# Association of female reproductive tract microbiota with egg production in layer chickens

**DOI:** 10.1093/gigascience/giab067

**Published:** 2021-09-23

**Authors:** Yuan Su, Shilin Tian, Diyan Li, Wei Zhu, Tao Wang, Shailendra Kumar Mishra, Ranlei Wei, Zhongxian Xu, Mengnan He, Xiaoling Zhao, Huadong Yin, Xiaolan Fan, Bo Zeng, Mingyao Yang, Deying Yang, Qingyong Ni, Yan Li, Mingwang Zhang, Qing Zhu, Mingzhou Li

**Affiliations:** Farm Animal Genetic Resources Exploration and Innovation Key Laboratory of Sichuan Province, Sichuan Agricultural University, Chengdu 611130, China; Department of Ecology, Hubei Key Laboratory of Cell Homeostasis, College of Life Sciences, Wuhan University, Wuhan 430072, China; Novogene Bioinformatics Institute, Beijing 100000, China; Farm Animal Genetic Resources Exploration and Innovation Key Laboratory of Sichuan Province, Sichuan Agricultural University, Chengdu 611130, China; Farm Animal Genetic Resources Exploration and Innovation Key Laboratory of Sichuan Province, Sichuan Agricultural University, Chengdu 611130, China; Farm Animal Genetic Resources Exploration and Innovation Key Laboratory of Sichuan Province, Sichuan Agricultural University, Chengdu 611130, China; Farm Animal Genetic Resources Exploration and Innovation Key Laboratory of Sichuan Province, Sichuan Agricultural University, Chengdu 611130, China; Center of Precision Medicine, West China Hospital, Sichuan University, Chengdu 610065, China; Farm Animal Genetic Resources Exploration and Innovation Key Laboratory of Sichuan Province, Sichuan Agricultural University, Chengdu 611130, China; Farm Animal Genetic Resources Exploration and Innovation Key Laboratory of Sichuan Province, Sichuan Agricultural University, Chengdu 611130, China; Farm Animal Genetic Resources Exploration and Innovation Key Laboratory of Sichuan Province, Sichuan Agricultural University, Chengdu 611130, China; Farm Animal Genetic Resources Exploration and Innovation Key Laboratory of Sichuan Province, Sichuan Agricultural University, Chengdu 611130, China; Farm Animal Genetic Resources Exploration and Innovation Key Laboratory of Sichuan Province, Sichuan Agricultural University, Chengdu 611130, China; Farm Animal Genetic Resources Exploration and Innovation Key Laboratory of Sichuan Province, Sichuan Agricultural University, Chengdu 611130, China; Farm Animal Genetic Resources Exploration and Innovation Key Laboratory of Sichuan Province, Sichuan Agricultural University, Chengdu 611130, China; Farm Animal Genetic Resources Exploration and Innovation Key Laboratory of Sichuan Province, Sichuan Agricultural University, Chengdu 611130, China; Farm Animal Genetic Resources Exploration and Innovation Key Laboratory of Sichuan Province, Sichuan Agricultural University, Chengdu 611130, China; Farm Animal Genetic Resources Exploration and Innovation Key Laboratory of Sichuan Province, Sichuan Agricultural University, Chengdu 611130, China; Farm Animal Genetic Resources Exploration and Innovation Key Laboratory of Sichuan Province, Sichuan Agricultural University, Chengdu 611130, China; Farm Animal Genetic Resources Exploration and Innovation Key Laboratory of Sichuan Province, Sichuan Agricultural University, Chengdu 611130, China; Farm Animal Genetic Resources Exploration and Innovation Key Laboratory of Sichuan Province, Sichuan Agricultural University, Chengdu 611130, China

**Keywords:** microbiota, reproductive tract, egg production, chicken

## Abstract

**Background:**

The microbiota of the female reproductive tract is increasingly recognized as playing fundamental roles in animal reproduction. To explore the relative contribution of reproductive tract microbiomes to egg production in chickens, we investigated the microbiota in multiple reproductive and digestive tract sites from 128 female layer (egg-producing) chickens in comparable environments.

**Results:**

We identified substantial differences between the diversity, composition, and predicted function of site-associated microbiota. Differences in reproductive tract microbiota were more strongly associated with egg production than those in the digestive tract. We identified 4 reproductive tract microbial species, *Bacteroides fragilis, Bacteroides salanitronis, Bacteroides barnesiae*, and *Clostridium leptum*, that were related to immune function and potentially contribute to enhanced egg production.

**Conclusions:**

These findings provide insights into the diverse microbiota characteristics of reproductive and digestive tracts and may help in designing strategies for controlling and manipulating chicken reproductive tract microbiota to improve egg production.

## Background

The domestic chicken (*Gallus gallus domesticus*, NCBI:txid9031) is of enormous agricultural significance, comprising broiler (meat-producing) and layer (egg-producing) chickens. Specialized commercial layer breeds were established during the twentieth century with greatly improved reproductive traits [1]. Currently, thousands of quantitative trait loci [2] and many gene mutations [3, 4] are reportedly associated with chicken reproductive traits. Nonetheless, egg production, as a polygenic inheritance trait, exhibits low to moderate heritability (*h*^2^, ranging from 0.05 to 0.44, depending on the period involved) [5, 6]. Alternative effective approaches for modulating egg production in laying hens are urgently required for the poultry industry to meet consumer demand.

Distinct bacterial communities throughout the female reproductive tract form a microbiota continuum from the vagina to the isthmus, which plays a prominent role in animal reproduction [7, 8]. In humans, microbiome interactions with the host during pregnancy leading to preterm birth were investigated [9], and temporal changes in the vaginal microbiome associated with full-term pregnancies were identified [10]. An abnormal vaginal microbiota may predispose individuals to increased microbial invasion of the amniotic cavity and fetal damage [11, 12]. The avian reproductive tract houses complex bacterial communities that are believed to play crucial roles in egg production [13]. Chicken digestive and reproductive tracts are mainly colonized by Firmicutes, Bacteroidetes, Proteobacteria, Actinobacteria, and Fusobacteria, which are spatially organized within specific digestive and reproductive compartments [14, 15]. Additionally, *Lactobacillus* species were found to be keystone species residing in the chicken oviduct [16].

Several synergistic factors, such as environment and diet, dominate over host genetics in determining gut microbiota composition [17, 18]. A comparative study of gut microbial diversity among parrot species indicated the potential role of host ancestry in shaping the gut microbiome [19]. A genome-wide association study (GWAS) in chickens demonstrated that the genetic loci rs15142709 and rs15142674, which are located in the pleiomorphic adenoma gene 1 (*PLAG1*) and lck/yes-related novel tyrosine kinase (*LYN*) genes, were significantly associated with microbial *Methanobacterium* abundance [20]. In a previous study, 14 identified quantitative trait loci strongly influenced *Clostridium leptum* and *Lactobacillus* abundance, as well as related candidate genes involved in anti-inflammatory responses and the motility of the digestive tract [21]. On the other hand, recent studies have suggested that host genetics have limited impact on gut microbiota composition in humans [22].

We speculated that the microbial component of the reproductive tract might be an important aspect of egg production in chicken. Here, we performed 16S recombinant DNA (rDNA) sequencing on 768 samples from 3 reproductive (vagina, uterus, and isthmus) and 3 digestive (crop, gizzard, and small intestine) tract sites and whole-genome sequencing of 128 laying hens. We characterized the reproductive tract microbiota and its features compared with those of the digestive tract microbiota of hens. We identified the contribution of key microorganisms to egg production and established a correlation between host genetics and the microbial diversity of 6 tract sites. These findings provide insights into the microbial communities in the reproductive tract of highly specialized layer populations, which may help in developing strategies to enhance commercial egg production.

## Results and Discussion

### Discriminative characteristics of microbiota in reproductive and digestive tract sites

The 16S rDNA sequencing in 768 samples generated a total of ∼57.61M high-quality reads (∼75.01k reads per sample). *De novo* clustering after singleton removal produced 46,480 operational taxonomic units (OTUs) at an identity cut-off of 97%, among which 6,776 OTUs found in >20% of samples were used for subsequent analysis (Supplementary Fig. S1a and b). We performed α-diversity analysis based on qualified sequencing depth with a mean Good coverage of 98.69% (range, 96.30–99.60%) (Supplementary Fig. S2a). Analysis of 5 indices (i.e., observed OTUs, ACE, Chao1, Simpson, and Shannon) (Supplementary Fig. S2b–f) indicated that the vast majority of pairwise comparisons between sites (80 of 90 pairwise comparisons [88.89%]) showed significant differences (*P* < 0.05, Wilcoxon rank-sum test), with the exception of 2 comparisons (uterus vs isthmus for all indices; and small intestine vs vagina for the observed OTUs, Simpson, and Shannon indices) (Supplementary Fig. S2g and h).

**Figure 1:**
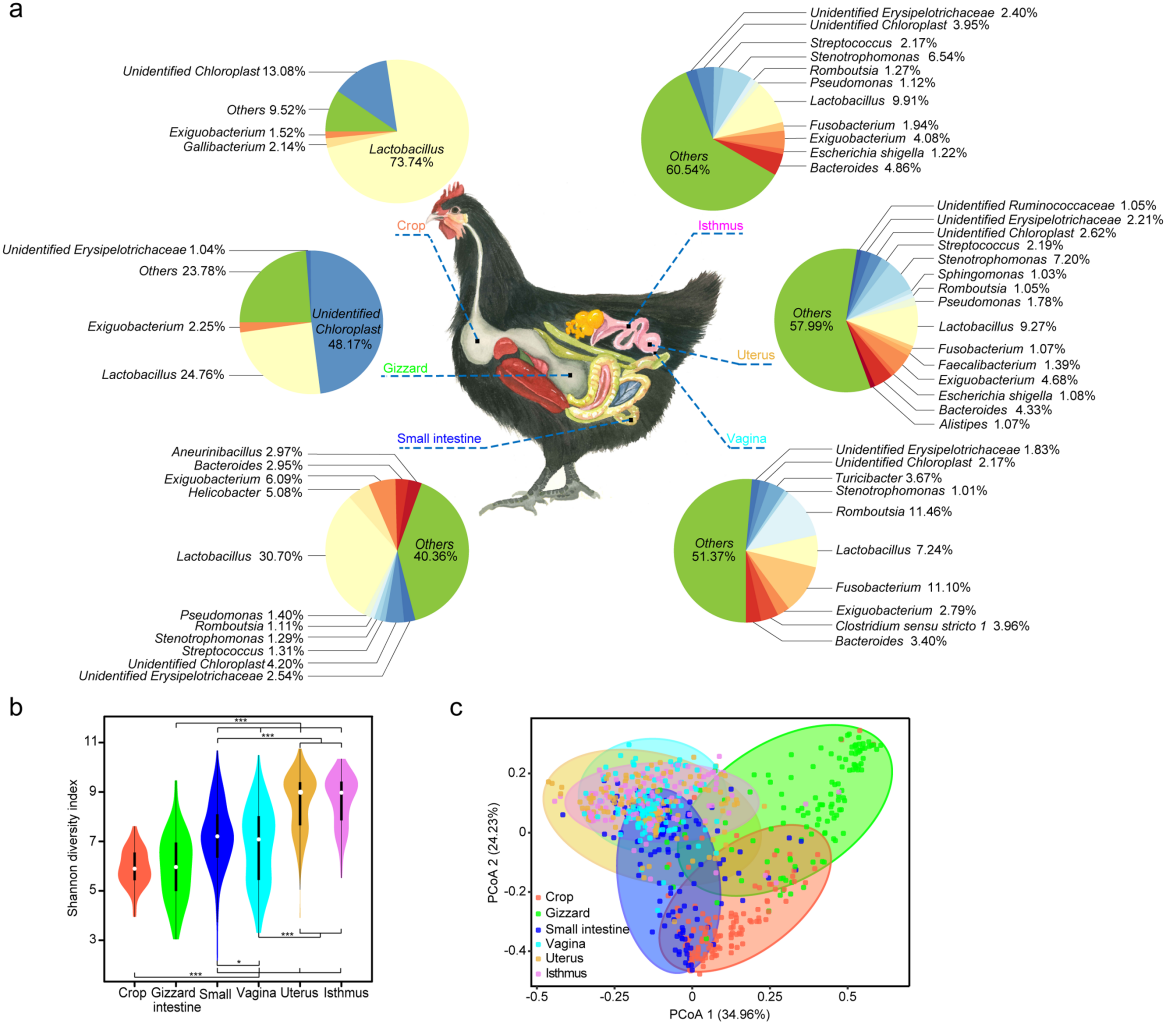
Diversity and composition of the reproductive and digestive tract microbiota in chickens. (**a)** Relative abundance of the microbiota from 6 sites at the genus level. Only genera with an abundance >1% in a site are shown. (**b)** α-diversity comparison based on the Shannon diversity index (*P < 0.05; **P < 0.01, Wilcoxon rank-sum test). Black boxes in the violin plots denote the interquartile range between the first and third quartiles (25th and 75th percentiles, respectively), and the centre white circle denotes the median value in the corresponding group, respectively. **(c)** Principal coordinates analysis of the 768 samples based on weighted UniFrac distances; 34.96% of variance was explained for component 1 (*P* < 0.05, Tracy-Widom test) and 24.23% for component 2 (*P* < 0.05, Tracy-Widom test).

Compared to the digestive system, the reproductive system exhibited higher α-diversity (all 5 indices) and thus contained more microbial taxa, especially in the upper reproductive tract (i.e., uterus and isthmus) (Fig. 1a and b). Similar to significant microbiota differences between the vaginal and upper reproductive tracts in humans [7, 23], we found highly discriminative microbial communities in chickens between the upper (isthmus and uterus) and lower (vagina) reproductive tracts (isthmus vs vagina, *R* = 0.473, *P* < 0.001, analysis of similarity [ANOSIM]; uterus vs vagina, *R* = 0.496, *P* < 0.001, ANOSIM) but indistinguishable microbiota between the isthmus and uterus (isthmus vs uterus, *R* = -0.003, *P* = 0.694, ANOSIM) (Supplementary Table S1). These results demonstrated microbiota heterogeneity throughout contiguous sites of the digestive and reproductive tracts in hens.

We used principal coordinates analysis (PCoA) to visualize differences in taxa composition between microbiota in the reproductive and digestive tracts. The first principal component, explaining 34.96% of the variance in weighted UniFrac distance matrices among the samples, separated reproductive and digestive tract samples (Fig. 1c). Given that reproductive and digestive tracts share a common exit in the cloaca, the frequently exchanged microbiome likely resulted in similar microbiota at the distal end of both tracts. Consequently, we found that the vagina acquired microbe communities from the isthmus and uterus, which all belong to the reproductive tract. Nonetheless, the vagina microbiota was partially indistinguishable from that of the small intestine (Fig. 1c). The unweighted UniFrac distance matrices (Supplementary Fig. S2i), weighted UniFrac distance (Supplementary Fig. S2j), and an ANOSIM based on Bray-Curtis (BC) distances (Supplementary Table S1) recapitulated these findings.

Similar phyla dominated the microbiota in the 6 sites; Firmicutes, Proteobacteria, and Cyanobacteria (likely an artifact of feed-derived chloroplast DNA) accounted for 71.45–97.86% of all OTUs. Nonetheless, some differences were observed among the sites. Cyanobacteria (likely an artifact of feed-derived chloroplast DNA) was the dominant material in the gizzard (48.19% of the total abundance); however, Firmicutes was the most abundant phylum (43.60–78.93%) in the other 5 sites. We also found that the uterus and isthmus had similar dominant phyla, including Firmicutes (44.87% and 43.60%), Proteobacteria (26.25% and 23.77%), and Bacteroidetes (17.13% and 19.52%) (Supplementary Fig. S2k). Strikingly, the vagina had the highest abundance of Fusobacteria (11.51%) among the 6 sites.

At the genus level, *Lactobacillus* (7.24%–73.74% in the 6 sites)*, Exiguobacterium* (2.79%–4.68%), *Stenotrophomonas* (1.01%–6.54%), and *Bacteroides* (3.40%–4.86%) were ubiquitously found across all sites with higher abundances than other bacteria owing to their broad adaptability and beneficial functions (Fig. 1a). We found *Lactobacillus* to be more dominant in the digestive tract (73.74% in crop, 24.76% in gizzard, 30.70% in small intestine) compared with the reproductive tract (7.24% in vagina, 9.27% in uterus, 9.91% in isthmus). *Lactobacillus* is thought to inhibit pathogenic bacteria by lowering the environmental pH through lactic acid and hydrogen peroxide production [24]. This genus was highly abundant in the digestive tracts, which were characterized by low pH values, which strongly limit the growth of most pathogens [25, 26]. In contrast, *Lactobacillus* was less abundant in the reproductive tract, where an alkaline pH is needed to maintain sperm motility [27, 28]. Unidentified Erysipelotrichaceae,unidentified chloroplast,*Lactobacillus*, and *Bacteroides* had abundances of >1.0% in the vagina, which was further increased in the uterus and isthmus (Fig. 1a).

Furthermore, 14.63% of genera (362 of 2,475) demonstrated associations between sites (*P* < 0.05 of Spearman *r, Z*-test) (Supplementary Fig. S2l). Typically, genera belonging to Proteobacteria and Firmicutes showed significantly positive correlations (*P* < 0.001, *Z*-test) between the crop and gizzard, the gizzard and small intestine, or the 3 reproductive tract sites. These results imply a connection of microbiome communities, possibly caused by the flow of material from different sites.

### Site-associated microorganisms in reproductive and digestive tracts

**Figure 2:**
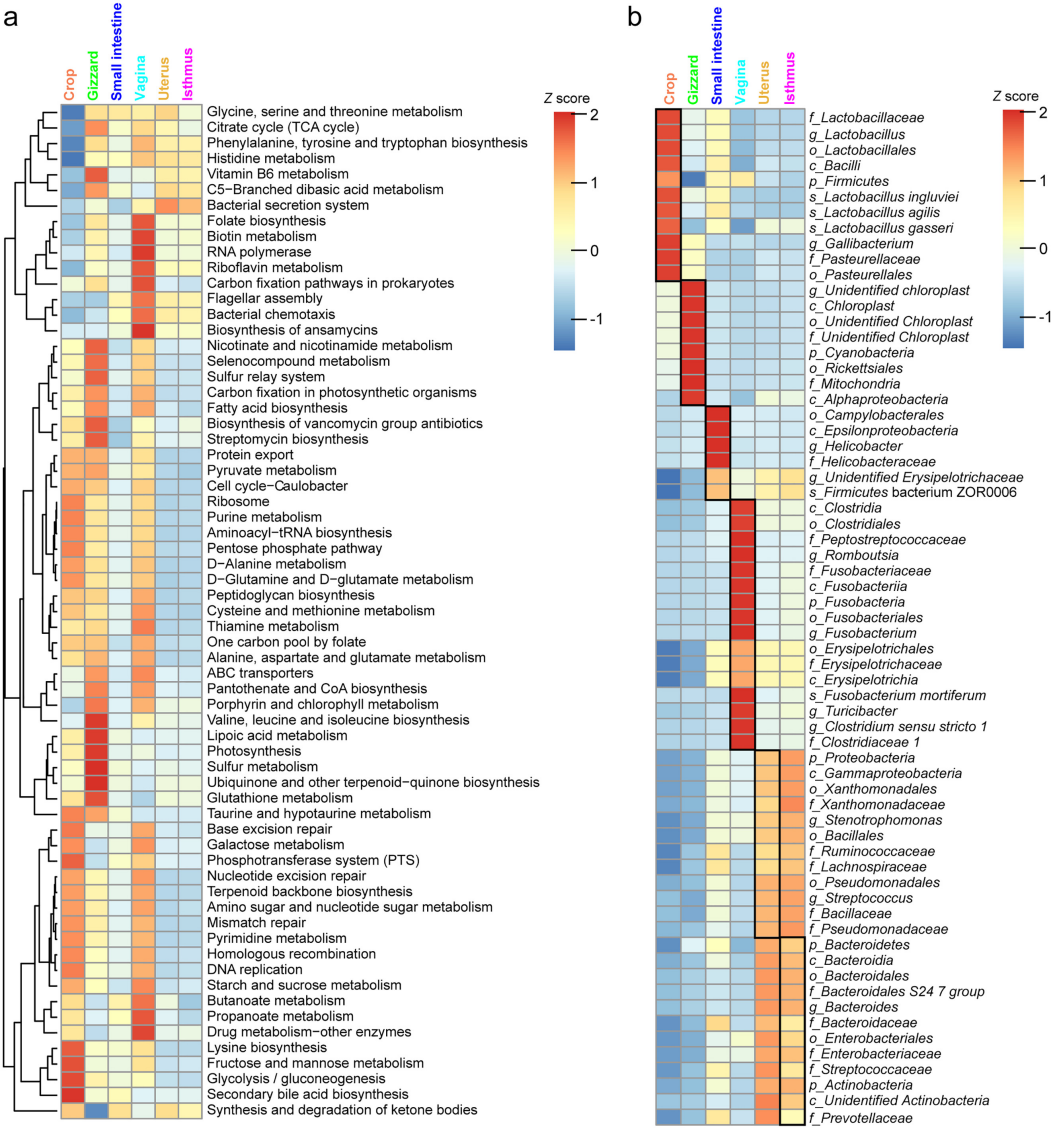
Comparison of predicted functional capacities and site-associated taxa of microbial communities. (**a)** Heat map showing the predicted KEGG pathways and their abundances at reproductive and digestive tract sites (Supplementary Table S2). *Z* scores indicate the means of KEGG pathway abundances. (**b)** Heat map showing the 65 site-associated bacterial taxa identified by LEfSe (LDA > 4). *Z* scores indicate the relative abundances of site-associated bacterial taxa. Black frames represent site-associated bacterial taxa whose *Z* scores of relative abundances differed significantly among the 6 sites. p: phylum; c: class; o: order; f: family; g: genus; tRNA: transfer RNA.

We analyzed the functional capacity of the microbiota in each reproductive and digestive tract site using PICRUSt2 and found that 72% of the representative pathways (36 of the top 50 KEGG pathways) were shared across the 6 sites, one-third of which (12 of 36) were primarily involved in metabolism (Supplementary Fig. S3a and b). Specifically, “bacterial secretion system” and “bacterial chemotaxis” were enriched in the reproductive tract. Previous studies found that successful bacterial pathogens evolved versatile protein secretion systems to promote their survival and fitness in response to different environmental challenges and to modulate host immunity [29–32]. Seven pathways were specifically enriched at a site (3 of 6 were site-specific to crop and gizzard, and “riboflavin metabolism” was specific to vagina). Abundances of the OTUs involved in these pathways differed among the 6 sites (*P* < 0.001, Wilcoxon rank-sum test) (Supplementary Table S2). For example, the microbial community of the small intestine had important roles in “valine, leucine, and isoleucine biosynthesis,” as indicated by the moderate row *Z* scores (−0.66) for each pathway. Moreover, “propanoate metabolism” (*Z* score = 1.72) and “bacterial chemotaxis” (*Z* score = 1.53) were overrepresented in the vagina. Meanwhile, “bacterial secretion system” was overrepresented only in the uterus (*Z* score = 1.29) and isthmus (*Z* score = 0.94) compared with the vagina and the 3 digestive tract sites (Fig. 2a).

We next identified 65 site-associated bacterial taxa among the 6 sites using linear discriminant analysis (LDA) effect size (LEfSe) [33] (Fig. 2b and Supplementary Fig. S3c), which confirmed most of the aforementioned observations (i.e., the uterus and isthmus showed essentially similar microbiota). Of note, *Helicobacter* and unidentified Erysipelotrichaceae, which were associated with the small intestine, showed the highest abundance among the 6 sites (Fig. 2b). Six genera from Lactobacillaceae were crop-associated bacteria. In chicken, the crop acts as a reservoir for the storage of food prior to its digestion, where food mixes with many beneficial *Lactobacillus* bacteria (73.84% at the genus level) that produce lactic acid before moving on to the proventriculus [34]. Next, the gizzard grinds any remaining large food particles with the assistance of grit, releasing abundant unidentified chloroplast (2.94%) and mitochondria-like (2.37%) materials from plant consumption. The small intestine exhibited the most abundant microbes of the 3 digestive tract sites; this is mainly where further digestion occurs and fermentation begins. Paenibacillaceae species, with optimum growth at pH 6.0–7.0, were also overrepresented. As a possible pathogen, *Helicobacter* specifically inhabits the small intestine in chickens and may be involved in inflammation, metabolism, and neutralization of gastric acid [35–37].

Unidentified Erysipelotrichaceae showed higher abundance in the 3 reproductive tract sites (1.83–2.40%). Bacteria associated with the isthmus and uterus both showed higher abundances than in the other sites. Several genera (typically, *Romboutsia, Fusobacterium*, and *Clostridium sensu stricto 1*) were dominant in the vagina (>25% of the microbiota) but had lower abundances in the other sites (Fig. 2b). Among these, vaginal *Romboutsia* could be used as a predictor for egg number in laying hens [8]. Six Bacteroidetes bacterial taxa were isthmus-associated; *Bacteroides* species live on host mucus-secreted polysaccharides, and this flexible foraging behavior contributes to diversity and stability [38].

These findings confirm that the digestive and reproductive tract microbiota in chicken is primarily determined by the physiological function of each compartment within these systems.

### Weak association between host genetics and microbial communities

To explore the relationship between host genome and microbiome of 6 sites, we generated a total of 1.76 Tb of high-quality genome sequences from 128 chickens with ∼10.15-fold mean depth for each individual (Supplementary Table S3) and identified a total of 10.82M single-nucleotide polymorphisms (SNPs) with a density of ∼10.29 SNPs per kb.

**Figure 3:**
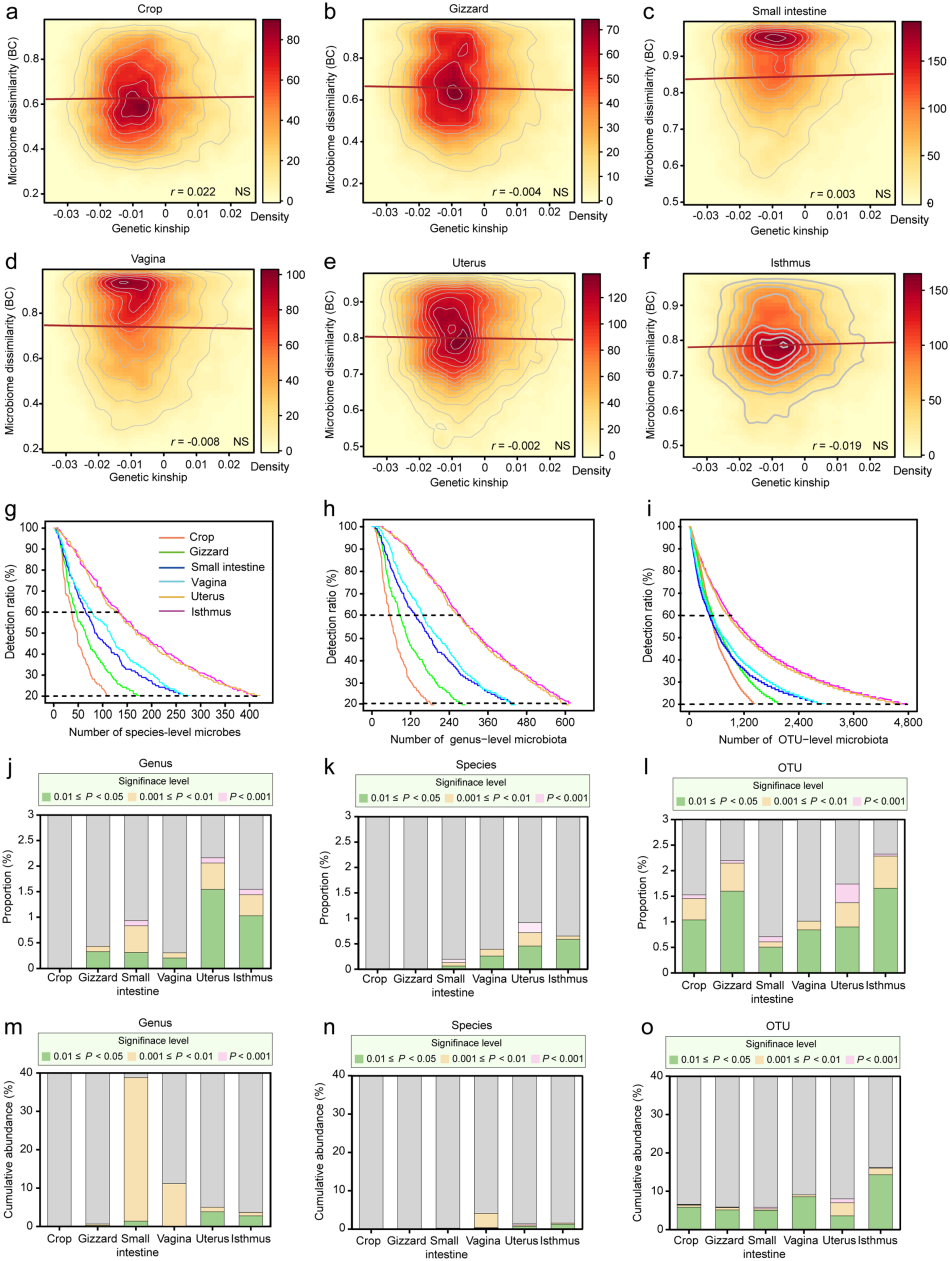
Association of host genetics with microbiota of the reproductive and digestive tracts. (**a–f)** Density scatter plots of genetic kinship of pairs of individuals (x axis) and their microbiome dissimilarity (y axis) among all pairs of individuals (*n* = 16,256). NS: not significant (*P* > 0.05; Mantel test). (**g–i)** Distribution of species, genera, and OTUs identified in 6 sites for all chickens. Microorganisms present in <20% of samples were excluded. (**j–l)** Proportion of heritable microbial genera, species, and OTUs in each site. **(m–o)** Cumulative relative abundances of heritable microbial genera, species, and OTUs in each site.

The correlation between host genetics (using genetic relatedness matrix [GRM] and microbial β-diversity based on BC distance) at the 6 sites in the same cohort of laying hens was not statistically significant (|*r*| < 0.033, *P* > 0.05, Mantel test, Fig. 3a–f and Supplementary Table S4). Nonetheless, the microbiomes of anatomically neighboring sites were similar. Typically, the microbial communities of the isthmus were positively correlated with those of the neighboring uterus (Spearman *r* = 0.426, *P* < 0.0001, Mantel test) but not significantly associated with the relatively distant crop (Spearman *r* = 0.019, *P =* 0.335, Mantel test, Supplementary Table S4). We also estimated the association between GRM and microbial relationship matrix (MRM) and obtained similar results: both Pearson and Spearman correlations suggesting that host genetics and the microbiota composition are weakly associated (Supplementary Table S4).

We next regarded the abundance of each microorganism as a quantitative trait to estimate the *h*^2^ of each microorganism at the species, genus, and OTU level. Microorganisms in >20% but <60% of samples were analyzed qualitatively as dichotomous traits (Fig. 3g–i). At the species and genus levels, no significant correlation (*P* > 0.05, Wilcoxon rank-sum test) was found between the presence of an SNP and the presence of a specific microbe (Fig. 3j–l) in the crop. Three species in the small intestine (accounting for 0.21% of the microbiota species tested in the small intestine), 6 in the vagina (0.39%), 14 in the uterus (0.92%), and 10 in the isthmus (0.66%) exhibited significant SNP-based heritability (*P* < 0.05, Wilcoxon rank-sum test) (Fig. 3k and Supplementary Table S5). Most of these heritable bacteria belonged to the Firmicutes phylum (Supplementary Fig. S4a, c, and e). Reproductive tract sites had more heritable bacterial phyla than digestive tract sites (Supplementary Fig. S4b, d, and f). The cumulative abundances of these heritable bacteria were only 0.22%, 4.14%, 1.46%, and 1.61% (*P* < 0.05, Wilcoxon rank-sum test) in the small intestine, vagina, uterus, and isthmus, respectively (Fig. 3n and Supplementary Table S5). Similar results were observed at the genus and OTU levels (Fig. 3m and o). These results supported that host genetics have limited effect on shaping the microbial composition of the reproductive and digestive tracts.

### Heritability (*h*^2^) and microbiability (*m*^2^) of EN300

To further explore the effect of genome and microbiome on egg number at 300 days of age (EN300), we used a GRM of sample pairs to estimate the *h*^2^ value of EN300 explained by whole-genome SNPs using the restricted maximum likelihood method. We found that EN300 exhibited relatively low to medium heritability (*h*^2^ = 0.282, *P* = 0.048, likelihood ratio test), which was comparable to previous estimations (Supplementary Table S6) [5, 6]. The fraction of EN300 variance explained by microbial variance was measured by microbiability (*m*^2^) [18]. After correcting for host genetic factors using EN300-related SNPs as additional covariates, we found that the estimated EN300 *m*^2^ values for digestive tract sites (0.523 for small intestine, 0.869 for crop, and 0.873 for gizzard) were lower than those for reproductive tract sites (0.923 for vagina, 0.936 for uterus, and 0.989 for isthmus) (Table 1). Generally, higher EN300 *m*^2^ values were observed for sites neighboring the ovaries; the isthmus was the most pertinent site with respect to egg production. Commercial egg producers are acutely interested in hen oviducts because pathological changes or disrupted activity directly affect egg production efficiency and ultimately decrease economic profitability [39]. In chickens, the inner and outer shell membranes form in the isthmus, while calcification of the eggshell, subsequent pigmentation, and cuticle deposition occur in the uterus and are followed by expulsion of the egg through the vagina [40]. These results suggest that EN300 in layer chickens is determined more by the microbiota in the reproductive tract than by those in the digestive tract.

**Table 1:** Estimated microbiability (*m*^2^) of EN300

Factor	Site	*m* ^2^ (SE)	*P* value
Digestive tract	Crop	0.869 (0.049)	<10^–16^
	Gizzard	0.873 (0.045)	<10^–16^
	Small intestine	0.523 (0.111)	2.56 × 10^–11^
Reproductive tract	Isthmus	0.989 (0.011)	<10^–16^
	Uterus	0.936 (0.030)	<10^–16^
	Vagina	0.923 (0.028)	<10^–16^
Host genetics		0.282 (0.231)	0.049

SE: standard error.

### Microorganisms in the reproductive tract are significantly associated with EN300

**Figure 4:**
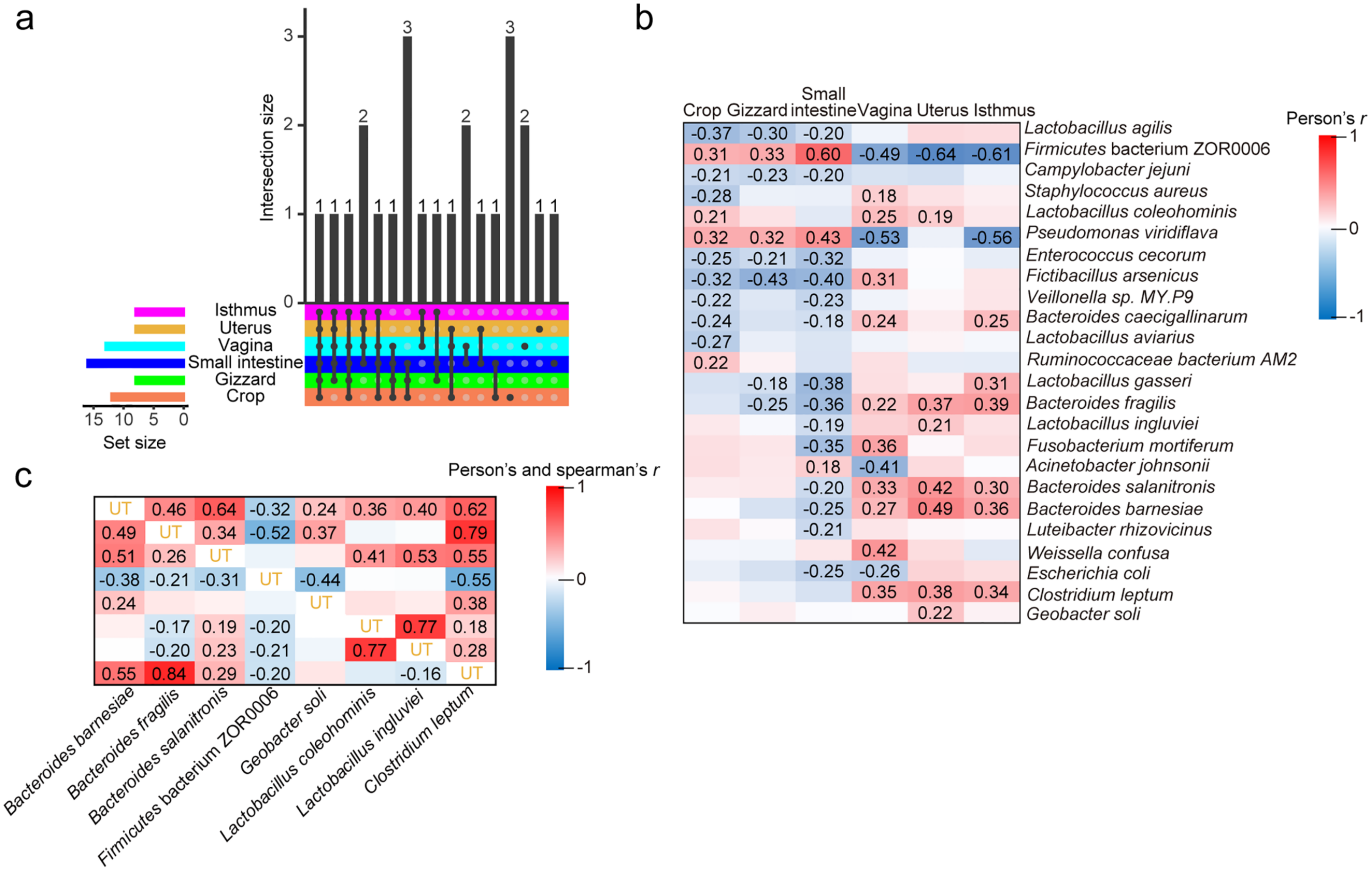
Microorganisms associated with egg number at 300 days of age (EN300). (**a)** Microbial species associated with EN300 (*P* < 0.05) among the 6 sites. **(b)** Pearson *r* values between EN300 and 24 EN300-associated microbial species; only significant *r* values are given numerically (*P* < 0.05). **(c**) Pearson *r* (lower diagonal) and Spearman *r* (upper diagonal) values among microbial species in the uterus (UT); significant *r* values are given numerically (*P* < 0.05).

We next focused on the microorganisms that are highly associated with EN300. The results showed that most of the microorganisms detected at the microbial species, genus, and OTU levels that were significantly associated with EN300 belonged to the Firmicutes phylum (*P* < 0.05, Wilcoxon rank-sum test) (Supplementary Fig. S5). Only microorganisms that exhibited a significant correlation between egg production and relative abundance as determined by both Pearson *r* and Spearman *r* were considered a causal relationship (*P* < 0.05, Wilcoxon rank-sum test). Consequently, 39 OTUs, 26 genera, and 24 species fulfilled these criteria (Fig. 4a and Supplementary Fig. S6a).

Most OTUs, genera, and species present in the 3 digestive tract sites were negatively correlated (Pearson *r*) with egg production (negative/positive: 19/6, 8/8, and 16/5, respectively), whereas they were positively correlated with egg production in the reproductive tract sites (positive/negative: 13/9, 11/10, and 13/4, respectively) (Fig. 4b and Supplementary Fig. S6b and c). Microorganisms in the uterus were most strongly correlated with each other (Fig. 4c and Supplementary Fig. S7), which implied a strong symbiotic/competitive relationship.

At the genus level, *Lactobacillus, Bacteroides*, and *Desulfovibrio* were positively correlated with EN300 in the 3 reproductive tract sites. *Pseudomonas, Exiguobacterium*, and unidentified Erysipelotrichaceae were negatively correlated with EN300 in the 3 reproductive tract sites but were positively correlated in the 3 digestive tract sites (Supplementary Fig. S6b). In accordance with previous findings, there is clear evidence of the role of fecal microbiomes in low and high egg-laying performance in hens; Elokil et al. [16] demonstrated a significantly positive association between the microbial genus *Lactobacillus* and egg-laying performance (*P* < 0.05). Likewise, Wang et al. [41] reported that *Lactobacillus* was also abundant in the feces of high-yield hens. The genus *Lactobacillus* produces growth promoters and exhibits antimicrobial activity against pathogenic microbes [42, 43], which may explain why the increasing abundance of *Lactobacillus* in the high-yield group is beneficial to egg-laying performance. The cecum has distinct microbial community profiles [44–46] that were not explored in this study. Microbial community analysis of the cecum microbiota in chickens exhibiting different egg production performance requires further investigation.

At the species level, *Bacteroides fragilis, Bacteroides salanitronis, Bacteroides barnesiae*, and *Clostridium leptum* were positively correlated with EN300 in the 3 reproductive tract sites but were weakly negatively correlated with EN300 in the small intestine, while no correlation was found in the crop and gizzard. The first 3 species belong to the genus *Bacteroides*, which had a significantly positive correlation with egg production in the 3 reproductive sites (Pearson *r* = 0.403–0.479). *Bacteroides* species have been identified as the predominant anaerobic genera in chicken cecum [47], which were thought to play an important role in the breakdown of polysaccharides into simpler compounds used by the animal host as well as the microorganisms themselves [48]. Interestingly, a recent study reported that the human-adapted *Bacteroides* species are likely introduced to chicken flocks by contact with humans and can temporarily persist in chickens [49]. Intestinal anaerobic bacteria such as *B. fragilis* and *B. salanitronis* have been suggested to possess metabolic pathways for N-glycan production [50]. The symbiont *B. fragilis* exists in a commensal relationship with the host as it expresses a relatively large number of genes involved in polysaccharide metabolism, which benefits the host. The surface of *B. fragilis* can produce polysaccharides; in particular, capsular polysaccharide A (CPSA) is a key mediator of mammalian immune system development [51]. Surprisingly, CPSA has also been shown to exert protective effects in autoimmune disorder models, such as antibiotic-induced experimental encephalomyelitis. It is thus suggested that the genus *Bacteroides* could regulate reproductive activity by mediating the avian immune system.

**Figure 5:**
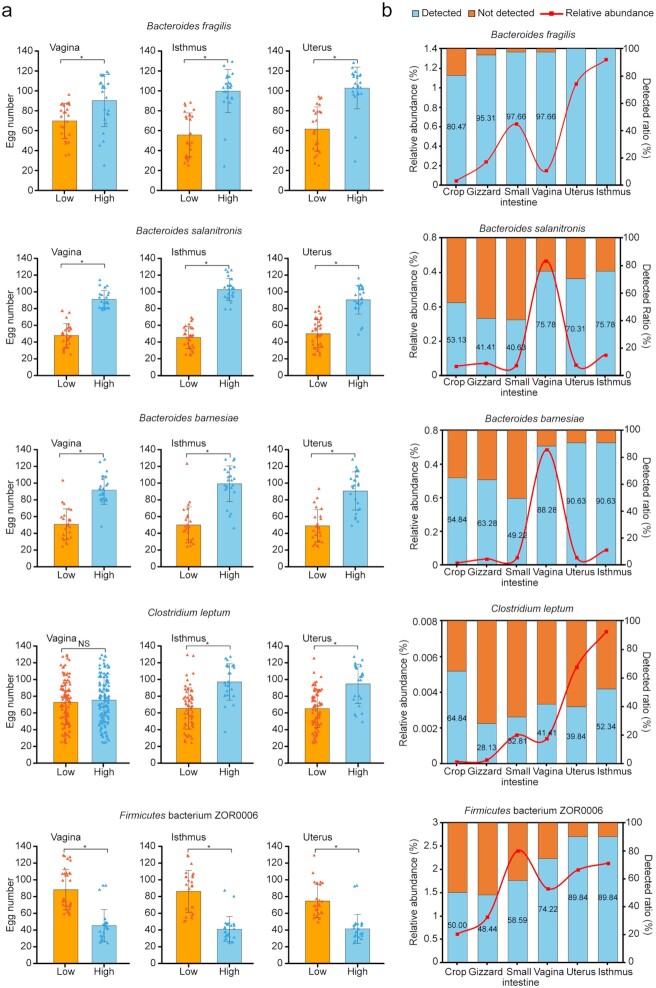
Effect of microbial species associated with EN300. (**a)** EN300 values for the 20% of chickens with the highest and lowest abundances of *B. fragilis, B. salanitronis, B. barnesiae, C. leptum*, and Firmicutes bacterium ZOR0006 in the 3 reproductive tract sites.Data plotted are mean ± standard error in the corresponding group, respectively. Significance levels were calculated using a permutation test with 10,000 replicates. *P < 0.05; NS, not significant. (**b)** Relative abundance and detected ratio of 5 species (*B. fragilis, B. salanitronis, B. barnesiae, C. leptum*, and Firmicutes bacterium ZOR0006) in the 6 sites. Blue bars indicate the detection ratio of each species at each site.

Firmicutes bacterium ZOR0006 had a significantly negative correlation with EN300 in the 3 reproductive tract sites and a significantly positive correlation in the 3 digestive tract sites. The 20% of chickens with the lowest EN300 values (mean = 37.13) had significantly lower *B. fragilis, B. salanitronis, B. barnesiae*, and *C. leptum* abundances (*P* < 0.05, Wilcoxon rank-sum test) (Supplementary Fig. S8a and b) compared with the 20% of chickens with the highest EN300 values (mean = 113.75) in the reproductive tract sites. Although its function is unknown, the 20% of chickens with the highest abundance of Firmicutes bacterium ZOR0006 exhibited significantly lower EN300 values than the 20% of chickens with the lowest abundance of this microorganism (Fig. 5a) (*P* < 0.05, Wilcoxon rank-sum test) in the reproductive tract sites.

Moreover, the 20% of chickens with the highest *B. fragilis, B. salanitronis, B. barnesiae*, and *C. leptum* abundances exhibited significantly higher EN300 values than the 20% of chickens with the lowest abundances of these microorganisms in the reproductive tract sites, with the exception of *C. leptum* abundance in the vagina (Fig. 5a). *C. leptum*, a major member of the Firmicutes phylum, can alter the gut microbiota in rats, especially in obese individuals. In human infants, fecal levels of *C. leptum* were found to be negatively correlated with proinflammatory marker levels [52]. Colonic colonization of *C. leptum* was associated with accumulation of regulatory T cells, which inhibited the development of inflammatory lesions. The proliferation and activation of regulatory T cells is crucial to establishing and maintaining an appropriate level of immune tolerance. In addition, our results demonstrated that *C. leptum* was associated with a large range of other uterus or isthmus microbiota constituents (but limited association was observed with digestive microbiota constituents) and was not influenced by host genetics. Thus, this microorganism might serve as a stimulator of regulatory T cell production and inhibitor of inflammatory lesions, then regulating and maintaining immunologic tolerance and microbiota composition of the reproductive tract (especially the uterus and isthmus). These results suggest that the microbial species contributing to the enhanced egg production are modulated by influencing the immune processes.

We then characterized the spatial distribution of these 5 EN300-associated microorganisms (*B. fragilis, B. salanitronis, B. barnesiae, C. leptum*, and Firmicutes bacterium ZOR0006). *B. fragilis* was detected in almost all samples and accounted for 0.05–1.29% of the total abundance (Fig. 5b). *B. salanitronis* and *B. barnesiae* were detected at similar ratios in the 6 sites and in most samples from the reproductive tract sites; both accounted for the highest abundance in the vagina. Firmicutes bacterium ZOR0006 was also detected in most samples from the reproductive tract sites (74.22–89.84%) and in half of the samples from the digestive tract sites (48.44–58.59%), accounting for 0.61–2.40% of the total abundance. Although the detection ratio (28.13–64.84%) and relative abundance of *C. leptum* were much lower than those of other microorganisms in all 6 sites, they accounted for the highest abundance in the isthmus and uterus (Fig. 5b).

Multiple factors, especially host species, potential pathogens, and immune status of the host, all play a major role in the female reproductive organs adversely interfering with the egg industry in laying flocks [53]. Additionally, the digestive tract environment of low-producing laying hens is fragile and susceptible to the influence of exogenous microorganisms [41]. Pathogenic infection, room temperature fluctuations, management systems, and other sudden changes to various factors can alter the composition of microbiota [54, 55]. These alterations may cause a significant degradation in production performance. Here, our results indicate that the reproductive tract microbiota plays an important role in egg production.

### Transcriptomic divergence in the uterus between hens with high and low egg production

**Figure 6:**
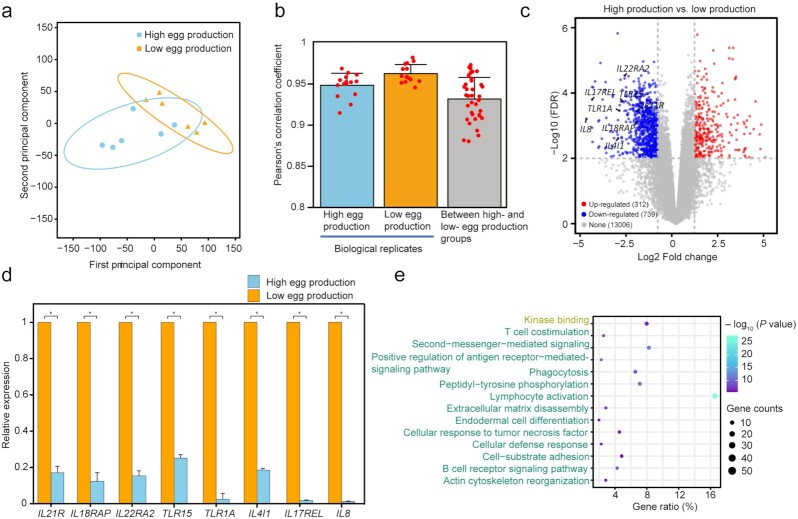
Microorganisms influence the expression of immune-related genes in the uterus. **(a)** Hierarchical clustering and (**b)** pairwise Pearson correlations of 12 samples using transcriptional profiles. The error bars indicate the standard error values in the corresponding group, respectively. **(c)** Differentially expressed genes between groups with high and low egg production. Eight well-documented inflammatory markers are labeled. (**d)** Comparison of expression changes of 8 inflammatory markers in the uterus between groups with high and low egg production by a q-PCR approach. Data are shown as the mean ± standard error. ^*^P < 0.05. **(e)** Top 20 functional categories enriched for 739 genes that were significantly downregulated in the high egg production group. The enrichment analysis was performed using the Metascape tool (see Methods). GO-BP: biological process (blue); GO-MF: molecular function (yellow).

Furthermore, we compared the transcriptional profiles in the uterus between the 2 groups composed of hens with either the 20% lowest or 20% highest EN300 values (6 hens for each group). As expected, the correlation rates between the high and low egg production groups (mean Pearson *r* = 0.93) were relatively lower than those between biological replicates (mean Pearson *r* = 0.95 and 0.96 for groups with high and low egg production, respectively) (Fig. 6a and b), indicating significant biological differences between groups. We identified 1,051 genes that exhibited significant expression changes (false discovery rate [FDR] ≤ 0.01 and |log_2_ (fold change)| ≥ 1) between groups with high and low egg production (Fig. 6c), which are mainly involved in immune-related categories, including the “NF−κB signaling pathway” and “chronic inflammatory response” (Supplementary Fig. S9). Of these, 739 genes that were significantly downregulated in the high egg production group were overrepresented in the categories related to the inflammatory response, including “T cell costimulation,” “B cell receptor signaling pathway,” and “lymphocyte activation” (Fig. 6e). Notably, we observed 8 well-documented inflammatory markers (2 Toll-like receptors [TLR15 and TLR1A] and 6 interleukins [IL21R, IL18RAP, IL22RA2, IL4I1, IL17REL, and IL8]) (Fig. 6c and d) that were significantly differently expressed in the uterus of the high egg production group and the low egg production group. Functionally, the microbiota of the uterus affects the health of the oviduct and thus influences chicken egg production, which is manifested as increased pathway abundance for bacterial motility proteins, the bacterial secretion system, and membrane and intracellular structural molecules.

## Conclusion

Our study provides a comprehensive view of the microbial community in the digestive and reproductive tracts of layer chickens. The diversity, composition, and predicted function of the microbiota varied considerably according to location within the reproductive and digestive tracts. Our results indicate that the reproductive tract microbiota in the hen influences egg production more than the digestive tract microbiota, and host genome has limited effect on their microbial composition. A small proportion of the variability in egg production was associated with the microbiota in the reproductive and digestive tracts of chickens. Remarkably, the genus *Bacteroides* and the species *C. leptum* and Firmicutes bacterium ZOR0006 were strongly associated with egg production, indicating their potential role in promoting reproductive performance. These findings provide new insight into the roles of reproductive and digestive tract microbiota for complex traits and may help contribute to the development of effective therapies for improving commercial egg production in chickens.

## Methods

### Chickens

The study was conducted on a common flock of 128 Dongxiang green shell laying hens reared on an experimental poultry farm at Sichuan Agricultural University in Ya'an, Sichuan, China. All chicks were hatched on the same day and housed in individual pens. Feed intake was controlled daily according to standard farm husbandry practices and water was provided *ad libitum*. The number of eggs produced for the first 300 days of life was recorded daily for each individual. We determined that the mean number of eggs (∼75.32; range, 24–129) that each hen laid by 300 days of age (EN300) fit a normal distribution pattern (*P* = 0.725, Kolmogorov-Smirnov test) (Supplementary Fig. S10). At the age of 300 days, 2 mL of whole blood was collected from the wing vein using venipuncture and stored at −20°C. Subsequently, each individual was culled by cervical dislocation followed by decapitation. After the abdomen was opened, fresh tissue was collected from 3 sites in the reproductive tract (vagina, uterus, and isthmus) and 3 sites in the digestive tract (crop, gizzard, and small intestine) (Fig. 1a). A 12-cm-long fixed mid-region of the small intestine (jejunum) was collected from each bird. All samples were snap-frozen in liquid nitrogen, transported to the laboratory, and stored at −80°C until further analysis. Chickens were managed according to the Institutional Animal Care and Use Committee of Sichuan Agricultural University under permit No. DKY- 2,018,102,015.

### Host and microbial genomic DNA extraction

Host DNA was isolated from blood using a TIANamp Genomic DNA Kit (Tiangen Biotech, Beijing, China) following the manufacturer's instructions. Total microbial genomic DNA from lumen of digestive tracts and mucus of reproductive tracts were extracted from ∼200 mg tissues using a TIANamp Stool DNA Kit (Tiangen Biotech) according to the manufacturer's instructions. The extracted DNA was quantified using a NanoDrop2000 spectrophotometer (Thermo Fisher Scientific, Chengdu, China), and DNA integrity was determined by 1% agarose gel electro-phoresis.

### 16S rDNA amplicon and sequencing

The V4 hypervariable region of the bacterial 16S rDNA was amplified using Phusion^®^ High-Fidelity PCR Master Mix (New England Biolabs, Ipswich, MA, USA) and the universal primers 515 F (5′-GTGCCAGCMGCCGCGGTAA-3′) and 806 R (5′-GGACTACHVGGGTWTCTAAT -3′) [56]. Reactions were carried out using 15  μL of Phusion^®^ High-Fidelity PCR Master Mix, 3  μL of the forward and reverse primers, 10 μL of template DNA, and 2 μL of ddH_2_O; no template control was also performed. The PCR cycling conditions were as follows: an initial denaturation step at 98°C for 1 min and 30 cycles of 98°C for 10 sec, 50°C for 30 sec, and 72°C for 30 sec, followed by an extension step at 72°C for 5 min, using a Bio-Rad CFX96 thermal cycler (Bio-Rad Laboratories, Hercules, CA, USA). Amplicons were purified on agarose gel (1%) using a GeneJET Gel Extraction kit (Thermo Fisher Scientific, Schwerte, Germany). A DNA library was prepared using an Ion Plus Fragment Library Kit (Thermo Fisher Scientific) based on the manufacturer's instructions. Reads were barcoded per sample, combined for multiplexed sequencing with the Ion S5^TM^ XL platform (Thermo Fisher Scientific) to generate 400-bp single-end reads, and sequenced by Novogene Bioinformatics Technology Co. Ltd of China.

### Whole-genome sequencing

After qualified host DNA samples were tested, the DNA was randomly fragmented using an ultrasonicator (Covaris Inc., Woburn, MA, USA), and then a sequencing library was prepared using a TruSeq Nano DNA HT Sample Preparation Kit (Illumina, San Diego, CA, USA) following the manufacturer's instructions. Index codes were added to tag each sample. DNA fragments were then end-repaired, dA-tailed, and ligated with a full-length adapter for Illumina sequencing, PCR amplification, and purification. Next, isolated DNA libraries were constructed with an insert size of 350 bp. Finally, genomes of the 128 individuals were separately sequenced with 150-bp paired-end reads using the Illumina Novaseq platform by Novogene Bioinformatics Technology Co. Ltd.

### 16S rDNA-seq data processing

The raw data obtained by sequencing were first filtered to obtain high-quality data. First, the adapter sequences in 61.05 million (M) raw reads (Supplementary Table S7) were trimmed using Cutadapt (version 1.9.1) [57] when the overlap length between the read and the adapter was shorter than 10 bp. Then, barcode sequences were trimmed and reads that were too long (>260 bp) or too short (<220 bp) were filtered using Cutadapt with the parameters “-e 0 -q 17 -m 200 -M 2600.” Next, the remaining reads were compared with the ChimeraSlayer reference database using the UCHIME algorithm [58] to detect and then remove chimera sequences. Finally, low-quality reads (i.e., >50% of bases with a phred quality <5) were also removed. Consequently, 57.61 M high-quality reads were generated for subsequent analysis (Supplementary Table S8). Greater than 93.85% of the high-quality reads had lengths of 250–260 nt (Supplementary Fig. S11a). Data with a quality score >20 accounted for 88.14% of all the effective bases (Supplementary Fig. S11b and c). The error ratio of the sequencing reads was relatively high in the ending position (Supplementary Fig. S11d).

### OTU cluster and species annotation

The remaining high-quality sequences were used to generate OTUs by Uparse software (version 7.0.1001) [59] with an identity cut-off of 97%. Singleton OTUs (OTUs found in only 1 sample) that did not match the reference database were removed. Clustering across all samples from the 128 chickens produced 46,480 OTUs after singleton removal. Nonetheless, most of those OTUs were present in low abundance and were found in very few samples. We then discarded OTUs that were not found in ≥20% of the chickens in each sampling site, yielding 6,776 OTUs (Supplementary Fig. S1a and b). For each OTU, the SSUrRNA library in Silva (https://www.arb-silva.de/) [60] was used to annotate taxonomic information (i.e., kingdom, phylum, class, order, family, genus, and species) based on the Mothur algorithm [61]. Subsequently, we determined the phylogenetic relationship of different OTUs and dominant species differences in samples (groups) after multiple sequence alignment using MUSCLE software (version 3.8.31) [62]. Additionally, OTU abundance information was normalized using a standard sequence number corresponding to the sample with the fewest sequences.

### α-Diversity

We used α-diversity to analyze the complexity of species diversity for a sample based on normalized OTUs through 6 indices (i.e., observed OTUs, ACE, Chao1, Simpson, Shannon, and Good's coverage), using the QIIME2 software [63]. Among these, Chao1 and ACE were selected to identify community richness, Shannon and Simpson were used to identify community diversity, and Good's coverage was used to characterize sequencing depth. Differences in α-diversity indices among the 6 sites were calculated with the Wilcoxon rank-sum test using R software (version 2.15.3).

### β-Diversity

We used β-diversity to evaluate differences in samples. The β-diversity in BC and weighted/unweighted UniFrac distances were calculated using QIIME2 software [63]. The BC ordination provided position values along an ordination axis and distances from the axis for samples of communities.

### Principal coordinate analysis

PCoA was performed to obtain principal coordinates and to visualize complex, multidimensional data. A distance matrix of previously obtained weighted/unweighted UniFrac distances among samples was transformed to a new set of orthogonal axes, by which the maximum variation factor was demonstrated by the first principal coordinate, the second maximum variation factor was demonstrated by the second principal coordinate, and so on. PCoA was performed using the WGCNA package [64], stat packages, and ggplot2 package in R software.

### Prediction of the functional profiles of microbial communities

The functions of the microorganisms present in the microbial communities detected in the 6 sites were predicted using PICRUSt2 [65]. We used the Wilcoxon rank-sum test to investigate differences in pathways among sites. *P*-values were adjusted using the Benjamini-Hochberg method by the FDR with the p.adjust function in R.

### Community difference analysis

Pairwise comparisons between different sites were statistically compared using ANOSIM (also named permutational MANOVA) with 10,000 permutations based on BC ordination to evaluate the reasonability of the division of groups.

### Between-group variation analysis

High-dimensional biomarkers were discovered by LEfSe using the parameter “LDA score > 4” [33] to identify characteristics of abundance and related classes (e.g., genes, metabolites, or taxa).

### Identification of microbiota constituents related to egg production

EN300 values between 2 groups (the lowest- and highest-ranked 20% of chickens with respect to their EN300 value) were then compared using the Wilcoxon rank-sum test. Microorganisms with *P* < 0.05 and FDR < 0.05 were retained. Furthermore, we calculated the Spearman *r* and Pearson *r* between EN300 and the abundance of each microbiota constituent at genus, OTU, and species levels. A significant correlation between the presence of a microorganism and the EN300 value was considered if *P* < 0.05, as determined using the psych package in R with the *P* value adjusted using the Benjamini-Hochberg method. Overlapping microorganisms obtained from the Wilcoxon rank-sum test and Spearman *r* and Pearson *r* were considered to have a potential relationship with EN300. We subsequently characterized EN300-associated microbes in the 6 sites.

### Whole-genome sequencing data processing

To avoid analysis noise caused by sequencing errors, low-quality paired reads (reads with ≥10% unidentified nucleotides [nt]; >10 nt aligned to the adaptor, allowing ≤10% mismatches; >50% bases having phred quality <5; and putative PCR duplicates generated in the library construction process), which mainly resulted from base-calling duplicates and adaptor contamination, were removed using an in-house script [66]. Consequently, 1.30 Tb (∼10.15-fold per individual) of high-quality paired-end reads were obtained, including 95.13% and 88.98% nucleotides with phred quality ≥Q20 (with an accuracy of 99.00%) and ≥Q30 (with an accuracy of 99.90%), respectively (Supplementary Table S3).

### Read mapping, and genomic variant calling and annotation

The remaining high-quality reads of each individual were aligned to the reference chicken genome (Gallus_gallus-6.0 Ensembl release 98 [81]) using BWA (version 0.7.15) [67] with the command “mem -t 10 -k 32.” BAM alignment files were then generated using SAMtools (version 0.1.19) [67]. Additionally, we improved alignment performance through filtering the alignment reads with mismatches ≤5 and mapping quality = 0. After sorting by SAMtools, the sorted BAM file was marked in duplicate using the command “MarkDuplicates” in the package Picard (version 1.119).

Subsequently, we performed gVCF calling in accordance with the Genome Analysis Toolkit (GATK) best practices pipeline (version v3.7) [68] using the HaplotypeCaller-based method, and then population SNP calling by merging all gVCFs with the commands “CombineGVCFs.”

To obtain high-credibility SNPs, we applied the hard filter command “VariantFiltration” to exclude potential false-positive variant calls as follows: (i) quality by depth >10.0; (ii) mapping quality score >40.0; (c) FS <60.0; (d) MQRank-Sum >−12.5; (e) ReadPosRankSum >−8.0. In addition, we further removed the SNPs with adjacent distances ≤5 [69]. Finally, we used vcftools (version 0.1.15) to obtain biallelic variants with the following parameters: sample call rate >90%, SNP call ratio >95%, minor allele frequencies >1%, and Hardy-Weinberg equilibrium *P* value < 10^−5^. Ultimately, a total of 10.82 M high-credibility SNPs in 128 individuals were retained (Supplementary Table S9). SNPs were classified into different genomic regions (i.e., exonic, intronic, splice sites, upstream and downstream around gene regions, and intergenic) using the ANNOVAR package [70].

### Construction of microbial relationship and host genetic relatedness matrices

OTUs identified in each site were normalized to a zero mean and unit variance. We then constructed an MRM [71] using an R script based on the following equation:

\begin{eqnarray*}
r{_{tij}} = \frac{1}{{N{_T}}}\ \mathop \sum \nolimits_{o\ = \ 1}^{N{_T}} \frac{{\left( {a{\ _{tio}} - \overline {t{\ _{to}}} } \right)\left( {a{\ _{tjo}} - \overline {a{\ _{to}}} } \right)}}{{\sigma _{to}^2}}, \end{eqnarray*}

where $\ r{\ _{tij}}$ represents the tested microbial relationship in tract *t* between chickens *i* and *j*; $a{\ _{tio}}$ and $\ a{\ _{tjo}}$ are the abundance of OTU *o* in tract *t* in chickens *i* and *j*, respectively; $\ \overline {t{\ _{to}}} \ $ is the mean relative abundance of OTU *o* in tract *t* in the population; $\ \sigma _{to}^2\ $ is the variance in the abundance of OTU *o* in tract *t*; and *N_T_* is the total number of OTUs in tract *t* used for the computation of relatedness. High-quality SNPs were further used to detect independent markers using PLINK [72], with the following parameters: 50 kb window size, 10 SNPs per step, and 0.2 as a squared Pearson *r* (${r^2}$). All 10,809,968 SNPs were used to compute the principal components (PCs) and GRM [73] using GCTA version 1.91.1 [74]:

\begin{eqnarray*}
{h_{ij}} = \frac{1}{N}\ \mathop \sum \nolimits_{a\ = \ 1}^N \frac{{\left( {{r_{ia}} - 2\overline {{f_a}} } \right)\left( {{r_{ja}} - 2\overline {{f_a}} } \right)}}{{2\overline {{f_a}} \left( {1 - \overline {{f_a}} } \right)}}, \end{eqnarray*}

where *h_ij_* is the tested genetic relationship between chickens *i* and *j; r_ia_* and *r_j__a_* represent the number of reference alleles in chickens *i* and *j*, respectively; $\overline {{f_a}} $ is the frequency of the reference allele in the population; and *N* is the number of variants.

### Heritability (*h*^2^) analysis

To estimate the effects of host genetics on the microbiota at different sites, we computed the correlation between GRM and BC distances at each site using both Pearson *r* and Spearman *r*, based on Mantel tests with 10,000 permutations. The correlation between GRM and MRM was also computed. To estimate the correlation between GRM and the microbiota community, we computed heritability at OTU, genus, and species levels. OTU abundance information was normalized using a standard sequence number corresponding to the sample with the least number of sequences. Microorganisms that were present in <60% but ≥20% of the samples were dichotomized as present or absent [75], and the microorganisms that were detected in <20% of the samples from each site were excluded from the analysis.

### Genetic and microbial parameters of egg production

Because the individuals examined in this study had no pedigree information, we computed the SNP-based heritability of the egg production phenotype (i.e., EN300) instead, using the following model [73]:

\begin{eqnarray*}
y\ = {K_c}\ + g + e\left[ A \right], \end{eqnarray*}

where *y* is an observed value (EN300); *c* is a vector of fixed covariates with the corresponding design matrix *K; e* is the residual effect; and *g* is a vector of aggregate effects of all SNPs with an ∼*N*(0,${G_{\sigma _A^2}}$), where G and $\ \sigma _A^2$ are the GRM and polygenetic variance (overall SNP effects), respectively. The top 5 host genetic PCs were considered covariates in the model to account for the calculated population stratification, as described above. The likelihood ratio test *P* value was calculated to examine the significance of the association between SNPs and EN300.

The fraction of EN300 variance explained by microbial variance was calculated as ${m^2} = {\sigma _m^2}/{\sigma _p^2}$ (called “microbiability” [${m^2}$] in animals [71] and “microbiome-association index” in humans [18]), where $\sigma _m^2\ {\mathrm{and\ }}\sigma _p^2$ are the phenotypic variance and microbial variance, respectively. To adjust for host genetic effects, all valid individuals and SNPs were used in a GWAS with a univariate linear mixed model (LMM), which was performed using GEMMA [76]. The LMM was calculated as follows:

\begin{eqnarray*}
y\,=\,{K_c}\,+\,{m_s}\, +\, e[{\rm B}], \end{eqnarray*}

where the model parameters are the same as those described in model $[ {\mathrm{A}} ]$, except ${m_s}$, which is the random effect of the microbiota in locations following the multinomial distribution $\ {m_s}$∼*N*(0, *M*$\sigma _m^2$), and *M* is the MRM. We then used the MRM in GCTA to calculate ${m^2}$. The genome-wide significance threshold was 10^–6^. We then extracted these SNPs with significant effects on EN300 and calculated the PCs using PLINK. The first 2 PCs and the top 5 host genetic PCs were then used as covariates in model [B] to account for host genetics.

### RNA sequencing analysis

For RNA sequencing (RNA-seq), total RNA was extracted from uterine tissue of 12 hens (6 for each of the groups with either the 20% lowest or 20% highest EN300 values) using the RNeasy Mini Kit (Qiagen). We used an rRNA depletion protocol (Ribo-Zero kit, Epicenter) coupled with the Illumina TruSeq stranded RNA-seq library protocol to construct the RNA-seq libraries. A total of 12 libraries were quantified using the Qubit dsDNA High Sensitivity Assay Kit (Invitrogen) and separately sequenced on the NovaSeq 6000 platform (Illumina) to produce an average of ∼31.86 M 150-bp paired-end raw reads and ∼30.52 M high-quality reads for each library. Sequence reads were aligned to the chicken reference genome (Gallus_gallus-6.0 Ensembl release 98) by the STAR alignment tool (version 2.5.3a). On average, ∼96% of reads of individual libraries were aligned to the chicken reference genome, generating a mean of 29.30 M aligned reads for each sample. The gene expression level was then estimated as transcripts per million (TPM) using the high-speed transcript quantification tool Kallisto (V0.43.0) [77].

We used the edgeR package [78] to identify differentially expressed genes (FDR < 0.01 and |log_2_(fold change)| ≥ 1) between the 2 groups with either the 20% lowest or 20% highest EN300 values. Functional enrichment analysis of differentially expressed genes was performed using the Metascape tool [79]. Only Gene Ontology (GO) terms and KEGG pathways with a *P* value < 0.05 were considered significant and are listed.

The expression levels of 8 genes were verified using a quantitative PCR (q-PCR) approach. The β-actin gene of chicken was used as an endogenous control gene. Relative expression levels of objective mRNAs were calculated using the ∆∆Ct method. The primer sequences used for q-PCR are shown in Supplementary Table S10. All measurements contained a negative control (no complementary DNA template), and each RNA sample was analyzed in triplicate.

## Data Availability

The sequencing data for this project have been deposited in the NCBI and can be accessed with BioProject Nos. PRJNA730194 (Microbiome), PRJNA731001 (Whole genome resequencing), and PRJNA730355 (RNA-seq). Other data further supporting this work are openly available in the *GigaScience* repository, GigaDB [80].

## Additional Files

**Supplementary Figure S1**. The number distribution of OTUs with different existing ratio of samples. (**a**) The number plot of OTUs with different existing ratio of samples. (**b**) The relationship of existing ratio and the slope of the curve in **a**. The dotted line indicates the threshold utilized to remove existing ratio distribution trend due to great fluctuation.

**Supplementary Figure S2**. The α-diversity, relative abundance, and Spearman *r* values of specific microbiota among the 6 sites. (**a–e**) α-diversity comparison based on Good's coverage, observed OTU, ACE, Chao1, and Simpson indices, using Wilcoxon rank-sum test to determine significant differences. (**f**) Rarefaction curves of observed OTU. (**g**) α-diversity values of the 6 sites. Values are represented as median ± SD. (**h**) *P* values of Wilcoxon rank-sum test of each comparison for 6 α-diversity indices. (**i**) PCoA of the 768 samples based on unweighted UniFrac distances. (**j**) β-diversity comparison based on the weighted UniFrac distances among the 6 sites. The values are filled with weighted UniFrac distances (mean ± SD) in the corresponding comparisons. All comparisons were significantly different using Wilcoxon rank-sum test (*P* < 0.05). (**k**) Relative abundance of the top 10 dominant microbial phyla in the 6 sites. (**l**) Only microbial genera that were present in ≥461 samples (60% of the total) were plotted. Each row represents a microorganism. Among 2,475 Spearman *r* values, only 362 (14.62%) were significantly correlated (*P* < 0.05).

**Supplementary Figure S3**. Comparison of the functional capacities of the reproductive and digestive microbial communities among the 6 sites. (**a**) Overlap of the top 50 predictions among the 6 sites. (**b**) Heat map showing the 36 overlapped predictions with different abundances among the 6 sites. The heat map is color-coded based on row *Z* scores. (**c**) Map showing 65 site-associated bacterial taxa identified by LEfSe (LDA score >4) in the test trial.

**Supplementary Figure S4**. Significantly heritable microorganisms. The number of significantly heritable microorganism OTUs, genera, and species (*P* < 0.05) grouped by sampling phyla (**a, c, e**) and site (**b, d, f**).

**Supplementary Figure S5**. Significantly EN300-associated microorganisms. The number of microorganisms significantly associated with EN300 detected at OTU, genus, and species (*P* < 0.05) levels grouped by sampling phyla (**a, c, e**) and site (**b, d, f**).

**Supplementary Figure S6**. EN300-associated microorganisms. (**a**) The number of microbial genera (left) and OTUs (right) associated with EN300 at *P* < 0.05 of 3 test methods of 6 sites and their overlap. (**b, c**) Pearson and Spearman *r* values between EN300 and EN300-associated 26 genera and 39 OTUs. Red and blue tiles indicate positive and negative correlations, respectively. Significant *r* values are filled in numerically (*P* < 0.05).

**Supplementary Figure S7**. Pearson correlations between EN300 and EN300-associated microorganisms. (**a**) Pearson *r* values of candidate microbial species in the 6 sites. (**b**) Pearson *r* values among microbial species in each site. CP: crop; GZ: gizzard; IS: isthmus; SI: small intestine; UT: uterus; VA: vagina. Red and blue tiles indicate positive and negative correlations, respectively. The ratios on the right side of each site represent the number of significant correlations. *P < 0.05.

**Supplementary Figure S8**. Differences in the relative abundance of 5 species between the 20% of chickens with the highest and lowest egg production (EN300). (**a**) EN300 values for the 20% of individuals with the highest and lowest egg production. (**b**) EN300 values for the 20% of individuals with the highest and lowest abundances of *Clostridium leptum, Bacteroides salanitronis*, Firmicutes bacterium ZOR0006, *B. barnesiae*, and *B. fragilis* in the 3 reproductive tract sites. All comparisons were significantly different, established at *P* < 0.05.

**Supplementary Figure S9**. Top 20 functional categories enriched by 1,051 genes exhibited significant expression changes between groups with high and low egg production. The enrichment analysis was performed using the Metascape tool (See Methods). GO-BP: biological process (blue); GO-MF: molecular function (yellow); KEGG (red).

**Supplementary Figure S10**. Distribution of egg number at 300 days of age (EN300). Compared to the 20% of hens with the highest EN300 values, the 20% of hens with the lowest EN300 values exhibited a later start laying age, an earlier stop laying day, and irregular lay performance.

**Supplementary Figure S11**. Quality assessment of sequencing data. (**a**) Length distribution of reads. (**b**) Quality score of each base. (**c**) Quality score distribution of sequencing data. (**d**) Error rate distribution of reads.

**Supplementary Table S1**. Analysis of Bray-Curtis distance similarities.

**Supplementary Table S2**. Statistical test for the 65 functional capacities among the 6 sites.

**Supplementary Table S3**. Summary of host whole-genome sequencing.

**Supplementary Table S4**. Correlation between genetic relatedness matrix (GRM) and each Bray-Curtis (BC) distance or microbial relationship matrix (MRM) by Mantel test.

**Supplementary Table S5**. Heritability (*h^2^*) of the microbiota and cumulative abundance of heritable microbiota.

**Supplementary Table S6**. Heritability (*h^2^*) of reproductive traits from previous reports.

**Supplementary Table S7**. Summary of 16S rDNA sequencing.

**Supplementary Table S8**. Summary statistics of 16S rDNA sequencing.

**Supplementary Table S9**. Summary statistics of host whole-genome sequencing.

**Supplementary Table S10**. Primer sequences for q-PCR.

## Abbreviations

ANOSIM: analysis of similarity; BC: Bray-Curtis; bp: base pairs; BWA: Burrows-Wheeler Alignment tool; CPSA: capsular polysaccharide A; EN300: egg number at 300 days of age; FDR: false discovery rate; GATK: Genome Analysis Toolkit; GO: Gene Ontology; GRM: genetic relatedness matrix; GWAS: genome-wide association study; *h*^2^: heritability; kb: kilobase pairs; KEGG: Kyoto Encyclopedia of Genes and Genomes; LDA: linear discriminant analysis; LEfSe: linear discriminant analysis effect size; LMM: linear mixed model; *LYN*: lck/yes-related novel tyrosine kinase gene; M: million; *m*^2^: microbiability; MRM: microbial relationship matrix; NCBI: National Center for Biotechnology Information; OTU: operational taxonomic unit; PCoA: principal coordinates analysis; PCs: principal components; *PLAG1*: pleiomorphic adenoma gene 1; q-PCR: quantitative PCR; rDNA: recombinant DNA; RNA-seq: RNA sequencing; SNP: single-nucleotide polymorphism; Tb: terabase pairs; TPM: transcripts per million.

## Ethics Statement

All animal experiments were approved and reviewed by Animal Care and Use Committee Institutional of Sichuan Agricultural University (Approval No. DKY- 2,018,102,015).

## Competing Interests

The authors declare that they have no competing interests.

## Funding

This work was supported by the Sichuan Science and Technology Program (2019JDTD0009, 2020YFH0138, and 2021YFYZ0009) and the Fok Ying-Tong Education Foundation for Young Teachers in the Higher Education Institutions of China (161026).

## Authors’ Contributions

R.W., M.Y., D.Y., Y.L., B.Z, and M.Z., did bioinformatics analyses; D.L. wrote methods; D.L., M.L., and Q.Z. supervised the work.

## Supplementary Material

giab067_GIGA-D-21-00132_Original_Submission

giab067_GIGA-D-21-00132_Revision_1

giab067_Response_to_Reviewer_Comments_Original_Submission

giab067_Reviewer_1_Report_Original_Submission Erez Mills -- 6/14/2021 Reviewed

giab067_Reviewer_1_Report_Revision_1 Erez Mills -- 8/25/2021 Reviewed

giab067_Reviewer_2_Report_Original_Submission Ivan Rychlik -- 7/11/2021 Reviewed

giab067_Supplemental_Figures_and_Tables
