# Supplementary material for: Association of female reproductive tract microbiota with egg production in layer chickens
Source: Gigascience. 2021 Sep 23;10(9):giab067. doi: 10.1093/gigascience/giab067 (PMC8460357; doi:10.1093/gigascience/giab067)
Supplement: giab067_GIGA-D-21-00132_Original_Submission [file giab067_giga-d-21-00132_original_submission.pdf]

GigaScience

Female reproductive tract microbiota influence egg production in layer chickens

--Manuscript Draft--

**Manuscript Number:**	GIGA-D-21-00132
**Full Title:**	Female reproductive tract microbiota influence egg production in layer chickens
**Article Type:**	Research
**Funding Information:**	Department of Science and Technology of Sichuan Province (2019JDTD0009) — Pro. Diyan Li<br>Fok Ying Tong Education Foundation (161026) — Pro. Diyan Li<br>Department of Science and Technology of Sichuan Province (2020YFH0138) — Dr. Zhongxian Xu<br>Department of Science and Technology of Sichuan Province (2021YFYZ0009) — Pro. Mingzhou Li
**Abstract:**	The microbiota of the female reproductive tract is increasingly recognized as having fundamental roles in animal reproduction. To explore the relative contribution of reproductive tract microbiomes on egg production in chickens, we investigated the microbiota in multiple reproductive and digestive tract sites from 128 female layer (egg-producing) chickens in comparable environments. We identified substantial differences between the diversity, composition, and predicted function of site-associated microbiota. Reproductive tract microbiota were more profoundly affected egg production than that in the digestive tract. We identified four reproductive tract microbial species, Bacteroides fragilis , B. salanitronis , B. barnesiae, and Clostridium leptum , which were related to immune function and potentially contribute to enhanced egg production. These findings provide insights into the diverse microbiota characteristics of reproductive and digestive tracts, and may help design strategies for controlling and manipulating chicken reproductive tract microbiota to improve egg production.
**Corresponding Author:**	Diyan Li<br><br>CHINA
**Corresponding Author Secondary Information:**	
**Corresponding Author's Institution:**	
**Corresponding Author's Secondary Institution:**	
**First Author:**	Yuan Su
**First Author Secondary Information:**	
**Order of Authors:**	Yuan Su<br>Shilin Tian<br>Diyan Li<br>Wei Zhu<br>Tao Wang<br>Shailendra Kumar Mishra<br>Ranlei Wei<br>Zhongxian Xu<br>Mengnan He

Powered by Editorial Manager® and ProduXion Manager® from Aries Systems Corporation

	Xiaoling Zhao
	Huadong Yin
	Xiaolan Fan
	Bo Zeng
	Mingyao Yang
	Deying Yang
	Qingyong Ni
	Yan Li
	Mingwang Zhang
	Qing Zhu
	Mingzhou Li
**Order of Authors Secondary Information:**	
**Additional Information:**	
**Question**	**Response**
Are you submitting this manuscript to a special series or article collection?	No
**Experimental design and statistics**<br><br>Full details of the experimental design and statistical methods used should be given in the Methods section, as detailed in our Minimum Standards Reporting Checklist. Information essential to interpreting the data presented should be made available in the figure legends.<br><br>Have you included all the information requested in your manuscript?	Yes
**Resources**<br><br>A description of all resources used, including antibodies, cell lines, animals and software tools, with enough information to allow them to be uniquely identified, should be included in the Methods section. Authors are strongly encouraged to cite Research Resource Identifiers (RRIDs) for antibodies, model organisms and tools, where possible.<br><br>Have you included the information requested as detailed in our Minimum	Yes

Standards Reporting Checklist?	
**Availability of data and materials**<br><br>All datasets and code on which the conclusions of the paper rely must be either included in your submission or deposited in publicly available repositories (where available and ethically appropriate), referencing such data using a unique identifier in the references and in the “Availability of Data and Materials” section of your manuscript.<br><br>Have you have met the above requirement as detailed in our Minimum Standards Reporting Checklist?	Yes

Powered by Editorial Manager® and ProduXion Manager® from Aries Systems Corporation

Manuscript
Click here to access/download;Manuscript;Manuscript.docx

Female reproductive tract microbiota influence egg production in layer chickens

Yuan Su[1,†] , Shilin Tian[2,3,†], Diyan Li[1,*,†], Wei Zhu[1,†], Tao Wang[1,†], Shailendra Kumar Mishra[1], Ranlei Wei[4], Zhongxian Xu[1], Mengnan He[1], Xiaoling Zhao[1], Huadong Yin[1], Xiaolan Fan[1], Bo Zeng[1], Mingyao Yang[1], Deying Yang[1], Qingyong Ni[1], Yan Li[1], Mingwang Zhang[1], Qing Zhu[1,*], Mingzhou Li[1,*]

[1]Farm Animal Genetic Resources Exploration and Innovation Key Laboratory of Sichuan Province, Sichuan Agricultural University, Chengdu 611130, China.

[2]Department of Ecology, Hubei Key Laboratory of Cell Homeostasis, College of Life Sciences, Wuhan University, Wuhan 430072, China.

[3]Novogene Bioinformatics Institute, Beijing 100000, China.

[4]Center of Precision Medicine, West China Hospital, Sichuan University, Chengdu 610065, China.

[†]These authors contributed equally to this paper.

[*]Corresponding author. e-mail: Diyan Li: diyanli@sicau.edu.cn; Qing Zhu: zhuqing@sicau.edu.cn; Mingzhou Li: mingzhou.li@sicau.edu.cn

Abstract

The microbiota of the female reproductive tract is increasingly recognized as having fundamental roles in animal reproduction. To explore the relative contribution of reproductive tract microbiomes on egg production in chickens, we investigated the

microbiota in multiple reproductive and digestive tract sites from 128 female layer (egg-producing) chickens in comparable environments. We identified substantial differences between the diversity, composition, and predicted function of site-associated microbiota. Reproductive tract microbiota were more profoundly affected egg production than that in the digestive tract. We identified four reproductive tract microbial species, *Bacteroides fragilis*, *B. salanitronis*, *B. barnesiae,* and *Clostridium leptum*, which were related to immune function and potentially contribute to enhanced egg production. These findings provide insights into the diverse microbiota characteristics of reproductive and digestive tracts, and may help design strategies for controlling and manipulating chicken reproductive tract microbiota to improve egg production.

Keywords: microbiota, reproductive tract, egg production, chicken

Background

The domestic chicken (*Gallus gallus domesticus*) is of enormous agricultural significance, comprising broiler (meat-producing) and layer (egg-producing) chickens. Specialized commercial layer breeds were established during the twentieth century with greatly improved reproductive traits [1]. Currently, thousands of quantitative trait loci (QTLs) [2] and many gene mutations [3, 4] are reportedly associated with chicken reproductive traits. Nonetheless, egg production, as a polygenic inheritance trait, exhibits low to moderate heritability (h^2, ranging from 0.05 to 0.44, depending on the period involved) [5, 6]. Alternative effective approaches for modulating egg production in laying hens are urgently required for the poultry industry to meet consumer demand.

Distinct bacterial communities throughout the female reproductive tract form a

microbiota continuum from the vagina to the isthmus, which has a prominent role in animal reproduction [7, 8]. In humans, microbiome interactions with the host during pregnancy leading to preterm birth were investigated [9], and temporal changes in the vaginal microbiome associated with full-term pregnancies were identified [10]. An abnormal vaginal microbiota may predispose individuals to increased colonization of the genital tract, microbial invasion of the amniotic cavity, and fetal damage. The avian reproductive tract houses complex bacterial communities that are believed to play crucial roles in egg production [11]. Chicken digestive and reproductive tracts are mainly colonized by *Firmicutes*, *Bacteroidetes*, *Proteobacteria*, *Actinobacteria*, and *Fusobacteria*, which are spatially organized within specific digestive and reproductive compartments [12, 13]. Additionally, *Lactobacillus* species were found to be keystone species residing in the chicken oviduct [14].

Several synergistic factors, such as environment and diet, dominate over host genetics in determining gut microbiota composition [15, 16]. A comparative study of gut microbial diversity among parrot species indicated the potential role of host ancestry in shaping the gut microbiome [17]. A genome-wide association study (GWAS) in chickens demonstrated the genetic loci rs15142709 and rs15142674 which are located in the pleiomorphic adenoma gene 1 (*PLAG1*) and lck/yes-related novel tyrosine kinase (*LYN*) genes were significantly associated with microbial *Methanobacterium* abundance [18]. In a previous study, 14 identified QTLs strongly influenced *Clostridium leptum* and *Lactobacillus* abundance, as well as related candidate genes involved in anti-inflammatory responses and the motility of the digestive tract [19]. On the other hand, recent studies have suggested that host genetics have limited impact on gut microbiota composition in humans [20]. Therefore, we realized that the relative abundance of some microorganisms is influenced by host

genetics.

We speculated that the microbial component of the reproductive tract might be an important aspect of egg production in chicken. Here, we performed whole-genome sequencing on 128 laying hens and profiled taxonomic abundance in 768 samples from three reproductive (vagina, uterus, and isthmus) and three digestive (crop, gizzard, and small intestine) tract sites by sequencing the V4 region of the 16S rRNA gene. We characterized the reproductive tract microbiota and its features compared with those of the digestive tract microbiota of hens. We identified the contribution of key microorganisms to egg production, and established a correlation between host genetics and the microbial diversity of six tract sites. These findings provide insights into the microbial communities in the reproductive tract of highly specialized layer populations, which may help develop strategies to enhance commercial egg production.

Methods

Chickens

The study was conducted on a common flock of 128 Dongxiang green shell laying hens reared on an experimental poultry farm at Sichuan Agricultural University in Ya'an, Sichuan, China. All chicks were hatched on the same day and housed in individual pens. Feed intake was controlled daily according to standard farm husbandry practices and water was provided *ad libitum*. The number of eggs produced for the first 300 days of life was recorded daily for each individual. We determined that the mean number of eggs (~75.32; ranging from 24-129) that each hen laid by 300 days of age (EN300) fit a normal distribution pattern (P = 0.725, Kolmogorov-Smirnov test) (**Supplementary Fig. S1**). At the age of 300 days, 2 mL of whole blood was collected from the wing

vein using venipuncture and stored at -20 °C. Subsequently, each individual was culled by cervical dislocation followed by decapitation. After laparotomy, fresh tissue was collected from three sites in the reproductive tract (vagina, uterus and isthmus) and three sites in the digestive tract (crop, gizzard and small intestine) (**Fig. 1a**). Samples were snap-frozen in liquid nitrogen, transported to the laboratory, and stored at -80 °C until further analysis.

Microbial genomic DNA extraction

Total microbial genomic DNA from lumen of digestive tracts and mucus of reproductive tracts were extracted from ~200 mg tissues using a TIANamp Stool DNA Kit (Tiangen Biotech, Beijing, China) according to the manufacturer's instructions. Host DNA was isolated from blood using a TIANamp Genomic DNA Kit (Tiangen Biotech) following the manufacturer's instructions. The extracted DNA was quantified using a NanoDrop2000 spectrophotometer (Thermo Fisher Scientific, Chengdu, China), and DNA integrity was determined by 1% agarose gel electrophoresis.

16S rRNA amplicon and sequencing

The V4 hypervariable region of the bacterial 16S rRNA gene was amplified using a HOTSTAR Taq Plus Master Mix Kit (Qiagen, Shanghai, China) and the universal primers 515 F (5′-GTGCCAGCMGCCGCGGTAA-3′) and 806 R (5′-GGACTACHVGGGTWTCTAAT -3′) [21]. Reactions were carried out using 15 μL of Phusion High-Fidelity PCR Master Mix (New England Biolabs, Ipswich, Ma, USA), 3 μL of the forward and reverse primers, 10 μL of template DNA, and 2 μL of ddH_2O, no template control was also performed. The PCR cycling conditions were as follows:

an initial denaturation step at 98 °C for 1 min and 30 cycles of 98 °C for 10 sec, 50 °C for 30 sec, and 72 °C for 30 sec, followed by an extension step at 72 °C for 5 min, using a Bio-Rad CFX96 thermal cycler (Bio-Rad Laboratories, Hercules, CA, USA). Amplicons were purified on agarose gel (1%) using a GeneJET Gel Extraction kit (Thermo Fisher Scientific, Schwerte, Germany). A DNA library was prepared using an Ion Plus Fragment Library Kit (Thermo Fisher Scientific) based on the manufacturer's instructions. Reads were barcoded per sample, combined for multiplexed sequencing with the Ion S5^{TM} XL platform (Thermo Fisher Scientific) to generate 400-bp single-end reads, and sequenced by Novogene Bioinformatics Technology Co. Ltd of China.

Whole-genome sequencing

After qualified host DNA samples were tested, the DNA was randomly fragmented using an ultrasonicator (Covaris Inc., Woburn, MA, USA), and then a sequencing library was prepared using a TruSeq Nano DNA HT Sample Preparation Kit (Illumina, San Diego, CA, USA) following the manufacturer’s instructions. Index codes were added to tag each sample. DNA fragments were then end-repaired, dA-tailed, and ligated with a full-length adapter for Illumina sequencing, PCR amplification, and purification. Next, isolated DNA libraries were constructed with an insert size of 350 bp. Finally, genomes of the 128 individuals were separately sequenced with 150 bp paired-end reads using the Illumina Novaseq platform by Novogene Bioinformatics Technology Co. Ltd.

16S rRNA-seq data processing

The raw data obtained by sequencing were first filtered to obtain high-quality data. First,

the adapter sequences in 61.05 million (M) raw reads (**Supplementary Table S1**) were trimmed using Cutadapt (version 1.9.1) [22] when the overlap length between the read and the adapter was shorter than 10 bp. Then, barcode sequences were trimmed and reads that were too long (> 260 bp) or too short (< 220 bp) were filtered using Cutadapt with the parameters "-e 0 -q 17 -m 200 -M 2600". Next, the remaining reads were compared with the ChimeraSlayer reference database using the UCHIME algorithm [23] to detect and then remove chimera sequences. Finally, low-quality reads (i.e., more than 50% of bases with a phred quality lower than five) were also removed. Consequently, 57.61 M high-quality reads were generated for subsequent analysis (**Supplementary Table S2**). Greater than 93.85% of the high-quality reads had lengths of 250 - 260 nt. Data with a quality score > 20 accounted for 88.14% of all the effective bases. The error ratio of the sequencing reads was relatively high in the ending position.

Operational taxonomic unit (OTU) cluster and species annotation

The remaining high-quality sequences were used to generate operational taxonomic units (OTUs) by Uparse software (version 7.0.1001) [24] with an identity cutoff of 97%. Singleton OTUs that did not match the reference database were removed. Clustering across all samples from the 128 chickens produced 46,480 OTUs after singleton removal. Nonetheless, most of those OTUs were present in low abundance and were found in very few samples. We then discarded OTUs that were not found in at least 20% of the chickens in each sampling site, yielding 6,776 OTUs. For each OTU, the SSUrRNA library in the Silva (https://www.arb-silva.de/) [25] was used to annotate taxonomic information (i.e., kingdom, phylum, class, order, family, genus and species) based on the Mothur algorithm [26]. Subsequently, we determined the phylogenetic

relationship of different OTUs and dominant species differences in samples (groups) after multiple sequence alignment using MUSCLE software (version 3.8.31) [27]. Additionally, OTU abundance information was normalized using a standard sequence number corresponding to the sample with the fewest sequences.

Alpha diversity

Alpha diversity was used to analyze the complexity of species diversity for a sample based on normalized OTUs through six indices (i.e., observed OTUs, Chao1, Shannon, Simpson, ACE, Good's coverage), using the QIIME2 software [28]. Among these, Chao1 and ACE were selected to identify community richness, Shannon and Simpson were used to identify community diversity, and Good's coverage was used to characterize sequencing depth. Differences in alpha diversity indices among the six sites were calculated with the Wilcoxon rank-sum test using R software (version 2.15.3).

Beta diversity

Beta diversity was used to evaluate differences in samples. Beta diversity in Bray-Curtis (BC) and weighted/unweighted UniFrac distances were calculated using QIIME2 software [28]. The BC ordination provided position values along an ordination axis and distances from the axis for samples of communities.

Principal coordinates analysis (PCoA)

PCoA was performed to obtain principal coordinates and to visualize complex, multidimensional data. A distance matrix of previously obtained weighted/unweighted

UniFrac distances among samples was transformed to a new set of orthogonal axes, by which the maximum variation factor was demonstrated by the first principal coordinate, the second maximum variation factor was demonstrated by the second principal coordinate, and so on. PCoA was performed using the WGCNA package [29], stat packages, and ggplot2 package in R software.

Prediction of the functional profiles of microbial communities

The functions of the microorganisms present in the microbial communities detected in the six sites were predicted using PICRUSt2 [30]. We used the Wilcoxon rank-sum test to investigate differences in pathways among sites. *P*-values were adjusted using the Benjamini-Hochberg method by the false discovery rate (FDR) with the p.adjust function in R.

Community difference analysis

Pairwise comparisons between different sites were statistically compared using Analysis of Similarity (ANOSIM, also named permutational MANOVA) with 10,000 permutations based on BC ordination to evaluate the reasonability of the division of groups.

Between-group variation analysis

High-dimensional biomarkers were discovered by Linear discriminant analysis (LDA) Effect Size (LEfSe) using the parameter ‘LDA score > 4’ [31] to identify characteristics of abundance and related classes (e.g., genes, metabolites, or taxa).

Identification of microbiota constituents related to egg production

EN300 values between two groups (the lowest- and highest-ranked 20% of chickens with respect to their EN300 value) were then compared using the Wilcoxon rank-sum test. Microorganisms with $P < 0.05$ and FDR < 0.05 were retained. Furthermore, we calculated the Spearman's r and Pearson's r between EN300 and the abundance of each microbiota constituent at genus, OTU, and species levels. A significant correlation between the presence of a microorganism and the EN300 value was considered if $P < 0.05$, as determined using the psych package in R with the P value adjusted using the Benjamini-Hochberg method. Overlapping microorganisms obtained from the Wilcoxon rank-sum test and Spearman's r and Pearson's r were considered to have a potential relationship with EN300. We subsequently characterized EN300-associated microbes in the six sites.

Whole-genome sequencing data processing

To avoid analysis noise caused by sequencing errors, low-quality paired reads (reads with ≥ 10% unidentified nucleotides [N]; > 10 nt aligned to the adaptor, allowing ≤ 10% mismatches; > 50% bases having phred quality < 5; and putative PCR duplicates generated in the library construction process), which mainly resulted from base-calling duplicates and adaptor contamination, were removed using an in-house script [32]. Consequently, 1.30 terabases (~10.15-fold per individual) of high-quality paired-end reads were obtained, including 95.13% and 88.98% nucleotides with phred quality ≥ Q20 (with an accuracy of 99.00%) and ≥Q30 (with an accuracy of 99.90%), respectively (**Supplementary Table S3**).

Read mapping, and genomic variant calling and annotation

The remaining high-quality reads of each individual were aligned to the reference chicken genome (Gallus_gallus-6.0 Ensembl release 94, http://asia.ensembl.org/Gallus_gallus/Info/Index/) using the Burrows-Wheeler Alignment tool (BWA) (version 0.7.15) [33] with the command ‘mem -t 10 -k 32’. BAM alignment files were then generated using SAMtools (version 0.1.19) [33]. Additionally, we improved alignment performance through filtering the alignment reads with mismatches ≤ 5 and mapping quality = 0. After sorting by SAMtools, the sorted BAM file was marked in duplicate using the command “MarkDuplicates” in the package Picard (version 1.119).

Subsequently, we performed gVCF calling in accordance with the Genome Analysis Toolkit (GATK) best practices pipeline (version v3.7) [34] using the HaplotypeCaller-based method, and then population single nucleotide polymorphism (SNP) calling by merging all gVCFs with the commands “CombineGVCFs”.

To obtain high-credibility SNPs, we applied the hard filter command ‘VariantFiltration’ to exclude potential false-positive variant calls as follows: (a) quality by depth >10.0; (b) mapping quality score > 40.0; (c) FS < 60.0; (d) MQRank-Sum > −12.5; (e) ReadPosRankSum > −8.0. In addition, the filter variants were further filtered when more than three SNPs clustered within a 10-bp window were removed [35]. Finally, we used vcftools (version 0.1.15) to obtain biallelic variants with the following parameters: sample call rate > 90%, SNP call ratio > 95%, minor allele frequencies > 1%, and Hardy-Weinberg equilibrium P value < 10^{-5}. Ultimately, a total of 10.82 M high-credibility SNPs in 128 individuals were retained (**Supplementary Table S4**).

SNPs were classified into different genomic regions (i.e., exonic, intronic, splice sites, upstream and downstream around gene regions, and intergenic) using the ANNOVAR package [36].

Construction of microbial relationship and host genetic relatedness matrices

OTUs identified in each site were normalized to a zero mean and unit variance. We then constructed a microbial relationship matrix (MRM) [37] using an R script based on the following equation:

$$r_{tij} = \frac{1}{N_T}\sum_{o=1}^{N_T} \frac{(a_{tio} - \overline{t_{to}})(a_{tjo} - \overline{a_{to}})}{\sigma_{to}^2}$$

where r_{tij} represents the tested microbial relationship in tract t between chickens i and j; a_{tio} and a_{tjo} are the abundance of OTU o in tract t in chickens i and j, respectively; $\overline{t_{to}}$ is the average relative abundance of OTU o in tract t in the population; σ_{to}^2 is the variance in the abundance of OTU o in tract t; and N_T is the total number of OTUs in tract t used for the computation of relatedness. High-quality SNPs were further used to detect independent markers using PLINK [38], with the following parameters: 50 kb window size, 10 SNPs per step, and 0.2 as a squared Pearson's r (r^2). All 10,809,968 SNPs were used to compute the principal components (PCs) and genetic relatedness matrix (GRM) [39] using GCTA version 1.91.1 [40]:

$$h_{ij} = \frac{1}{N}\sum_{a=1}^{N} \frac{(r_{ia} - 2\overline{f_a})(r_{ja} - 2\overline{f_a})}{2\overline{f_a}(1 - \overline{f_a})}$$

where h_{ij} is the tested genetic relationship between chickens i and j; r_{ia} and r_{ja} represent the number of reference alleles in chickens i and j, respectively; $\overline{f_a}$ is the

frequency of the reference allele in the population; and N is the number of variants.

Heritability (h^2) analysis

To estimate the effects of host genetics on the microbiota at different sites, we computed the correlation between GRM and BC distances at each site using both Pearson's r and Spearman's *r*, based on Mantel tests with 10,000 permutations. The correlation between GRM and MRM was also computed. To estimate the correlation between GRM and the microbiota community, we computed heritability at OTU, genus, and species levels. OTU abundance information was normalized using a standard sequence number corresponding to the sample with the least number of sequences. Microorganisms that were present in < 60% but ≥ 20% of the samples were dichotomized as present or absent [41], and the microorganisms that were detected in < 20% of the samples from each site were excluded from the analysis.

Genetic and microbial parameters of egg production

As the individuals examined in this study had no pedigree information, we computed the SNP-based heritability of the egg production phenotype (i.e., EN300) instead, using the following model [39]:

$$y = K_c + g + e[A]$$

where y is an observed value (EN300); c is a vector of fixed covariates with the corresponding design matrix K; e is the residual effect; and g is a vector of aggregate effects of all SNPs with an ~N(0,$G_{\sigma_A^2}$), where G and σ_A^2 are the GRM and polygenetic variance (overall SNP effects), respectively. The top five host genetic PCs were

considered covariates in the model to account for the calculated population stratification, as described above. The likelihood ratio test P value was calculated to examine the significance of the association between SNPs and EN300.

The fraction of EN300 variance explained by microbial variance was calculated as $m^2 = \frac{\sigma_m^2}{\sigma_p^2}$; (called 'microbiability' [m^2] in animals [37] and 'microbiome-association index' in humans [16]), where σ_m^2 and σ_p^2 are the phenotypic variance and microbial variance, respectively. To adjust for host genetic effects, all valid individuals and SNPs were used in a GWAS with a univariate linear mixed model (LMM), which was performed using GEMMA [42]. The LMM was calculated as follows:

$$y=K_c+m_s + e \text{ [B]}$$

where the model parameters are the same as those described in model [A], except m_s, which is the random effect of the microbiota in locations following the multinomial distribution m_s~N(0,Mσ_m^2), and M is the MRM. We then used the MRM in GCTA to calculate m^2. The genome-wide significance threshold was 10^{-6}. We then extracted these SNPs with significant effects on EN300 and calculated the PCs using PLINK. The first two PCs and the top five host genetic PCs were then used as covariates in model [B] to account for host genetics.

RNA sequencing (RNA-seq) analysis

For RNA-seq, total RNA was extracted from uterine tissue of twelve hens (six for each of the groups with either the 20% lowest or 20% highest EN300 values) using the RNeasy Mini Kit (Qiagen). We used an rRNA depletion protocol (Ribo-Zero kit,

Epicenter) coupled with the Illumina TruSeq stranded RNA-seq library protocol to construct the RNA-seq libraries. A total of 12 libraries were quantified using the Qubit dsDNA High Sensitivity Assay Kit (Invitrogen) and separately sequenced on the NovaSeq 6000 platform (Illumina) to produce an average of ~31.86 M 150-bp paired-end raw reads and ~30.52 M high-quality reads for each library. Sequence reads were aligned to the chicken reference genome (Gallus_gallus-6.0 Ensembl release 94) by the STAR alignment tool (version 2.5.3a). On average, ~96% of reads of individual libraries were aligned to the chicken reference genome, generating an average of 29.30 M aligned reads for each sample. The gene expression level was then estimated as transcripts per million (TPM) using the high-speed transcript quantification tool Kallisto (V0.43.0) [43].

We used the edgeR package [44] to identify differentially expressed genes (FDR < 0.01 and $|\log2(\text{fold change})| \geq 1$) between the two groups with either the 20% lowest or 20% highest EN300 values. Functional enrichment analysis of differentially expressed genes was performed using the Metascape tool [45]. Only Gene Ontology (GO) terms and Kyoto Encyclopedia of Genes and Genomes (KEGG) pathways with a P value < 0.05 were considered significant and are listed.

The expression levels of eight genes were detected using a quantitative PCR (q-PCR) approach. The β-actin gene of chicken was used as an endogenous control gene. Relative expression levels of objective mRNAs were calculated using the $\Delta\Delta$Ct method. The primer sequences used for q-PCR are shown in **Supplementary Table S5**. All measurements contained a negative control (no cDNA template), and each RNA sample

was analyzed in triplicate.

Results and Discussion

Discriminative characteristics of microbiota in reproductive and digestive tract sites

Sequencing of the 16S rRNA gene in 768 samples generated a total of ~57.61 M high-quality reads (~75.01 K reads per sample). *De novo* clustering after singleton removal produced 46,480 OTUs at an identity cutoff of 97%, among which 6,776 OTUs found in > 20% of samples were used for subsequent analysis. We performed alpha diversity analysis based on qualified sequencing depth with an average Good's coverage of 98.69% (ranging from 96.30-99.60%) (**Supplementary Fig. S2a**). Analysis of five indices (i.e., observed OTUs, Chao1, Shannon, Simpson, and ACE) (**Supplementary Fig. S2b-g**) indicated that the vast majority of pairwise comparisons between sites (10 of 90 pairwise comparisons, or 11.11%) showed significant differences ($P < 0.001$, Wilcoxon rank-sum test), with the exception of two comparisons (uterus vs. isthmus for all indices; and small intestine vs. vagina for the observed OTUs, Shannon, and Simpson indices) (**Supplementary Fig. S2h, i**).

Compared to the digestive system, the reproductive system exhibited higher alpha diversity (all five indices) and thus contained more microbial taxa, especially in the upper reproductive tract (i.e., uterus and isthmus) (**Fig. 1a, b**). Similar to significant microbiota differences between the vaginal and upper reproductive tracts in humans [7, 46], we found highly discriminative microbial communities in chickens between the upper (isthmus and uterus) and lower (vagina) reproductive tracts (isthmus vs. vagina, $R = 0.473$, $P < 0.001$, ANOSIM; uterus vs. vagina, $R = 0.496$, $P < 0.001$, ANOSIM),

but indistinguishable microbiota between the isthmus and uterus (isthmus vs. uterus, *R* = -0.003, *P* = 0.694, ANOSIM) (**Supplementary Table S6**). These results demonstrated microbiota heterogeneity throughout contiguous sites of the digestive and reproductive tracts in hens.

We used PCoA to visualize differences in taxa composition between microbiota in the reproductive and digestive tracts. The first principal component, explaining 34.96% of the variance in weighted UniFrac distance matrices among the samples, separated reproductive and digestive tract samples (**Fig. 1c**). Given that reproductive and digestive tracts share a common exit in the cloaca, the frequently exchanged microbiome likely resulted in similar microbiota at the distal end of both tracts. Consequently, we found that the vagina acquired microbe communities from the isthmus and uterus, which all belong to the reproductive tract. Nonetheless, the vagina microbiota was partially indistinguishable from that of the small intestine (**Fig. 1c**). The unweighted UniFrac distance matrices (**Supplementary Fig. S2j**) and an ANOSIM based on BC distances **(Supplementary Table S6**) recapitulated these findings.

Similar phyla dominated the microbiota in the six sites; *Firmicutes*, *Proteobacteria*, and *Cyanobacteria* accounted for 71.45% - 97.86% of all OTUs. Nonetheless, some differences were observed among the sites. *Cyanobacteria* was the dominant phylum in the gizzard (48.19% of the total abundance); however, *Firmicutes* was the most abundant phylum (43.60% to 78.93%) in the other five sites. We also found that the uterus and isthmus had similar dominant phyla, including *Firmicutes* (44.87% and 43.60%), *Proteobacteria* (26.25% and 23.77%), and *Bacteroidetes* (17.13% and 19.52%) (**Supplementary Fig. S2k**). Strikingly, the vagina had the highest abundance of *Fusobacteria* (11.51%) among the six sites.

At the genus level, *Lactobacillus* (7.24% - 73.74% in the six sites), *Exiguobacterium* (2.79% - 4.68%), *Stenotrophomonas* (1.01% - 6.54%), and *Bacteroides* (3.40% - 4.86%) were ubiquitously found across all sites with higher abundances than other bacteria due to their broad adaptability and beneficial functions (**Fig. 1a**). Supporting the well-documented protective role of *Lactobacillus* by lowering the environmental pH through lactic acid production, we found *Lactobacillus* to be more dominant in the digestive tract (73.74% in crop, 24.76% in gizzard, 30.70% in small intestine) compared with the reproductive tract (7.24% in vagina, 9.27% in uterus, 9.91% in isthmus). The digestive tract had a lower pH than the reproductive tract where an alkaline pH is needed to maintain sperm motility [47, 48]. *Unidentified Erysipelotrichaceae, Unidentified Chloroplast, Lactobacillus,* and *Bacteroides* had abundances of >1.0% in the vagina, which was further increased in the uterus and isthmus (**Fig. 1a**).

Furthermore, 14.63% of genera (362 of 2,475) demonstrated associations between sites ($P < 0.05$ of Spearman' s *r,* Z-test) (**Supplementary Fig. S2l**). Typically, genera belonging to *Proteobacteria* and *Firmicutes* showed significantly positive correlations ($P < 0.001$, *Z*-test) between the crop and gizzard, the gizzard and small intestine, or the three reproductive tract sites.

Site-associated microorganisms in reproductive and digestive tracts

We analyzed the functional capacity of the microbiota in each reproductive and digestive tract site using PICRUSt2, and found that 72.00% of the representative pathways (36 of the top 50 KEGG pathways) were shared across the six sites, one third of which (12 of 36) were primarily involved in metabolism (**Supplementary Fig. S3a, b**). Specifically, 'bacterial secretion system' and 'bacterial chemotaxis' were enriched

in the reproductive tract. A previous study found that successful bacterial pathogens evolved versatile protein secretion systems to promote their survival and fitness in response to different environmental challenges, and to modulate host immunity [49]. Seven pathways were specifically enriched at a site (three of six were site-specific to crop and gizzard, and 'riboflavin metabolism' was specific to vagina). Abundances of the OTUs involved in these pathways differed among the six sites ($P < 0.001$, Wilcoxon rank-sum test) (**Supplementary Table S7**). For example, the microbial community of the small intestine had important roles in 'valine, leucine, and isoleucine biosynthesis', as indicated by the moderate row Z scores (-0.66) for each pathway. Moreover, 'propanoate metabolism' (Z score = 1.72) and 'bacterial chemotaxis' (Z score = 1.53) were overrepresented in the vagina. Meanwhile, 'bacterial secretion system' was overrepresented only in the uterus (Z score = 1.29) and isthmus (Z score = 0.94) compared with the vagina and the three digestive tract sites (**Fig. 2a**).

We next identified 65 site-associated bacterial taxa among the six sites using LEfSe; [31] (**Fig. 2b and Supplementary Fig. S3c**), which confirmed most of the observations described above (i.e., the uterus and isthmus showed essentially similar microbiota). Of note, *Helicobacter* and *Unidentified Erysipelotrichaceae*, which were associated with the small intestine, showed the highest abundance among the six sites (**Fig. 2b**). Six genera from *Lactobacillaceae* were crop-associated bacteria. In chicken, the crop acts as a reservoir for the storage of food prior to its digestion, where food mixes with many beneficial *Lactobacillus* bacteria (73.84% at the genus level) that produce lactic acid before moving on to the proventriculus [50]. Next, the gizzard grinds any remaining large food particles with the assistance of grit, releasing abundant *Unidentified Chloroplast* (2.94%) and mitochondria-like (2.37%) microbes from plant consumption. The small intestine exhibited the most abundant microbes of the three digestive tract

sites, which is mainly where further digestion occurs, and fermentation begins. *Paenibacillaceae* species, with optimum growth at pH 6.0-7.0, were also overrepresented. *Helicobacter* specifically inhabits the small intestine in chicken to maintain near neutral pH and a microaerophilic environment, and may be involved in inflammation, metabolism, and neutralization of gastric acid [51]; whether this is beneficial or harmful remains largely unknown.

Unidentified Erysipelotrichaceae showed higher abundance in the three reproductive tract sites (1.83% - 2.40%). Bacteria associated with the isthmus and uterus both showed higher abundance than in the other sites. Several genera (typically, *Romboutsia*, *Fusobacterium,* and *Clostridium sensu stricto 1*) were dominant in the vagina (> 25% of the microbiota) but had lower abundance in the other sites (**Fig. 2a**). Among these, *Romboutsia* has been negatively associated with body weight, fasting serum glucose, and insulin in mice [52]. *Fusobacterium* is considered a proinflammatory organism in humans [53]. *Clostridium* is reportedly capable of utilizing a broad range of organic substrates for efficient hydrogen production [54], thus adjusting the pH of the host. *Gammaproteobacteria* and *Betaproteobacteria* were uterus-associated (LDA > 4); the abundances of these two bacterial taxa changed in immune-suppressed honey bees emerging from Varroa-infested colonies [55]. Six Bacteroidetes bacterial taxa were isthmus-associated; *Bacteroides* species are thought to play a vital role in the breakdown of polysaccharides into simpler compounds related to host immunity.

Taken together, we speculate that the reproductive tract microbiota plays a vital role in improving protective immunity, defending against invasion of the reproductive tract by harmful bacteria, and pH adaptation. These findings confirm that the digestive and reproductive tract microbiota in chicken are primarily determined by the physiological function of each compartment within these systems.

Weak association between host genetics and microbial communities

In order to explore the relationship between host genome and microbiome of six sites, we generated a total of 1.76 tera bases of high-quality genome sequences from 128 chickens with ~10.15-fold average depth for each individual (**Supplementary Table S3**), and identified a total of 10.82 M SNPs with a density of ~10.29 SNPs per kb.

The correlation between host genetics (using GRM) and microbial beta diversity based on BC distance at the six sites in the same cohort of laying hens was not statistically significant ($|r| < 0.033$, $P > 0.05$, Mantel test, **Fig. 3a-f and Supplementary Table S8**). Nonetheless, the microbiomes of anatomically neighboring sites were similar. Typically, the microbial communities of the isthmus were positively correlated with those of the neighboring uterus (Spearman's $r = 0.426$, $P < 0.0001$, Mantel test), but not significantly associated with the relatively distant crop (Spearman's $r = 0.019$, $P = 0.335$, Mantel test, **Supplementary Table S8**). We also estimated the association between GRM and MRM, and obtained similar results: both Pearson's and Spearman's correlations suggesting that host genetics and the microbiota composition are weakly associated (**Supplementary Table S8**).

We next regarded the abundance of each microorganism as a quantitative trait to estimate the h^2 of each microorganism at the species, genus, and OTU level. Microorganisms in > 20% but < 60% of samples (**Supplementary Fig. S4a, b**) were analyzed qualitatively as dichotomous traits (**Fig. 3g-i**). At the species and genus levels, no significant correlation ($P > 0.05$, Wilcoxon rank-sum test) was found between the presence of a SNP and the presence of a specific microbe (**Fig. 3j-l**) in the crop. Three species in the small intestine (accounting for 0.21% of the tested small intestine

microbiota), six in the vagina (0.39%), 14 in the uterus (0.92%), and ten in the isthmus (0.66%) exhibited significant SNP-based heritability ($P < 0.05$, Wilcoxon rank-sum test) (**Supplementary Table S9**). Most of these heritable bacteria belonged to the *Firmicutes* phylum (**Supplementary Fig. S4c, e, g)**. Reproductive tract sites had more heritable bacterial phyla than digestive tract sites (**Supplementary Fig. S4d, f, h**). The cumulative abundances of these heritable bacteria were only 0.22%, 4.14%, 1.46%, and 1.61% ($P < 0.05$, Wilcoxon rank-sum test) in the small intestine, vagina, uterus, and isthmus, respectively (**Fig. 3n and Supplementary Table S9**). Similar results were observed at the genus and OTU levels (**Fig. 3m, o**). These results supported that host genetics have limited effect on shaping the microbial composition of the reproductive and digestive tracts.

Heritability (h^2) and microbiability (m^2) of EN300

To further explore the effect of genome and microbiome on EN300, we used a GRM of sample pairs to estimate the h^2 value of EN300 explained by whole genome SNPs using the restricted maximum likelihood method. We found that EN300 exhibited relatively low to medium heritability ($h^2 = 0.282$, $P = 0.048$, likelihood ratio test), which was comparable to previous estimations (**Supplementary Table S10**) [5, 6]. The fraction of EN300 variance explained by microbial variance was measured by microbiability (m^2) [16]. After correcting for host genetic factors using EN300-related SNPs as additional covariates, we found that the estimated EN300 m^2 values for digestive tract sites (0.523 for small intestine, 0.869 for crop, and 0.873 for gizzard) were lower than those for reproductive tract sites (0.923 for vagina, 0.936 for uterus, and 0.989 for isthmus) (**Table 1**). Generally, higher EN300 m^2 values were observed

for sites neighboring the ovaries; the isthmus was the most pertinent site with respect to egg production. Commercial egg producers are acutely interested in hen oviducts because pathological changes or disrupted activity directly affect egg production efficiency and ultimately decrease economic profitability [56]. In chickens, the inner and outer shell membranes form in the isthmus, while calcification of the eggshell, subsequent pigmentation, and cuticle deposition occur in the uterus and is followed by expulsion of the egg through the vagina [57]. These results suggest that EN300 in layer chickens is determined more by the microbiota in the reproductive tract than in the digestive tract.

Table 1 Estimated microbiability (m^2) of EN300

	Site	m^2	Standard error	*P* value
**Digestive tract**	Crop	0.869	0.049	$< 10^{-16}$
	Gizzard	0.873	0.045	$< 10^{-16}$
	Small intestine	0.523	0.111	2.56×10^{-11}
**Reproductive tract**	Isthmus	0.989	0.011	$< 10^{-16}$
	Uterus	0.936	0.030	$< 10^{-16}$
	Vagina	0.923	0.028	$< 10^{-16}$
**Host genetics**		0.282	0.231	0.049

Microorganisms in the reproductive tract are significantly associated with EN300

We next focused on the microorganisms which are highly associated with EN300. The results showed that most of the microorganisms detected at the microbial species, genus, and OTU levels that significantly associated with EN300 belonged to the *Firmicutes* phylum ($P < 0.05$, Wilcoxon rank-sum test) (**Supplementary Fig. S5**). Only microorganisms that exhibited a significant correlation between egg production and

relative abundance as determined by both Pearson's *r* and Spearman's *r* were considered a causal relationship ($P < 0.05$, Wilcoxon rank-sum test) Consequently, 39 OTUs, 26 genera, and 24 species fulfilled these criteria (**Fig. 4a and Supplementary Fig. S6a**).

Most OTUs, genera, and species present in the three digestive tract sites were negatively correlated (Pearson's *r*) with egg production (negative/positive: 19/6, 8/8 and 16/5, respectively), whereas they were positively correlated with egg production in the reproductive tract sites (positive/negative: 13/9, 11/10 and 13/4, respectively) (**Fig. 4b and Supplementary Fig. S6b, c**). Microorganisms in the uterus were most strongly correlated with each other (**Fig. 4c and Supplementary Fig. S7**), which implied a strong symbiotic/competitive relationship.

At the genus level, *Lactobacillus*, *Bacteroides,* and *Desulfovibrio* were positively correlated with EN300 in the three reproductive tract sites. *Pseudomonas, Exiguobacterium,* and *Unidentified Erysipelotrichaceae* were negatively correlated with EN300 in the three reproductive tract sites, but were positively correlated in the three digestive tract sites (**Supplementary Fig. S6b**).

At the species level, *Bacteroides fragilis*, *B. salanitronis*, *B. barnesiae,* and *C. leptum* were positively correlated with EN300 in the three reproductive tract sites, but were weakly negatively correlated with EN300 in the small intestine, while no correlation was found in the crop and gizzard. The first three species belong to the genus *Bacteroides*, which had a significantly positive correlation with egg production in the three reproductive sites (Pearson's r = 0.403 - 0.479). *Bacteroides* species have been identified as the predominant anaerobic genera in chicken cecum [58], which were thought to play an important role in the breakdown of polysaccharides into simpler

compounds used by the animal host as well as the microorganisms themselves [59]. Intestinal anaerobic bacteria such as *B. fragilis* and *B. salanitronis* have been suggested to possess metabolic pathways for N-glycan production [60]. The symbiont *B. fragilis* exists in a commensal relationship with the host as it expresses a relatively large number of genes involved in polysaccharide metabolism, which benefits the host. The surface of *B. fragilis* can produce polysaccharides; in particular, capsular polysaccharide A (CPSA) is a key mediator of mammalian immune system development [61]. Surprisingly, CPSA has also been shown to exert protective effects in autoimmune disorder models, such as antibiotic-induced experimental encephalomyelitis. It is thus suggested that the genus *Bacteroides* could regulate reproductive activity by mediating the avian immune system.

Firmicutes bacterium ZOR0006 had a significantly negative correlation with EN300 in the three reproductive tract sites and a significantly positive correlation in the three digestive tract sites. The 20% of chickens with the lowest EN300 values (mean = 37.13) had significantly lower *B. fragilis*, *B. salanitronis*, *B. barnesiae,* and *C. leptum* abundances ($P < 0.05$, Wilcoxon rank-sum test) (**Supplementary Fig. S8a, b**) compared with the highest EN300 values (mean = 113.75) of the 20% of chickens in the reproductive tract sites. Although its function is unknown, 20% of chickens with the highest abundance of *Firmicutes* bacterium ZOR0006 exhibited significantly lower EN300 values than the 20% of chickens with the lowest abundance of this microorganism (**Fig. 5a**) ($P < 0.05$, Wilcoxon rank-sum test) in the reproductive tract sites.

Moreover, the 20% of chickens with the highest *B. fragilis*, *B. salanitronis*, *B.*

barnesiae, and *C. leptum* abundances exhibited significantly higher EN300 values than the 20% of chickens with the lowest abundances of these microorganisms in the reproductive tract sites, with the exception of *C. leptum* abundance in the vagina (**Fig. 5a**). *C. leptum*, a major member of the *Firmicutes* phylum, can alter the gut microbiota in rats, especially in obese individuals. In human infants, fecal levels of *C. leptum* were found to be negatively correlated with proinflammatory marker levels [62]. Colonic colonization of *C. leptum* was associated with accumulation of regulatory T cells, which inhibited the development of inflammatory lesions. The proliferation and activation of regulatory T cells is crucial to establishing and maintaining an appropriate level of immune tolerance. In addition, our results demonstrated that *C. leptum* was associated with a large range of other uterus or isthmus microbiota constituents (but limited association was observed with digestive microbiota constituents) and was not influenced by host genetics. Thus, this microorganism might serves as a stimulator of regulatory T cell production and inhibitor of inflammatory lesions, then regulating and maintaining immunologic tolerance and microbiota composition of the reproductive tract (especially the uterus and isthmus). These results suggest that the microbial species contribute to the enhanced egg production are modulated by influencing the immune processes.

We then characterized the spatial distribution of these five EN300-associated microorganisms (*B. fragilis*, *B. salanitronis*, *B. barnesiae*, *C. leptum*, and *Firmicutes* bacterium ZOR0006). *B. fragilis* was detected in almost all samples and accounted for 0.05% - 1.29% of the total abundance (**Fig. 5b**). *B. salanitronis* and *B. barnesiae* were detected at similar ratios in the six sites and in most samples from the reproductive tract sites; both accounted for the highest abundance in the vagina. *Firmicutes* bacterium ZOR0006 was also detected in most samples from the reproductive tract sites (74.22%

- 89.84%) and in half of the samples from the digestive tract sites (48.44% - 58.59%), accounting for 0.61% - 2.40% of the total abundance. Although the detection ratio (28.13% - 64.84%) and relative abundance of *C. leptum* were much lower than those of other microorganisms in all six sites, they accounted for the highest abundance in the isthmus and uterus (**Fig. 5b**).

Transcriptomic divergence in the uterus between hens with high and low egg production

To test whether microorganisms influence egg production by modulating immune processes, we compared the transcriptional profiles in the uterus between the two groups composed of hens with either the 20% lowest or 20% highest EN300 values (six hens for each group). As expected, the correlation rates between the high- and low-egg production groups (mean Pearson's $r = 0.93$) were relatively lower than those between biological replicates (mean Pearson's $r = 0.95$ and 0.96 for groups with high and low egg production, respectively) (**Fig. 6a, b**), indicating significant biological differences between groups. We identified 1,051 genes that exhibited significant expression changes (FDR ≤ 0.01 and $|\log2\ (\text{fold change})| \geq 1$) between groups with high and low egg production (**Fig. 6c**), which are mainly involved in immune-related categories, including the 'NF−κB signaling pathway' and 'chronic inflammatory response' (**Supplementary Fig. S9**). Of these, 739 genes that were significantly downregulated in the high-egg production group were overrepresented in the categories related to the inflammatory response, including 'T cell costimulation', 'B cell receptor signaling pathway' and 'lymphocyte activation' (**Fig. 6e**). Notably, we observed eight well-documented inflammatory markers (two Toll-like receptors [*TLR15* and *TLR1A*] and

six interleukins [*IL21R*, *IL18RAP*, *IL22RA2*, *IL4I1*, *IL17REL* and *IL8*]) (**Fig. 6c, d**) that were significantly downregulated in the uterus of the high-egg production group compared to the low-egg production group. Because ectopic expression of these inflammatory genes in the endometrium has been associated with the development of endometritis and thus results in infertility and subfertility [63-65], repressive transcription of these genes is beneficial for egg production. This result suggests the possible mechanism by which the downregulated expression of well-documented inflammatory markers suppresses the inflammatory response (and thus may contribute to the benign immune state) in reproductive tracts, which potentially benefits egg production. Functionally, the microbiota of the uterus affect the health of the oviduct and thus influence chicken egg production, which is manifested as increased pathway abundance for bacterial motility proteins, the bacterial secretion system, and membrane and intracellular structural molecules.

Conclusion

Our study provides a comprehensive view of the microbiome succession in the digestive and reproductive tracts of layer chickens. The diversity, composition, and predicted function of the microbiota varied considerably according to location within the reproductive and digestive tracts. Our results indicate that the reproductive tract microbiota in the hen influences egg production more than the digestive tract microbiota, and host genome has limited effect on their microbial composition. A small proportion of the variability in egg production was associated with the microbiota in the reproductive and digestive tracts of chickens. Remarkably, the genus *Bacteroides* and the species *C. leptum* and *Firmicutes* bacterium ZOR0006 were strongly associated with egg production, indicating their potential role in promoting reproductive

performance. Reproductive tract bacterial species could suppress the inflammatory response and thus potentially benefit for the egg production. These findings provide new insight into the roles of reproductive and digestive tract microbiota for complex traits, and may help contribute to the development of effective therapies for improving commercial egg production in chickens.

Figure Legends

Figure 1. Diversity and composition of the reproductive and digestive tract microbiota in chickens. (**a)** Relative abundance of the microbiota from six sites at the genus level. Only genera with an abundance > 1% in a site are shown. (**b)** Alpha diversity comparison based on the Shannon diversity index (*P < 0.05; **P < 0.01, Wilcoxon rank-sum test). **c.** Principal coordinates analysis of the 768 samples based on weighted UniFrac distances. 34.96% of variance was explained for component 1 (P < 0.05, Tracy-Widom test) and 24.23% for component 2 (P < 0.05, Tracy-Widom test).

Figure 2. Comparison of predicted functional capacities and site-associated taxa of microbial communities. (**a)** Heatmap showing the predicted KEGG pathways and their abundances at reproductive and digestive tract sites (Supplementary Table S7). Z scores indicate the means of KEGG pathway abundances. (**b)** Heatmap showing the 65 site-associated bacterial taxa identified by LEfSe (LDA > 4). Z scores indicate the relative abundances of site-associated bacterial taxa. Black frames represent site-associated bacterial taxa whose Z scores of relative abundances differed significantly among the six sites. p, phylum; c, class; o, order; f, family; g, genus.

Figure 3. Effect of host genetics on microbiota of the reproductive and digestive tracts. (**a-f)** Density scatter plots of genetic kinship of pairs of individuals (x axis) and their

microbiome dissimilarity (y axis) among all pairs of individuals (*n* = 16,256). NS, not significant (*P* > 0.05; Mantel test). (**g-i)** Distribution of species, genera, and OTUs identified in six sites for all chickens. Microorganisms present in < 20% of samples were excluded. (**j-l)** Proportion of heritable microbial genera, species, and OTUs in each site. **(m-o)** Cumulative relative abundances of heritable microbial genera, species, and OTUs in each site.

Figure 4. Microorganisms associated with egg number at 300 days of age (EN300). (**a)** Microbial species associated with EN300 (*P* < 0.05) among the six sites. **(b)** Pearson's *r* values between EN300 and 24 EN300-associated microbial species; only significant *r* values are given numerically (*P* < 0.05). **c** Pearson's *r* (lower diagonal) and Spearman's *r* (upper diagonal) values among microbial species in the uterus (UT); significant *r* values are given numerically (*P* < 0.05).

Figure 5. Effect of microbial species associated with EN300. (**a)** EN300 values for the 20% of chickens with the highest and lowest abundances of *B. fragilis*, *B. salanitronis*, *B. barnesiae*, *C. leptum*, and *Firmicutes* bacterium ZOR0006 in the three reproductive tract sites. The plots show the median, as well as the 25% and 75% quantiles. The cross and horizontal lines indicate the mean and median values in the corresponding group, respectively. Significance levels were calculated using a permutation test with 10,000 replicates. **P* < 0.05; NS, not significant. (**b)** Relative abundance and detected ratio of five species (*B. fragilis*, *B. salanitronis*, *B. barnesiae*, *C. leptum* and *Firmicutes* bacterium ZOR0006) in the six sites. Blue bars indicate the detection ratio of each species at each site.

Figure 6. Microorganisms influence the expression of immune-related genes in the uterus. **(a)** Hierarchical clustering and (**b)** pairwise Pearson's correlations of 12 samples

using transcriptional profiles. **(c)** Differentially expressed genes between groups with high and low egg production. Eight well-documented inflammatory markers are labeled. (**d)** Comparison of expression changes of eight inflammatory markers in the uterus between groups with high and low egg production by a q-PCR approach. **(e)** Top 20 functional categories enriched for 739 genes that were significantly downregulated in the high-egg production group. The enrichment analysis was performed using the Metascape tool (see Method). GO-BP: biological process (blue), GO-MF: molecular function (yellow) and KEGG (red).

Availability of supporting data

The sequencing data for this project have been deposited in the National Genomics Data Center (https://bigd.big.ac.cn/) under accession numbers CRA002196 (microbiome), CRA002195 (Whole genome resequencing) and CRA003376 (RNA-seq).

Additional Files

Supplementary Figure S1. Distribution of egg number at 300 days of age (EN300). Compared to the 20% of hens with the highest EN300 values, the 20% of hens with the lowest EN300 values exhibited a later start laying age, an earlier stop aging day and irregular lay performance.

Supplementary Figure S2. Quality assessment of sequencing data, alpha diversity, relative abundance, and Spearman's *r* values of specific microbiota among the six sites. (**a**) Length distribution of reads, quality score of each base, quality score distribution of sequencing data and error rate distribution of reads. (**b-f**) Alpha diversity comparison

based on Good's coverage, observed OTU，ACE, Chao1, and Simpson indices, using Wilcoxon rank-sum test to determine significant differences. (**g**) Rarefaction curves of observed OTU. (**h**) Alpha diversity values of the six sites. Values are represented as median±SD. (**i**) *P* values of Wilcoxon rank-sum test of each comparison for six alpha diversity indices. (**j**) PCoA of the 768 samples based on unweighted UniFrac distances. (**k**) Relative abundance of the top ten dominant microbial phyla in the six sites. (**l**) Only microbial genera that were present in at least 461 samples (60% of the total) were plotted. Each row represents a microorganism. Among 2,475 Spearman's *r* values, only 362 (14.62%) were significantly correlated ($P < 0.05$).

Supplementary Figure S3. Comparison of the functional capacities of the reproductive and digestive microbial communities among the six sites. (**a**) Overlap of the top 50 predictions among the six sites. (**b**) Heatmap showing the 36 overlapped predictions with different abundances among the six sites. The heatmap is color-coded based on row *Z* scores. (**c**) Map showing 65 site-associated bacterial taxa identified by LEfSe (LDA score > 4) in the test trial.

Supplementary Figure S4. The number distribution of OTUs with different existing ratio of samples and significantly heritable microorganisms. (**a**) The number plot of OTUs with different existing ratio of samples. (**b**) The relationship of existing ratio and the slope of the curve in A. The dotted line indicated the threshold utilized to remove existing ratio distribution trend due to fluctuate greatly. The number of significantly heritable microorganism OTUs, genera, and species ($P < 0.05$) grouped by sampling phyla (**c, e, g**) and site (**d, f, h**).

Supplementary Figure S5. Significantly EN300-associated microorganisms. The number of microorganisms significantly associated with EN300 detected at OTU, genus, and species ($P < 0.05$) levels grouped by sampling phyla (**a, c, e**) and site (**b, d, f**).

Supplementary Figure S6. EN300-associated microorganisms. (**a**) The number of microbial genera (left) and OTUs (right) associated with EN300 at $P < 0.05$ of three test methods of six sites and their overlap. (**b-c**) Pearson's and Spearman's *r* values between EN300 and EN300-associated 26 genera and 39 OTUs. Red and blue tiles indicate positive and negative correlations, respectively. Significant *r* values are filled in numerically ($P < 0.05$).

Supplementary Figure S7. Pearson correlations between EN300 and EN300-associated microorganisms. (**a**) Pearson' s *r* values of candidate microbial species in the six sites. (**b**) Pearson' s *r* values among microbial species in each site. CP: Crop, GZ: Gizzard, SI: small intestine, UT: uterus, IS: Isthmus, VA: vagina. Red and blue tiles indicate positive and negative correlations, respectively. The ratios on the right side of each site represents the number of significant correlations. *$P < 0.05$.

Supplementary Figure S8. Differences in the relative abundance of five species between the 20% of chickens with the highest and lowest egg production (EN300). (**a**) EN300 values for the 20% of individuals with the highest and lowest egg production. (**b**) EN300 values for the 20% of individuals with the highest and lowest abundances of *Clostridium leptum*, *Bacteroides salanitronis*, *Firmicutes* bacterium ZOR0006, *B. barnesiae*, and *B. fragilis* in the three reproductive tract sites. All comparisons were significantly different, established at $P < 0.05$.

Supplementary Figure S9. Top 20 functional categories enriched by 1,051 genes exhibited significant expression changes between groups with the high- and low- egg production. The enrichment analysis was performed using the Metascape tool (See Method). GO-BP: biological process (blue), GO-MF: molecular function (yellow) and KEGG (red).

Supplementary Table S1. Summary of 16S rRNA gene sequencing.

Supplementary Table S2. Summary statistics of 16S rRNA gene sequencing.

Supplementary Table S3. Summary of host whole genome sequencing.

Supplementary Table S4. Summary statistics of host whole genome sequencing.

Supplementary Table S5. Primer sequences for q-PCR.

Supplementary Table S6. Analysis of Bray-Curtis distance similarities.

Supplementary Table S7. Statistical test for the 65 functional capacities among the six sites.

Supplementary Table S8. Correlation between genetic relatedness matrix (GRM) and each Bray-Curtis (BC) distance or microbial relationship matrix (MRM) by Mantel test.

Supplementary Table S9. Heritability (h^2) of the microbiota and cumulative abundance of heritable microbiota.

Supplementary Table S10. Heritability (*h2*) of reproductive traits from previous reports.

List of abbreviations

QTLs: quantitative trait loci; h^2: heritability; GWAS: genome-wide association study; *PLAG1*: pleiomorphic adenoma gene 1; *LYN*: lck/yes-related novel tyrosine kinase gene; EN300: egg number at 300 days of age; M: million; OTUs: operational taxonomic units; BC: Bray-Curtis; PCoA: Principal coordinates analysis; FDR: false discovery rate; ANOSIM: Analysis of Similarity; LDA: Linear discriminant analysis; LEfSe: Linear discriminant analysis Effect Size; BWA: Burrows-Wheeler Alignment tool; GATK: Genome Analysis Toolkit; SNP: single nucleotide polymorphism; MRM: microbial relationship matrix; PCs: principal components; GRM: genetic relatedness matrix; m^2: microbiability; LMM: linear mixed model; RNA-seq: RNA sequencing; TPM: transcripts per million; GO: Gene Ontology; KEGG: Kyoto Encyclopedia of Genes and Genomes; q-PCR: quantitative PCR; CPSA: capsular polysaccharide A.

Ethics approval

All animal experiments were approved and reviewed by Animal Care and Use Committee Institutional of Sichuan Agricultural University (Approval No. DKY-2018102015).

Competing interests

The authors declare no competing interests.

Acknowledgements

We thank the High-Performance Computing Platform of Sichuan Agricultural University and Ya'an Big Data Industrial Park for providing computing resources and support that have contributed to these research results. This work was supported by the Sichuan Science and Technology Program (2019JDTD0009, 2020YFH0138 and 2021YFYZ0009), the Fok Ying-Tong Education Foundation for Young Teachers in the Higher Education Institutions of China (161026).

Author contributions

D.L., M.L. and Q.Z. designed the study; T.W., S.K.M., Y.S., W.Z., and S.T. wrote the manuscript; Z.X., M.H., X.Z., H.Y., X.F., and Q.N. collected the sample and extracted the DNA. M.Y., D.Y., Y.L., B.Z, and M.Z., did bioinformatics analyses; D.L. wrote methods; D.L., M.L. and Q.Z. supervised the work.

References

1. Wolc A, Arango J, Settar P, et al. Analysis of egg production in layer chickens using a random regression model with genomic relationships. Poult Sci 2013;**92**(6):1486-1491.
2. Hu ZL, Park CA, Reecy JM. Building a livestock genetic and genomic information knowledgebase through integrative developments of Animal QTLdb and CorrDB. Nucleic Acids Res 2019;**47**(D1):D701-D710.
3. Yuan J, Sun C, Dou T, et al. Identification of Promising Mutants Associated with Egg Production Traits Revealed by Genome-Wide Association Study. PLoS One 2015;**10**(10):e0140615.
4. Zhang GX, Fan QC, Wang JY, et al. Genome-wide association study on reproductive traits in

Jinghai Yellow Chicken. Anim Reprod Sci 2015;**163**:30-34.

5. Tongsiri S, Jeyaruban MG, Van Der Werf JH. Genetic parameters for egg production traits in purebred and hybrid chicken in a tropical environment. Br Poult Sci 2015;**56**(6):613-620.
6. Savegnago RP, Caetano SL, Ramos SB, et al. Estimates of genetic parameters, and cluster and principal components analyses of breeding values related to egg production traits in a White Leghorn population. Poult Sci 2011;**90**(10):2174-2188.
7. Chen C, Song X, Wei W, et al. The microbiota continuum along the female reproductive tract and its relation to uterine-related diseases. Nat Commun 2017;**8**(1):875.
8. Wen C, Li Q, Lan F, et al. Microbiota continuum along the chicken oviduct and its association with host genetics and egg formation. Poult Sci 2021:101104.
9. Fettweis JM, Serrano MG, Brooks JP, et al. The vaginal microbiome and preterm birth. Nat Med 2019;**25**(6):1012-1021.
10. Serrano MG, Parikh HI, Brooks JP, et al. Racioethnic diversity in the dynamics of the vaginal microbiome during pregnancy. Nat Med 2019;**25**(6):1001-1011.
11. Den Hartog G, De Vries-Reilingh G, Wehrmaker AM, et al. Intestinal immune maturation is accompanied by temporal changes in the composition of the microbiota. Benef Microbes 2016;**7**(5):677-685.
12. Choi JH, Kim GB, Cha CJ. Spatial heterogeneity and stability of bacterial community in the gastrointestinal tracts of broiler chickens. Poult Sci 2014;**93**(8):1942-1950.
13. Lee S, La T-M, Lee H-J, et al. Characterization of microbial communities in the chicken oviduct and the origin of chicken embryo gut microbiota. Sci Rep 2019;**9**(1):6838.
14. Elokil AA, Magdy M, Melak S, et al. Faecal microbiome sequences in relation to the egg-laying

performance of hens using amplicon-based metagenomic association analysis. Animal 2020;**14**(4):706-715.

15. Carmody RN, Gerber GK, Luevano JM, Jr., et al. Diet dominates host genotype in shaping the murine gut microbiota. Cell Host Microbe 2015;**17**(1):72-84.
16. Rothschild D, Weissbrod O, Barkan E, et al. Environment dominates over host genetics in shaping human gut microbiota. Nature 2018;**555**(7695):210-215.
17. Liu H, Chen Z, Gao G, et al. Characterization and comparison of gut microbiomes in nine species of parrots in captivity. Symbiosis 2019;**78**(3):241-250.
18. Ji J, Luo CL, Zou X, et al. Association of host genetics with intestinal microbial relevant to body weight in a chicken F2 resource population. Poult Sci 2019;**98**(9):4084-4093.
19. Mignon-Grasteau S, Narcy A, Rideau N, et al. Impact of Selection for Digestive Efficiency on Microbiota Composition in the Chicken. PLoS One 2015;**10**(8):e0135488.
20. Scepanovic P, Hodel F, Mondot S, et al. A comprehensive assessment of demographic, environmental, and host genetic associations with gut microbiome diversity in healthy individuals. Microbiome 2019;**7**(1):130.
21. Caporaso JG, Lauber CL, Walters WA, et al. Global patterns of 16S rRNA diversity at a depth of millions of sequences per sample. Proc Natl Acad Sci U S A. 2011;**108**:4516-4522.
22. Martin M. Cutadapt removes adapter sequences from high-throughput sequencing reads. EMBnet J 2011;**17**(1):10-12.
23. Edgar RC, Haas BJ, Clemente JC, et al. UCHIME improves sensitivity and speed of chimera detection. Bioinformatics 2011;**27**(16):2194-2200.
24. Edgar RC. UPARSE: highly accurate OTU sequences from microbial amplicon reads. Nat.

Methods 2013;**10**(10):996-998.

25. Quast C, Pruesse E, Yilmaz P, et al. The SILVA ribosomal RNA gene database project: improved data processing and web-based tools. Nucleic Acids Res 2013;**41**:D590-D596.
26. Kozich J, Westcott S, Baxter N, et al. Development of a dual-index sequencing strategy and curation pipeline for analyzing amplicon sequence data on the MiSeq Illumina sequencing platform. Appl Environ Microbiol 2013;**79**(17):5112-5120.
27. Edgar RC. MUSCLE: multiple sequence alignment with high accuracy and high throughput. Nucleic Acids Res 2004;**32**(5):1792-1797.
28. Bolyen E, Rideout J, Dillon M, et al. Reproducible, interactive, scalable and extensible microbiome data science using QIIME 2. Nat Biotechnol 2019;**37**(8):852-857.
29. Langfelder P, Horvath S. WGCNA: an R package for weighted correlation network analysis. BMC Bioinformatics 2008;**9**:559.
30. Douglas GM, Maffei VJ, Zaneveld J, et al. PICRUSt2: An improved and extensible approach for metagenome inference. BioRxiv 2019:672295.
31. Segata N, Izard J, Waldron L, et al. Metagenomic biomarker discovery and explanation. Genome Biol 2011;**12**(6):R60.
32. Li M, Tian S, Jin L, et al. Genomic analyses identify distinct patterns of selection in domesticated pigs and Tibetan wild boars. Nat Genet 2013;**45**(12):1431-1438.
33. Li H, Durbin R. Fast and accurate long-read alignment with Burrows–Wheeler transform. Bioinformatics 2010;**26**(5):589-595.
34. McKenna A, Hanna M, Banks E, et al. The Genome Analysis Toolkit: a MapReduce framework for analyzing next-generation DNA sequencing data. Genome Res 2010;**20**(9):1297-1303.

35. Li H, Ruan J, Durbin R. Mapping short DNA sequencing reads and calling variants using mapping quality scores. Genome Res 2008;**18**(11):1851-1858.

36. Wang K, Li M, Hakonarson H. ANNOVAR: functional annotation of genetic variants from high-throughput sequencing data. Nucleic Acids Res 2010;**38**(16):e164.

37. Camarinha-Silva A, Maushammer M, Wellmann R, et al. Host Genome Influence on Gut Microbial Composition and Microbial Prediction of Complex Traits in Pigs. Genetics 2017;**206**(3):1637-1644.

38. Purcell S, Neale B, Todd-Brown K, et al. PLINK: a tool set for whole-genome association and population-based linkage analyses. Am J Hum Genet 2007;**81**(3):559-575.

39. Yang J, Bakshi A, Zhu Z, et al. Genetic variance estimation with imputed variants finds negligible missing heritability for human height and body mass index. Nat Genet 2015;**47**(10):1114-1120.

40. Yang J, Lee SH, Goddard ME, et al. GCTA: a tool for genome-wide complex trait analysis. Am J Hum Genet 2011;**88**(1):76-82.

41. Zierer J, Jackson MA, Kastenmuller G, et al. The fecal metabolome as a functional readout of the gut microbiome. Nat Genet 2018;**50**(6):790-795.

42. Zhou X, Stephens M. Genome-wide efficient mixed-model analysis for association studies. Nat Genet 2012;**44**(7):821-824.

43. Bray NL, Pimentel H, Melsted P, et al. Near-optimal probabilistic RNA-seq quantification. Nat Biotechnol 2016;**34**(5):525-527.

44. Robinson MD, McCarthy DJ, Smyth GK. edgeR: a Bioconductor package for differential expression analysis of digital gene expression data. Bioinformatics 2010;**26**(1):139-140.

45. Zhou Y, Zhou B, Pache L, et al. Metascape provides a biologist-oriented resource for the analysis of systems-level datasets. Nat Commun 2019;**10**(1):1523.

46. Verstraelen H, Vilchez-Vargas R, Desimpel F, et al. Characterisation of the human uterine microbiome in non-pregnant women through deep sequencing of the V1-2 region of the 16S rRNA gene. PeerJ 2016;**4**:e1602.

47. Mishra AK, Kumar A, Swain DK, et al. Insights into pH regulatory mechanisms in mediating spermatozoa functions. Vet World 2018;**11**(6):852-858.

48. Fiser PS, Macpherson JW. pH values in the oviduct of the hen during egg formation. Poult Sci 1974;**53**(2):827-829.

49. Yu MD, Lai EM. Warfare between Host Immunity and Bacterial Weapons. Cell Host Microbe 2017;**21**(1):3-4.

50. Moore RW, Park SY, Kubena LF, et al. Comparison of zinc acetate and propionate addition on gastrointestinal tract fermentation and susceptibility of laying hens to Salmonella enteritidis during forced molt. Poult Sci 2004;**83**(8):1276-1286.

51. Kalisperati P, Spanou E, Pateras IS, et al. Inflammation, DNA Damage, Helicobacter pylori and Gastric Tumorigenesis. Front Genet 2017;**8**:20.

52. Liu J, Yue S, Yang Z, et al. Oral hydroxysafflor yellow A reduces obesity in mice by modulating the gut microbiota and serum metabolism. Pharmacol Res 2018;**134**:40-50.

53. Moreira G, Sobrinho APR, Bambirra BHS, et al. Synergistic Growth Effect among Bacteria Recovered from Root Canal Infections. Braz J Microbiol 2011;**42**(3):973-979.

54. Jeong DY, Cho SK, Shin HS, et al. Application of an electric field for pretreatment of a seeding source for dark fermentative hydrogen production. Bioresour Technol 2013;**139**:393-396.

55. Marche MG, Satta A, Floris I, et al. Quantitative variation in the core bacterial community associated with honey bees from Varroa- infested colonies. J Apicult Res 2019;**58**(3):444-454.

56. Chousalkar KK, Roberts JR. Ultrastructural changes in the oviduct of the laying hen during the laying cycle. Cell Tissue Res 2008;**332**(2):349-358.

57. Hrabia A, Lesniak-Walentyn A, Sechman A, et al. Chicken oviduct-the target tissue for growth hormone action: effect on cell proliferation and apoptosis and on the gene expression of some oviduct-specific proteins. Cell Tissue Res 2014;**357**(1):363-372.

58. Salanitro JP, Blake IG, Muirhead PA. Studies on the cecal microflora of commercial broiler chickens. Appl Microbiol 1974;**28**(3):439-447.

59. Reeves AR, Wang GR, Salyers AA. Characterization of four outer membrane proteins that play a role in utilization of starch by Bacteroides thetaiotaomicron. J Bacterioly 1997;**179**(3):643-649.

60. Nihira T, Suzuki E, Kitaoka M, et al. Discovery of beta-1,4-D-mannosyl-N-acetyl-D-glucosamine phosphorylase involved in the metabolism of N-glycans. J Biol Chem 2013;**288**(38):27366-27374.

61. Mazmanian SK, Liu CH, Tzianabos AO, et al. An immunomodulatory molecule of symbiotic bacteria directs maturation of the host immune system. Cell 2005;**122**(1):107-118.

62. Partty A, Kalliomaki M, Salminen S, et al. Infantile Colic Is Associated With Low-grade Systemic Inflammation. J Pediatr Gastr Nutr 2017;**64**(5):691-695.

63. Shan H, Fischer DP, Dirk W, et al. Expression and Function of Toll-Like Receptor 4 in the Endometrial Cells of the Uterus. Endocrinology 2006;**147**(1):562-570.

64. Herath S, Lilly ST, Santos NR, et al. Expression of genes associated with immunity in the

endometrium of cattle with disparate postpartum uterine disease and fertility. Reprod Biol Endocrinol 2009;**7**:55.

65. Kannaki TR, Shanmugam M, Verma PC. Toll-like receptors and their role in animal reproduction. Anim Reprod Sci 2011;**125**(1-4):1-12.

Figure 1

Click here to access/download;Figure;Figure 1.tif

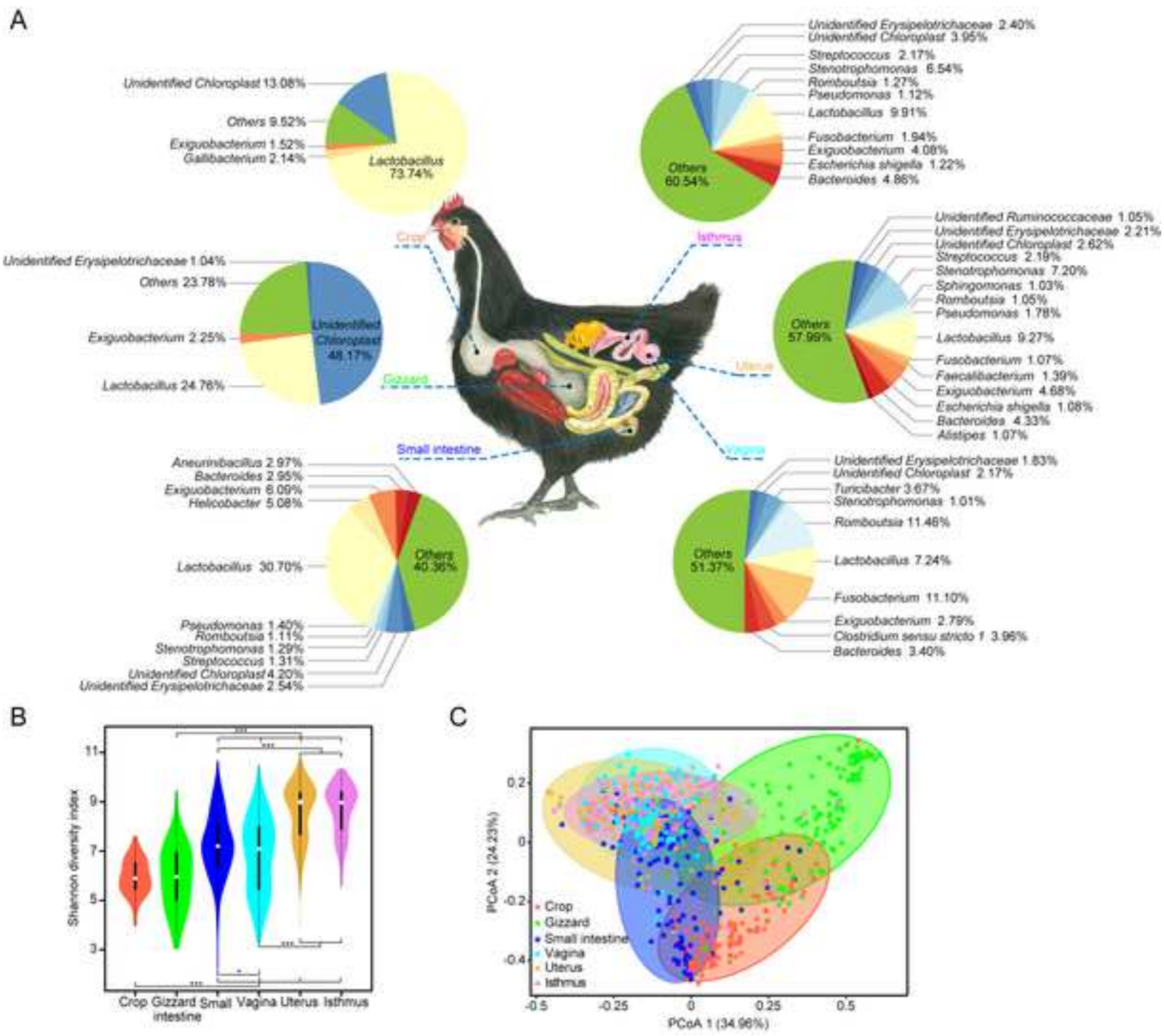

Figure 2

Click here to access/download;Figure;Figure 2.tif

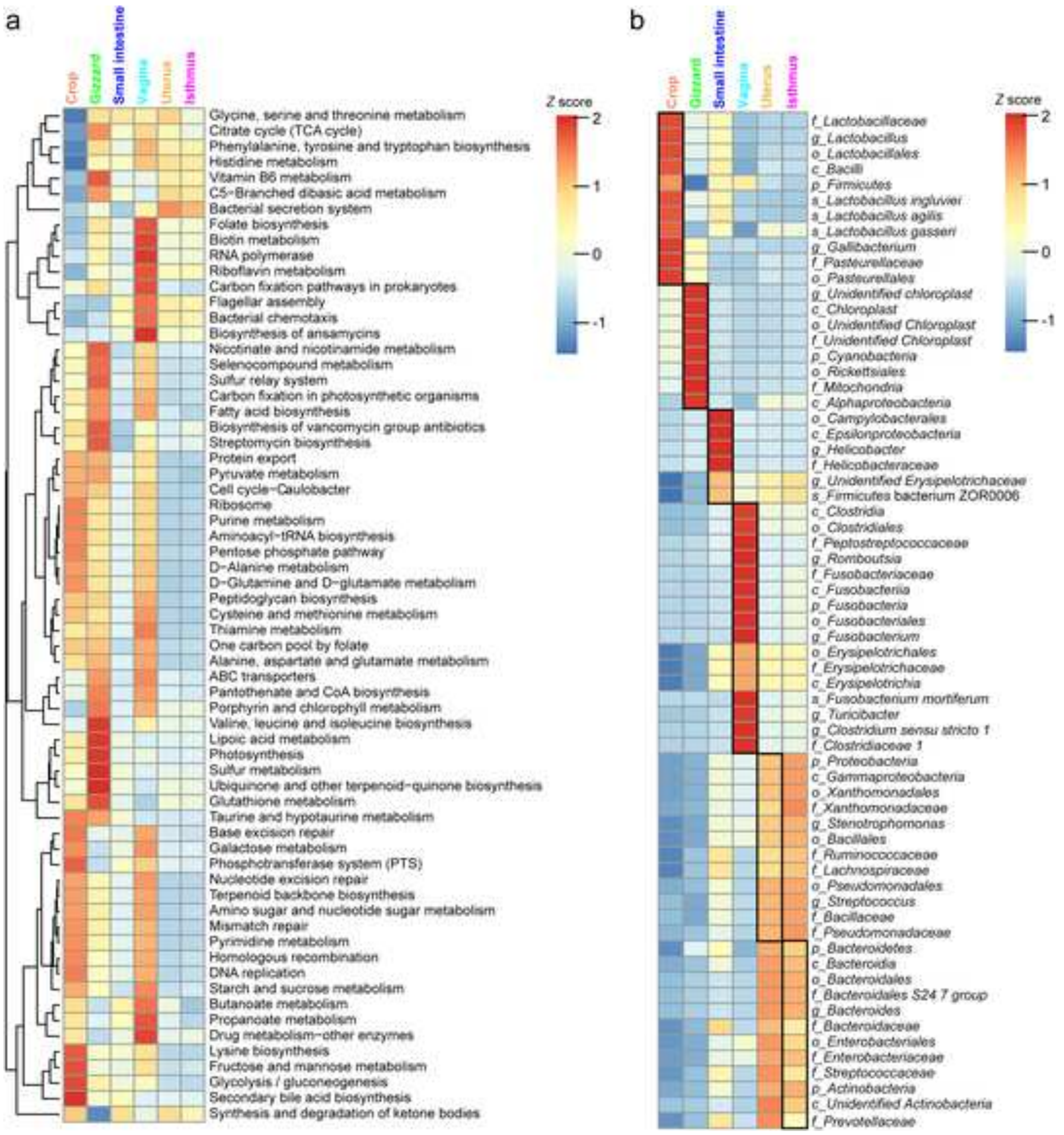

Figure 3 Click here to access/download;Figure;Figure 3.tif

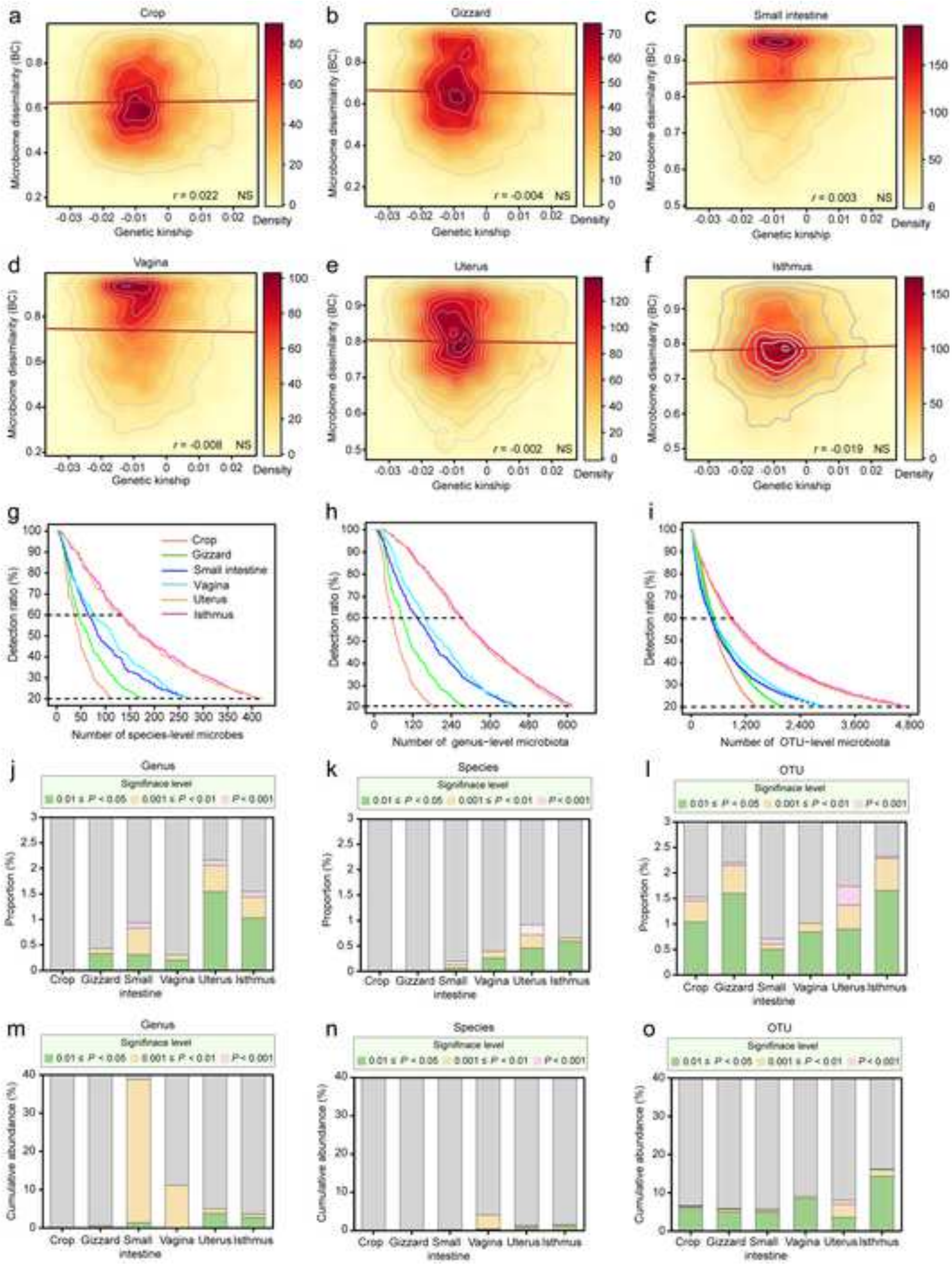

Figure 4

Click here to access/download;Figure;Figure 4.tif

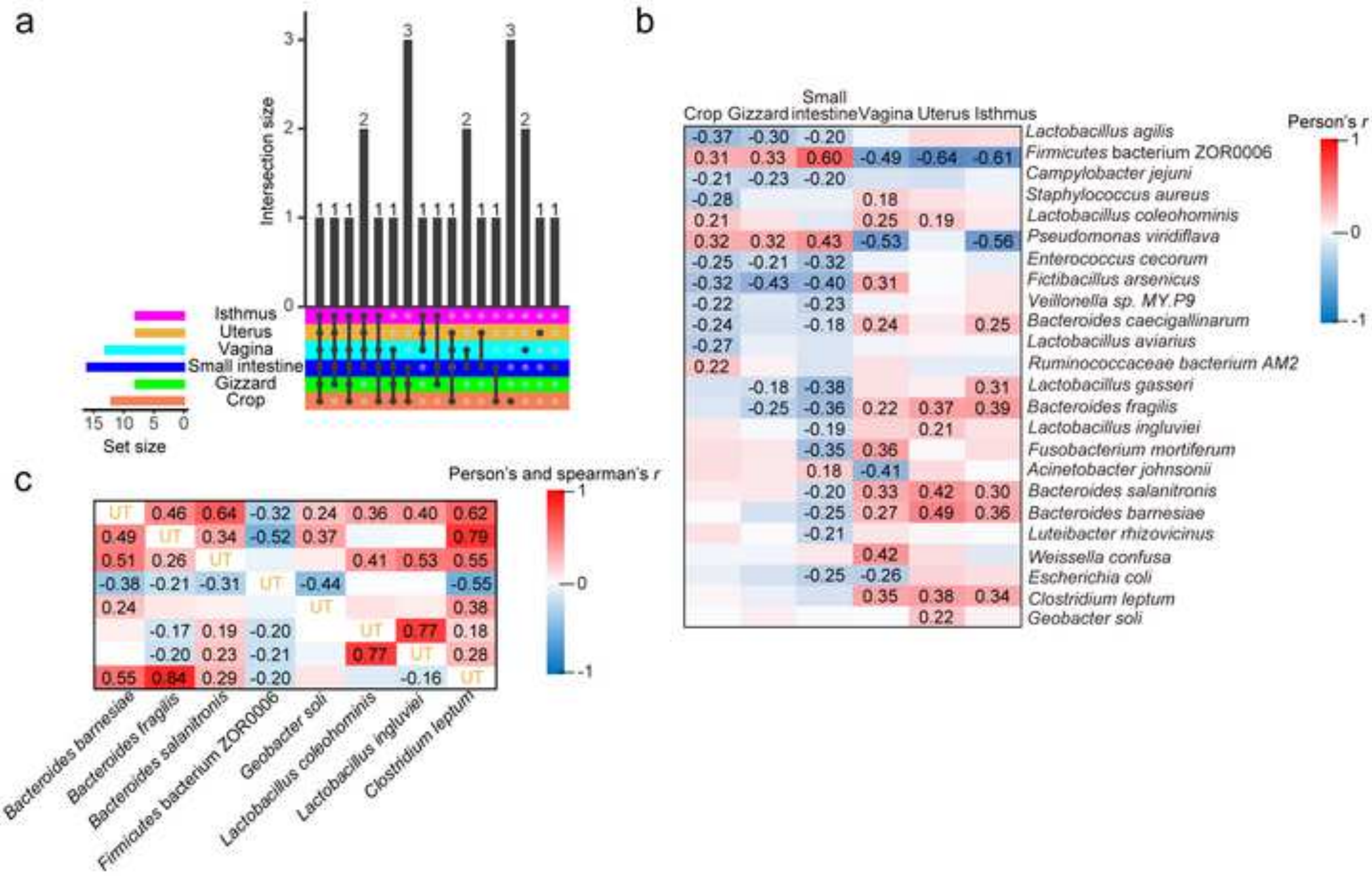

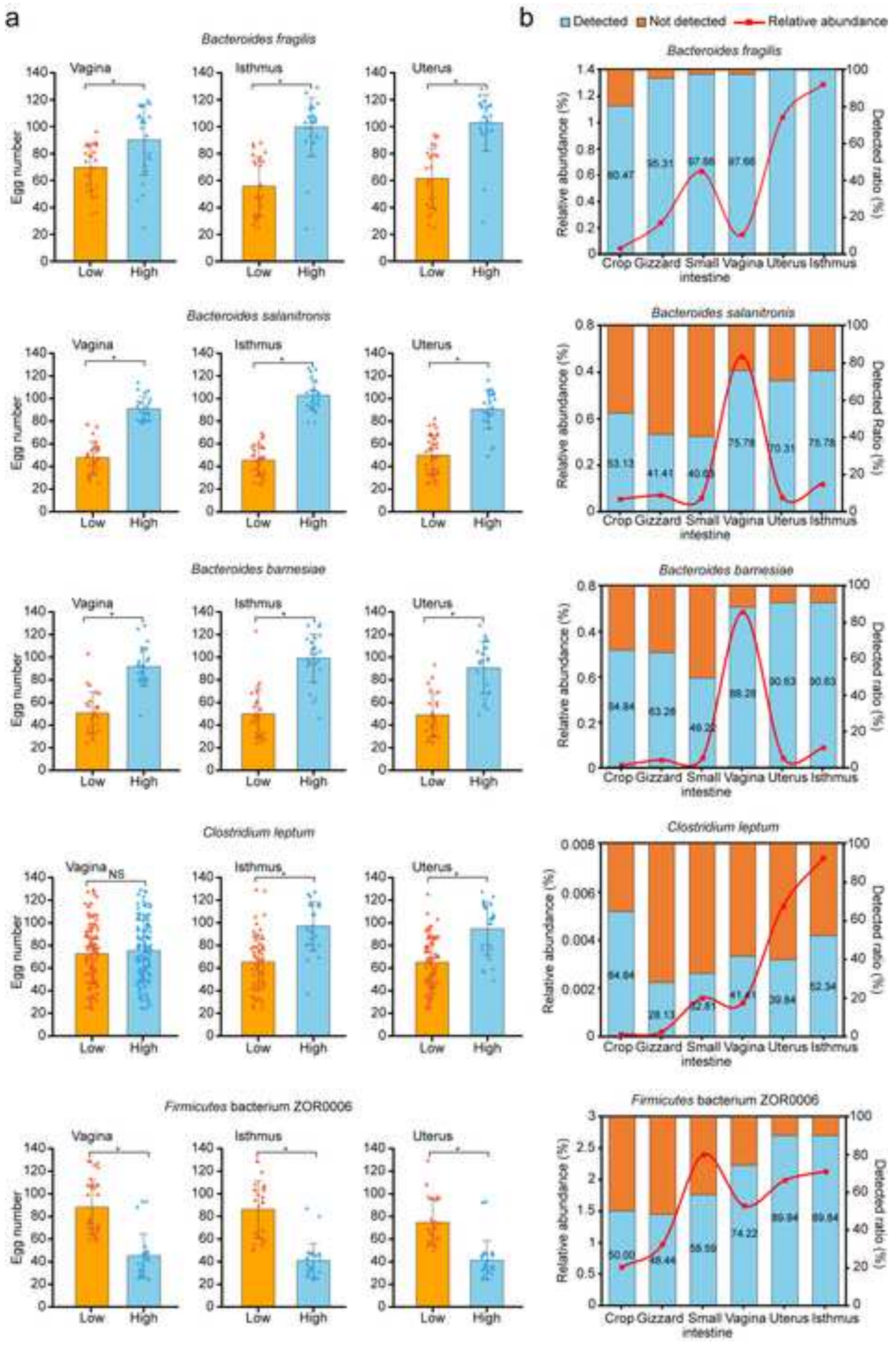
a
b
Detected Not detected Relative abundance
Bacteroides fragilis
Bacteroides salanitronis
Bacteroides barnesiae
Clostridium leptum
Firmicutes bacterium ZOR0006
Vagina
Isthmus
Uterus
Egg number
Low
High
Relative abundance (%)
Detected ratio (%)
Crop Gizzard Small intestine Vagina Uterus Isthmus

Figure 6

Click here to access/download;Figure;Figure 6.tif

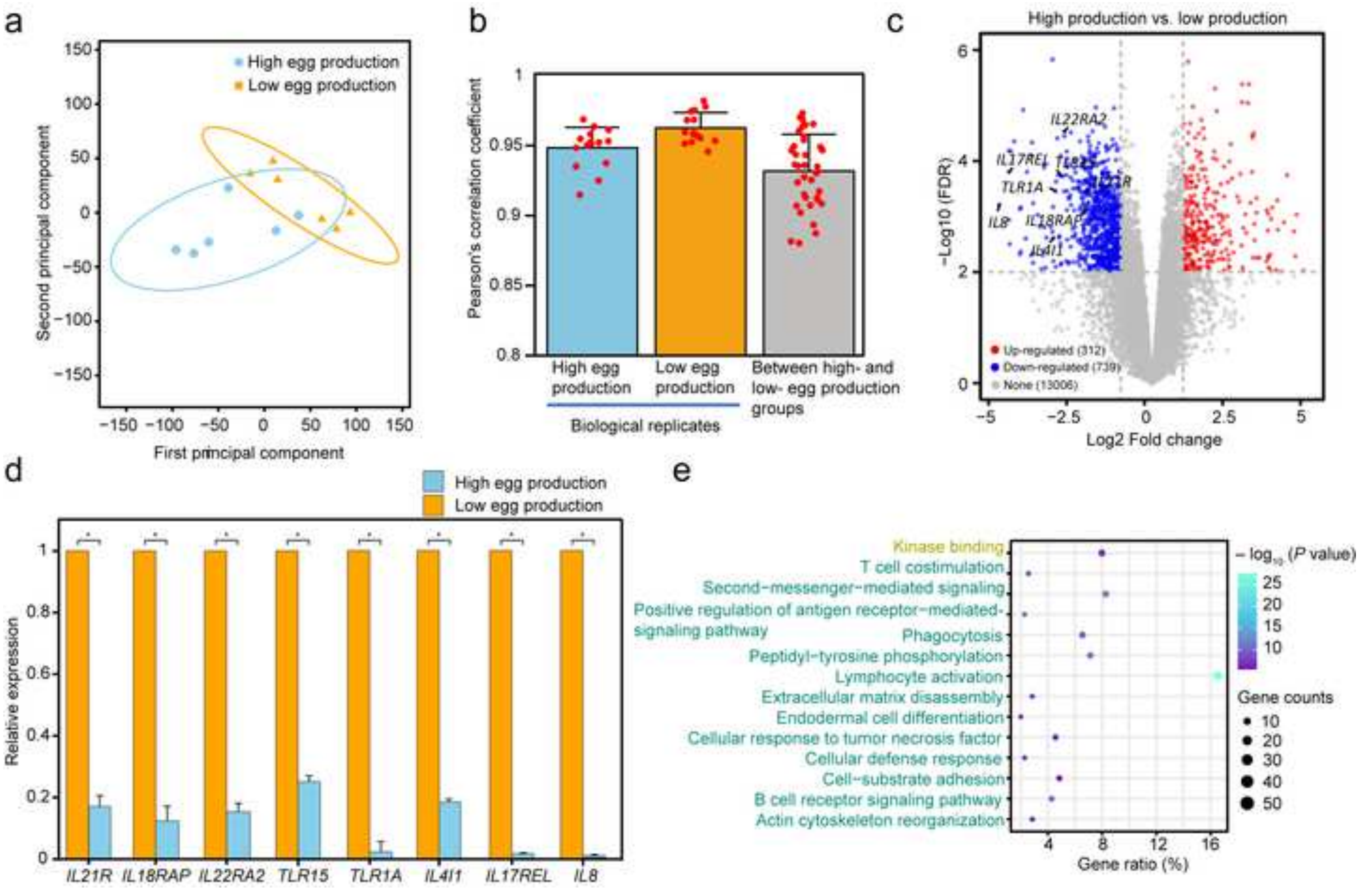

Supplementary Figures

Click here to access/download
Supplementary Material
Supplementary Figures.docx

Supplementary Tables

Click here to access/download
Supplementary Material
Supplementary Tables.xlsx
