# Supplementary material for: Association of female reproductive tract microbiota with egg production in layer chickens
Source: Gigascience. 2021 Sep 23;10(9):giab067. doi: 10.1093/gigascience/giab067 (PMC8460357; doi:10.1093/gigascience/giab067)
Supplement: giab067_GIGA-D-21-00132_Revision_1 [file giab067_giga-d-21-00132_revision_1.pdf]

GigaScience

Female reproductive tract microbiota influence egg production in layer chickens

--Manuscript Draft--

**Manuscript Number:**	GIGA-D-21-00132R1
**Full Title:**	Female reproductive tract microbiota influence egg production in layer chickens
**Article Type:**	Research
**Funding Information:**	Department of Science and Technology of Sichuan Province (2019JDTD0009) — Pro. Diyan Li<br>Fok Ying Tong Education Foundation (161026) — Pro. Diyan Li<br>Department of Science and Technology of Sichuan Province (2020YFH0138) — Dr. Zhongxian Xu<br>Department of Science and Technology of Sichuan Province (2021YFYZ0009) — Pro. Mingzhou Li
**Abstract:**	Background: The microbiota of the female reproductive tract is increasingly recognized as having fundamental roles in animal reproduction. To explore the relative contribution of reproductive tract microbiomes on egg production in chickens, we investigated the microbiota in multiple reproductive and digestive tract sites from 128 female layer (egg-producing) chickens in comparable environments.<br>Results: We identified substantial differences between the diversity, composition, and predicted function of site-associated microbiota. Reproductive tract microbiota were more profoundly affected egg production than that in the digestive tract. We identified four reproductive tract microbial species, Bacteroides fragilis , B. salanitronis , B. barnesiae, and Clostridium leptum , which were related to immune function and potentially contribute to enhanced egg production.<br>Conclusions: These findings provide insights into the diverse microbiota characteristics of reproductive and digestive tracts, and may help design strategies for controlling and manipulating chicken reproductive tract microbiota to improve egg production.
**Corresponding Author:**	Diyan Li<br><br>CHINA
**Corresponding Author Secondary Information:**	
**Corresponding Author's Institution:**	
**Corresponding Author's Secondary Institution:**	
**First Author:**	Yuan Su
**First Author Secondary Information:**	
**Order of Authors:**	Yuan Su<br>Shilin Tian<br>Diyan Li<br>Wei Zhu<br>Tao Wang<br>Shailendra Kumar Mishra<br>Ranlei Wei<br>Zhongxian Xu

Powered by Editorial Manager® and ProduXion Manager® from Aries Systems Corporation

	Mengnan He
	Xiaoling Zhao
	Huadong Yin
	Xiaolan Fan
	Bo Zeng
	Mingyao Yang
	Deying Yang
	Qingyong Ni
	Yan Li
	Mingwang Zhang
	Qing Zhu
	Mingzhou Li
**Order of Authors Secondary Information:**	
**Response to Reviewers:**	Dear editor and reviewers,<br><br>Thank you very much for reviewing our manuscript “Female reproductive tract microbiota influence egg production in layer chickens” (ID: GIGA-D-21-00132) for possible publication in GigaScience. We sincerely thank the editor and two reviewers for their valuable feedback that we have used to improve the quality of our manuscript. According to the reviewers’ comments, we have made the relevant modifications to our manuscript. All modifications are marked in red in the revised manuscript and a point-by-point response to the reviewers’ comments follows. We hope these revisions meet your satisfaction and make our manuscript acceptable for publication in your journal.<br><br>We look forward to hearing a positive response from you.<br><br>Best regards,<br>Diyan Li, Professor, Ph.D.<br>Address: Institute of Animal Genetics and Breeding, College of Animal Science and Technology, Sichuan Agricultural University, Chengdu 611130, China.<br>E-mail: diyanli@sicau.edu.cn<br><br>Detailed responses to reviewers:<br>All comments provided by reviewers are in black, and our responses are in red. This following text is also included in the uploaded file "Response letter":<br><br>Reviewer #1: Dear authors, the work described in the manuscript is very extensive! I have not yet seen an analysis of host genetics, microbiota composition and host transcriptomics coupled with egg production data. Also while not perfect the manuscript is written well.<br>Response: Thank you for your positive comments.<br><br>Main problems:<br>1.It is clear you found an association between specific bacteria and egg production. You have also shown that some form of inflammation is associated with the changes in microbiota and egg production. However, you assume that the bacteria affect inflammation which affect egg production. But other options also exist. For example, it is possible that waning egg production, from other causes, changes the conditions in the oviduct so that the microbiota would change. Or inflammation, for example because of a pathogen, might modulate both egg production as well as microbiota composition. Please change the text so that the readers understand you are speculating and briefly mention the other options.<br>Response: As suggested, we have added discussion of the other options affecting egg production in the "Results and Discussion" sections (Lines 355-363).<br>"Multiple factors, especially host species, potential pathogens, and immune status of the host, all play a major role in the female reproductive organs adversely interfering

Powered by Editorial Manager® and ProduXion Manager® from Aries Systems Corporation

with the egg industry in laying flocks [53]. Additionally, the digestive tract environment of low-egg producing hens is fragile and susceptible to the influence of exogenous microorganisms [41]. Pathogenic infection, room temperature fluctuations, management systems, and other sudden changes to various factors can alter the composition of microbiota [54, 55]. These alterations may cause a significant degradation in production performance. Here, our results indicate that the reproductive tract microbiota play an important role in egg production."

2.While it is too late to change, it is a pity that you did not characterize the cecum community. Of all of the intestinal communities the cecum is by far the biggest and the most likely to affect the nutrition of the hen, thereby possibly affecting egg production.
Response: Thank you for your comments and understanding. We have included some information regarding this in the Discussion (Lines 288-291).
"The cecum has distinct microbial community profiles [44-46] that were not explored in this study. Microbial community analysis of the cecum microbiota in chickens exhibiting different egg production performances requires further investigation."

3.Please make sure to reference and discuss relevant literature. I quickly identified Elokil A. A. Animal 2020 which performed a limited but similar analysis. Please perform a literature search and make sure to reference and discuss relevant work.
Response: Thank you for your helpful suggestions. As suggested, we have added and discussed additionally relevant literature references (Lines 280-288).
"In accordance with previous findings, there is clear evidence of the role of fecal microbiomes in low and high egg-laying performance in hens; Elokil et al [16] demonstrated a significantly positive association between the microbial genus Lactobacillus and egg-laying performance ($P < 0.05$). Likewise, Wang et al [41] reported that Lactobacillus was also abundant in the feces of high-yield hens. The genus Lactobacillus produces growth promoters and exhibits antimicrobial activity against pathogenic microbes [42, 43] which may explain why the increasing abundance of Lactobacillus in the high-yield group is beneficial to egg-laying performance."

Minor problems:
Lines 48-49 - something is wrong with this sentence.
Response: Thank you for the pointing this out. We have revised the sentence in the manuscript (Lines 46-48).
"An abnormal vaginal microbiota may predispose individuals to increased microbial invasion of the amniotic cavity and fetal damage [11, 12]."

Lines 67-69 - it is not clear what you are trying to say here.
Response: As commented, the sentence was not clear and had limited relevance to the manuscript so we deleted the sentence.

Line 71 - 16S rDNA sequencing and not whole-genome sequencing.
Response: In this study, we conducted whole-genome sequencing on 128 laying hens and 16S rDNA sequencing on 768 samples from six sites. We have revised the sentence in the manuscript (Lines 68-71).
"Here, we performed 16S rDNA sequencing on 768 samples from three reproductive (vagina, uterus, and isthmus) and three digestive (crop, gizzard, and small intestine) tract sites and whole-genome sequencing of 128 laying hens."

Line 96 - please be more specific regarding which part of the small intestine was sampled.
Response: The jejunum of the small intestine was sampled. As suggested, we provide a more specific explanation of which part of the small intestine was sampled in the revised manuscript (Lines 420-421).
"A 12-cm-long fixed mid-region of the small intestine (jejunum) was collected from each bird."

Lines 100-108 - description of host DNA extraction is not appropriate another the heading "microbial genomic DNA extraction".
Response: "Microbial genomic DNA extraction" has been changed to "Host and microbial genomic DNA extraction" in the revised manuscript (Line 426).

Powered by Editorial Manager® and ProduXion Manager® from Aries Systems Corporation

Line 109 and elsewhere - 16S rDNA and not 16S rRNA.
Response: "16S rRNA" was changed to "16S rDNA" throughout the whole manuscript.

Line 154 - why are you using OTUs with 97% identity and not 100% identical amplicon sequence variants (ASVs)?
Response: As an alternative to OTUs, ASVs have been proposed as a way to adapt the thresholds suggested by genome sequencing to microbial community analysis using 16S rDNA sequences. Meanwhile, the OTU approach is still one of the primarily used methods for analyzing 16S rDNA-seq data (Dvergedal et al., 2020; Pinna et al., 2021; Wen et al., 2021). Schloss PD recently evaluated the clustering risk among ASV and OTU methods, and reached a conclusion that ASVs and the use of overly narrow thresholds to identify OTUs increase the risk of splitting a single genome into separate clusters (Schloss, 2021).
Since there is no consensus for a biological definition of a bacterial species (Sanford et al., 2021), microbiologists should accept that how bacterial species are named is biased and that taxonomic rules are not applied in a consistent manner. This makes it impossible to fit a distance threshold that matches a set of species names (Konstantinidis and Tiedje, 2005). Furthermore, the 16S rDNA sequence does not evolve at the same rate across all bacterial lineages (Schloss and Westcott, 2011), which limits the biological interpretation of a common OTU definition. A distance-based definition of a taxonomic unit based on the 16S rDNA or full-genome sequences is operational and not necessarily grounded in biological theory (Yarza et al., 2014; Barco et al., 2020). One benefit of a distance-based OTU definition is the ability to mask residual sequencing errors. The sequences generated in microbiome studies can harbor PCR and sequencing errors. These errors would only exacerbate the inflated number of ASVs.
Although there are multiple reasons why proponents favor ASVs, we feel the significant risk of artificially splitting genomes into separate clusters is too high to warrant their use.

References
Barco, R., G. Garrity, J. Scott, J. Amend, K. Nealson, and D. Emerson. 2020. A genus definition for bacteria and archaea based on a standard genome relatedness index. mBio 11:e02475-02419.
Dvergedal, H., S. R. Sandve, I. Angell, G. Klemetsdal, and K. Rudi. 2020. Association of gut microbiota with metabolism in juvenile Atlantic salmon. Microbiome 8:160.
Konstantinidis, K. T., and J. M. Tiedje. 2005. Towards a genome-based taxonomy for prokaryotes. Journal of Bacteriology 187:6258-6264.
Pinna, N. K., R. M. Anjana, S. Saxena, A. Dutta, V. Gnanaprakash, G. Rameshkumar, S. Aswath, S. Raghavan, C. S. S. Rani, and V. Radha. 2021. Trans-ethnic gut microbial signatures of prediabetic subjects from India and Denmark. Genome Medicine 13:36.
Sanford, R. A., K. G. Lloyd, K. T. Konstantinidis, and F. E. Löffler. 2021. Microbial taxonomy run amok. Trends in Microbiology 29:394-404.
Schloss, P. D. 2021. Amplicon sequence variants artificially split bacterial genomes into separate clusters. mSphere Jul 21:e0019121. doi: 10.1128/mSphere.00191-21.
Schloss, P. D., and S. L. Westcott. 2011. Assessing and improving methods used in operational taxonomic unit-based approaches for 16S rRNA gene sequence analysis. Applied and Environmental Microbiology 77:3219-3226.
Wen, C., W. Yan, C. Mai, Z. Duan, J. Zheng, C. Sun, and N. Yang. 2021. Joint contributions of the gut microbiota and host genetics to feed efficiency in chickens. Microbiome 9:126.
Yarza, P., P. Yilmaz, E. Pruesse, F. O. Glöckner, W. Ludwig, K.-H. Schleifer, W. B. Whitman, J. Euzéby, R. Amann, and R. Rosselló-Móra. 2014. Uniting the classification of cultured and uncultured bacteria and archaea using 16S rRNA gene sequences. Nature Reviews Microbiology 12:635-645.

Line 157 - what are "singleton OTUs"? OTUs with only one read? OTUs found in only one sample?
Response: "singleton OTUs" represent OTUs found in only one sample (Lines 483-484).
"Singleton OTUs (OTUs found in only one sample) that did not match the reference database were removed."

Powered by Editorial Manager® and ProduXion Manager® from Aries Systems Corporation

Lines 159-161 - why were low abundance OTUs discarded? Does it affect your analysis? In general, it is better not to modify the data base extensively. Specifically, some phylogenetic groups are represented by multiple low abundant strains with a high total abundance whereas other phylogenetic groups are represented by a single highly abundant strain. By getting rid of such lower abundance OTUs you might be creating an artifact.
Response: Thank you for the helpful comments. Errors introduced by next-generation amplicon sequencing tend to induce spurious OTUs and spurious counts in OTU tables, both of which are especially prevalent at low abundances. Despite the power of NGS and the progress achieved, generated data is imperfect, being subject to different types of errors, including those inherent to PCR amplification (substitutions and chimeric sequence formation) and sequencing-specific biases which are characteristic of each sequencing technology. Sequencing errors are predominantly caused by base substitutions, although base deletions, low-quality reads, variable read lengths and non-target amplification are also known error sources which may result in false species affiliation. Undetected chimeric sequences, caused by the hybridization of DNA fragments from different species also reduce the reliability of the 16S rDNA sequence-based phylogenetic composition of microbial communities. Together, these different errors generate a high number of lower-abundance sequences, which lead to overestimations of actual community diversity and the creation of many false taxa. Those spurious sequences are hard to filter out using current mainstream pipelines implementing error correction, denoising, and stringent filtration of chimeric sequences, contaminants and non-bacterial contents. Previous study have reported that although the overall abundance of these pseudo sequences was low, introducing them into analysis increased the total number of features to 10 times higher than expected and enlarged the divergence of the alpha and beta diversity analyses among the different methods (Wang et al., 2018). Lower-abundance and lower-quality sequences were observed to surround higher-abundance, biologically real sequences, forming error clouds (Bokulich et al., 2013; Edgar, 2013). Various researchers have developed different approaches to remove these pseudo sequences.

In our work, we described the numerical distribution of OTUs with different existing ratios in samples, after which the slope fluctuation is estimated. We considered there are a lot of false positives for these microbiotas when fluctuations are great. Therefore, we selected OTUs that existed in more than 20% of samples according to the slope distribution curve (Supplementary Fig. S1a, b). This filtering rule will improve the stability and accuracy of further analyses. Similar filtering rules have also been reported in previous analyses (Zierer et al., 2018). In addition, we have cited Supplementary Fig. S1a, b in the revised manuscript (Lines 488-489).

References
Bokulich, N. A., S. Subramanian, J. J. Faith, D. Gevers, J. I. Gordon, R. Knight, D. A. Mills, and J. G. Caporaso. 2013. Quality-filtering vastly improves diversity estimates from Illumina amplicon sequencing. Nature Methods 10:57-59.
Edgar, R. C. 2013. UPARSE: highly accurate OTU sequences from microbial amplicon reads. Nature Methods 10:996-998.
Wang, J., Q. Zhang, G. Wu, C. Zhang, M. Zhang, and L. Zhao. 2018. Minimizing spurious features in 16S rRNA gene amplicon sequencing. PeerJ Preprints 6:e26872v1 https://doi.org/10.7287/peerj.preprints.26872v1
Zierer, J., M. A. Jackson, G. Kastenmüller, M. Mangino, T. Long, A. Telenti, R. P. Mohney, K. S. Small, J. T. Bell, and C. J. Steves. 2018. The fecal metabolome as a functional readout of the gut microbiome. Nature Genetics 50:790-795.

Lines 378-382 - this is an interesting idea that you did not establish well. Could you determine unifrac (or any other metric) distances of samples from different organs. i.e. compare all vagina samples to all small intestine samples and determine average Unifrac distances and stDev, and do so for each pair of organ sites. Perhaps you will gain a new figure showing how similar or different are the communities in each site.
Response: We are grateful for the suggestion. As suggested by the reviewer, we added a new figure (Supplementary Fig. S2j) showing the weighted UniFrac distances among the six sites.

Line 387 - cyanobacteria are not likely gut or reproductive tract inhabitants. This is more likely an artifact of identifying feed derived chloroplast DNA. You might want to mention this.

Powered by Editorial Manager® and ProduXion Manager® from Aries Systems Corporation

	Response: Thanks for your helpful suggestions. The chickens used in our study are fed using corn-soybean-based diets, so the Cyanobacteria (likely an artifact of feed-derived chloroplast DNA) were misidentified as microbes. Additionally, we have revised the sentence in the revised manuscript (Lines 118-120).<br>"Firmicutes, Proteobacteria, and Cyanobacteria (likely an artifact of feed-derived chloroplast DNA) accounted for 71.45% - 97.86% of all OTUs."<br><br>Lines 398-402 - what is the connection between the "well-documented protective role of lactobacillus…" and differences in the levels of lactobacillus between gut and reproductive tract? Does the reproductive tract not deserve protection? Please stick to the data. Do not attach unsupported conclusions. Instead connect the levels of Lactobacillus and pH.<br>Response: As suggested, we have connected the levels of Lactobacillus and pH in the revised manuscript (Lines 134-139).<br>"Lactobacillus is thought to inhibit pathogenic bacteria by lowering the environmental pH through lactic acid and hydrogen peroxide production [24]. This genus was highly abundant in the digestive tracts which were characterized by low pH values which strongly limits the growth of most pathogens [25, 26]. In contrast, Lactobacillus was less abundant in the reproductive tract where an alkaline pH is needed to maintain sperm motility [27, 28]."<br><br>Lines 407-411 - if I understand correctly, you are implying a connection possibly caused because of the flow of material from different sites. You may want to note that.<br>Response: Thank you for the helpful comments. Yes, we want to note that, and we have added a sentence in our revised manuscript (Lines 146-147).<br>"These results imply there is a connection of microbiome communities possibly caused because by the flow of material from different sites."<br><br>Line 419-421 - you have reduced a whole field of study to just one "previous study". And in any case I am not sure what you wanted to write here.<br>Response: We have now included additional references in the revised manuscript (Line 158).<br><br>Line 442 - not chloroplast and mitochondria-like microbes but rather true chloroplasts and mitochondria which you misidentified as microbes.<br>Response: Thanks for your helpful suggestion. We have revised the sentence in the manuscript (Lines 179-180).<br>"Unidentified Chloroplast (2.94%) and mitochondria-like (2.37%) materials from plant consumption."<br><br>Line 447 - does Helicobactor maintain near natural pH and a microaerophilic environment? Is that its role in the gut? Please do not bring up bits and pieces of information if this is not really relevant to your results. What is important is that a possible pathogen was found in your chickens.<br>Response: Thanks for the comments. In order to infect the gastric mucosa, Helicobacter pylori has to survive in the gastric acidic pH, and Helicobacter pylori has well developed mechanisms to neutralize the effects of acidic pH (Ansari, S. and Y. Yamaoka. 2017. Survival of Helicobacter pylori in gastric acidic territory, helicobacter 22:e12386), that is its role in the gastric tract. In addition, we have reorganized the sentence in the revised manuscript (Lines 183-185).<br>"As a possible pathogen, Helicobacter specifically inhabits the small intestine in chickens, and may be involved in inflammation, metabolism, and neutralization of gastric acid [35-37]."<br><br>Lines 450-463 - this whole paragraph contains a lot of disjoined bits and pieces of information. Does it really matter that some bacteria were changed in immune suppressed honey bees? What are you trying to say? Consider taking this whole paragraph off.<br>Response: Thanks for the comment. This whole paragraph mainly displays the results of the site-associated bacterial taxa in the three reproductive tract sites identified by LEfSe and discussed association with the previous literature. Additionally, we have deleted the disjoined bits and pieces of information and reorganized the sentence in the paragraph in our revised manuscript (Lines 186-194).<br>"Unidentified Erysipelotrichaceae showed higher abundance in the three reproductive

Powered by Editorial Manager® and ProduXion Manager® from Aries Systems Corporation

tract sites (1.83% - 2.40%). Bacteria associated with the isthmus and uterus both showed higher abundances than in the other sites. Several genera (typically, Romboutsia, Fusobacterium, and Clostridium sensu stricto 1) were dominant in the vagina (> 25% of the microbiota) but had lower abundances in the other sites (Fig. 2b). Among these, vaginal Romboutsia could be employed as a predictor for egg number in laying hens [8]. Six Bacteroidetes bacterial taxa were isthmus-associated; Bacteroides species live on host mucus-secreted polysaccharides and this flexible foraging behavior contributes to diversity and stability [38]."

Lines 464-466 - do your results support this speculation? If yes please expend, if not please remove.
Response: Thank you for the suggestion. Lines 464-466 have been removed from the revised manuscript.

Line 492 - 0.21% of total abundance? Of total species?
Response: Yes, it is "of total species". We have reorganized the sentence and clarified in the manuscript (Line 223).
"accounting for 0.21% of the microbiota species tested in the small intestine".

Lines 560-568 - this is a good example of discussion! Here you brought relevant information and created a data based speculation.
Response: Thank you for your positive remarks about this discussion.

Line 646 - what do you mean in "microbiome succession"?
Response: "microbiome succession" has been changed to "microbial community" in the revised manuscript (Line 390).

Supplementary figure 2 contains two separate topics - functional sequencing data and bacterial community analysis. Please divide into separate figures.
Response: As suggested, we have divided Supplementary figure 2 into two separate figures (Supplementary Fig. S2 and S11).

Reviewer #2: This is a very good study to which I have only a few comments, mostly to microbiota part in which I am stronger than in chicken genetics. The only weaker part, but not weak, is the fact that the study was performed with only a single flock and was not repeated with different hens at all. I understand that it is impossible to repeat the study in another flock but for example you could have set up Cl. leptum PCR and check for its presence in reproductive tract in completely different birds.
Response: We appreciate the positive feedback and sincerely thank the reviewer for the thoughtful and supportive recommendations, which are of great help in improving the quality of this manuscript.

Please consider the following points.
line 94, indeed laparotomy?
Response: "After laparotomy" has been changed to "After the abdomen was opened" in the revised manuscript (Line 417).

l.111 and 114, I do not understand wha PCR kit you used. Please, reword.
Response: The PCR amplification was performed using Phusion® High-Fidelity PCR Master Mix (New England Biolabs, Ipswich, Ma, USA). We have revised the sentence in the revised manuscript (Lines 436-439).
"The V4 hypervariable region of the bacterial 16S rDNA was amplified using Phusion® High-Fidelity PCR Master Mix (New England Biolabs, Ipswich, Ma, USA) and the universal primers 515 F (5′-GTGCCAGCMGCCGCGGTAA-3′) and 806 R (5′-GGACTACHVGGGTWTCTAAT -3′) [56]."

l.253, 254, this sentence is somewhat compromised. Please check and reword.
Response: Thank you for the comments, and we have revised the sentence in the revised manuscript (Lines 578-579).
"In addition, we further removed the SNPs with adjacent distances ≤ 5."

l.342, rather verified, or confirmed, then detected. By the way, I am not sure whether you used data from the qPCR in the rest of the manuscript
Response: We have changed "detected" to "verified" in the revised manuscript (Line

	664). We only used qPCR data in the "RNA sequencing (RNA-seq) analysis" section.<br><br>l.388, these are not Cyanobacteria, this is chloroplast DNA from plants in the feed, you may check very recent paper Volf et al. Eggshell and Feed Microbiota Do Not Represent Major Sources of Gut Anaerobes for Chickens in Commercial Production. Microorganisms 2021, 9, 1480.<br>Response: Thanks for the valuable suggestions, and we have read the above reference carefully. The chickens used in our study are fed using corn-soybean-based diets, so the Cyanobacteria (likely an artifact of feed derived-chloroplast DNA) was misidentified as microbes. Additionally, we have revised the sentence in the manuscript (Lines 121-122).<br>"Cyanobacteria (likely an artifact of feed-derived chloroplast DNA) was the dominant material in the gizzard (48.19% of the total abundance)."<br><br>l.462,463, I would suggest alternative explanation in this case and this is living on expense of host mucus secreted polysaccharides, Sonnenburg, J. L. et al. Glycan foraging in vivo by an intestine-adapted bacterial symbiont. Science 307, 1955-1959 (2005)<br>Response: As suggested, we have cited the reference and revised the sentence in the manuscript (Lines 192-194).<br>"Bacteroides species live on host mucus-secreted polysaccharides and this flexible foraging behavior contributes to diversity and stability [38]."<br><br>l.559, check that B. fragilis might be a consequence of intensive human care, Kollarcikova et al. Different Bacteroides Species Colonise Human and Chicken Intestinal Tract. Microorganisms 2020, 8, 1483.<br>Response: We are grateful for the suggestion. We have cited the useful and interesting reference in the revised manuscript (Lines 301-303).<br>"Interestingly, a recent study reported that the human-adapted Bacteroides species are likely introduced to chicken flocks by contact with humans and can temporarily persist in chickens [49]."<br><br>l.615, how can you know this? What if all of this the other way round, and I indeed believe that this is the other way, i.e. hens becomes of compromised performance, due to whatever factor, within but possibly also outside of those which you have monitored. This naturally results in decrease in egg lay but also in increased inflammatory response. Locally changed conditions due to inflammatory signaling change, infiltrating heterophils and macrophages produce antimicrobial peptides and reactive oxygen species and strict anaerobes will be the firs bacterial species to decrease in a response of increasing oxygen concentration. What is cause and what is consequence. I do not know, I think that you do not know either, though you blame bacteria that these are responsible for the response.<br>Response: Thank you for the comments. We deleted the sentence that caused misunderstanding.<br><br>l.631, similar to previous comment, I do not think that there is any downregulation. In high egg producers, there is basal, background expression of inflammatory marker genes. And these are induced in the hens with compromised performance. Be also careful, whether this induction since this could also be a cause of infiltration of macrophage with their specific expression profile, and you then mistakenly conclude on induction when purifying mRNA from a total complex tissue.<br>Response: Thanks for your comments. "downregulated" has been changed to "different expressed" in the revised manuscript (Line 383).<br><br>l.656, the same as above, be careful what is cause and what is consequence. Increase in reactive oxygen species may affect the most strict anaerobes. When these are present, there is no inflammation. When these are eliminated by increase in oxygen concentration and all other inflammatory responses, this is explained that these bacteria are anti-inflammatory. These are not, these only dislike inflammation and oxygen.<br>Response: Thanks for your helpful suggestion. We have deleted the sentence in the revised manuscript.
**Additional Information:**	

Powered by Editorial Manager® and ProduXion Manager® from Aries Systems Corporation

Question	Response
Are you submitting this manuscript to a special series or article collection?	No
**Experimental design and statistics**<br><br>Full details of the experimental design and statistical methods used should be given in the Methods section, as detailed in our Minimum Standards Reporting Checklist. Information essential to interpreting the data presented should be made available in the figure legends.<br><br>Have you included all the information requested in your manuscript?	Yes
**Resources**<br><br>A description of all resources used, including antibodies, cell lines, animals and software tools, with enough information to allow them to be uniquely identified, should be included in the Methods section. Authors are strongly encouraged to cite Research Resource Identifiers (RRIDs) for antibodies, model organisms and tools, where possible.<br><br>Have you included the information requested as detailed in our Minimum Standards Reporting Checklist?	Yes
**Availability of data and materials**<br><br>All datasets and code on which the conclusions of the paper rely must be either included in your submission or deposited in publicly available repositories (where available and ethically appropriate), referencing such data using a unique identifier in the references and in the "Availability of Data and Materials" section of your manuscript.	Yes

Have you have met the above requirement as detailed in our Minimum Standards Reporting Checklist?	

Manuscript w. DOI
Click here to access/download;Manuscript;Revised Manuscript DOI.docx

Female reproductive tract microbiota influence egg production in layer chickens

Yuan Su[1,†] , Shilin Tian[2,3,†], Diyan Li[1,*,†], Wei Zhu[1,†], Tao Wang[1,†], Shailendra Kumar Mishra[1], Ranlei Wei[4], Zhongxian Xu[1], Mengnan He[1], Xiaoling Zhao[1], Huadong Yin[1], Xiaolan Fan[1], Bo Zeng[1], Mingyao Yang[1], Deying Yang[1], Qingyong Ni[1], Yan Li[1], Mingwang Zhang[1], Qing Zhu[1,*], Mingzhou Li[1,*]

[1]Farm Animal Genetic Resources Exploration and Innovation Key Laboratory of Sichuan Province, Sichuan Agricultural University, Chengdu 611130, China.

[2]Department of Ecology, Hubei Key Laboratory of Cell Homeostasis, College of Life Sciences, Wuhan University, Wuhan 430072, China.

[3]Novogene Bioinformatics Institute, Beijing 100000, China.

[4]Center of Precision Medicine, West China Hospital, Sichuan University, Chengdu 610065, China.

[†]These authors contributed equally to this paper.

[*]Corresponding author. e-mail: Diyan Li: diyanli@sicau.edu.cn; Qing Zhu: zhuqing@sicau.edu.cn; Mingzhou Li: mingzhou.li@sicau.edu.cn

Abstract

Background: The microbiota of the female reproductive tract is increasingly recognized as having fundamental roles in animal reproduction. To explore the relative contribution of reproductive tract microbiomes on egg production in chickens, we investigated the microbiota in multiple reproductive and digestive tract sites from 128 female layer (egg-producing) chickens in comparable environments.

Results: We identified substantial differences between the diversity, composition, and predicted function of site-associated microbiota. Reproductive tract microbiota were more profoundly affected egg production than that in the digestive tract. We identified four reproductive tract microbial species, *Bacteroides fragilis, B. salanitronis, B. barnesiae,* and *Clostridium leptum*, which were related to immune function and potentially contribute to enhanced egg production.

Conclusions: These findings provide insights into the diverse microbiota characteristics of reproductive and digestive tracts, and may help design strategies for controlling and manipulating chicken reproductive tract microbiota to improve egg production.

Keywords: microbiota, reproductive tract, egg production, chicken

Background

The domestic chicken (*Gallus gallus domesticus*) is of enormous agricultural significance, comprising broiler (meat-producing) and layer (egg-producing) chickens. Specialized commercial layer breeds were established during the twentieth century with greatly improved reproductive traits [1]. Currently, thousands of quantitative trait loci (QTLs) [2] and many gene mutations [3, 4] are reportedly associated with chicken

reproductive traits. Nonetheless, egg production, as a polygenic inheritance trait, exhibits low to moderate heritability (h^2, ranging from 0.05 to 0.44, depending on the period involved) [5, 6]. Alternative effective approaches for modulating egg production in laying hens are urgently required for the poultry industry to meet consumer demand.

Distinct bacterial communities throughout the female reproductive tract form a microbiota continuum from the vagina to the isthmus, which has a prominent role in animal reproduction [7, 8]. In humans, microbiome interactions with the host during pregnancy leading to preterm birth were investigated [9], and temporal changes in the vaginal microbiome associated with full-term pregnancies were identified [10]. An abnormal vaginal microbiota may predispose individuals to increased microbial invasion of the amniotic cavity and fetal damage [11, 12]. The avian reproductive tract houses complex bacterial communities that are believed to play crucial roles in egg production [13]. Chicken digestive and reproductive tracts are mainly colonized by *Firmicutes*, *Bacteroidetes*, *Proteobacteria*, *Actinobacteria*, and *Fusobacteria*, which are spatially organized within specific digestive and reproductive compartments [14, 15]. Additionally, *Lactobacillus* species were found to be keystone species residing in the chicken oviduct [16].

Several synergistic factors, such as environment and diet, dominate over host genetics in determining gut microbiota composition [17, 18]. A comparative study of gut microbial diversity among parrot species indicated the potential role of host ancestry in shaping the gut microbiome [19]. A genome-wide association study (GWAS) in chickens demonstrated the genetic loci rs15142709 and rs15142674 which are located in the pleiomorphic adenoma gene 1 (*PLAG1*) and lck/yes-related novel tyrosine kinase (*LYN*) genes were significantly associated with microbial *Methanobacterium* abundance [20]. In a previous study, 14 identified QTLs strongly

influenced *Clostridium leptum* and *Lactobacillus* abundance, as well as related candidate genes involved in anti-inflammatory responses and the motility of the digestive tract [21]. On the other hand, recent studies have suggested that host genetics have limited impact on gut microbiota composition in humans [22].

We speculated that the microbial component of the reproductive tract might be an important aspect of egg production in chicken. Here, we performed 16S rDNA sequencing on 768 samples from three reproductive (vagina, uterus, and isthmus) and three digestive (crop, gizzard, and small intestine) tract sites and whole-genome sequencing of 128 laying hens. We characterized the reproductive tract microbiota and its features compared with those of the digestive tract microbiota of hens. We identified the contribution of key microorganisms to egg production, and established a correlation between host genetics and the microbial diversity of six tract sites. These findings provide insights into the microbial communities in the reproductive tract of highly specialized layer populations, which may help develop strategies to enhance commercial egg production.

Results and Discussion

Discriminative characteristics of microbiota in reproductive and digestive tract sites

The 16S rDNA sequencing in 768 samples generated a total of ~57.61 M high-quality reads (~75.01 K reads per sample). *De novo* clustering after singleton removal produced 46,480 operational taxonomic units (OTUs) at an identity cutoff of 97%, among which 6,776 OTUs found in > 20% of samples were used for subsequent analysis (**Supplementary Fig. S1a, b**). We performed alpha diversity analysis based on

qualified sequencing depth with an average Good's coverage of 98.69% (ranging from 96.30% - 99.60%) (**Supplementary Fig. S2a**). Analysis of five indices (i.e., observed OTUs, ACE, Chao1, Simpson, and Shannon) (**Supplementary Fig. S2b-f**) indicated that the vast majority of pairwise comparisons between sites (10 of 90 pairwise comparisons, or 11.11%) showed significant differences ($P < 0.001$, Wilcoxon rank-sum test), with the exception of two comparisons (uterus vs. isthmus for all indices; and small intestine vs. vagina for the observed OTUs, Simpson, and Shannon indices) (**Supplementary Fig. S2g, h**).

Compared to the digestive system, the reproductive system exhibited higher alpha diversity (all five indices) and thus contained more microbial taxa, especially in the upper reproductive tract (i.e., uterus and isthmus) (**Fig. 1a, b**). Similar to significant microbiota differences between the vaginal and upper reproductive tracts in humans [7, 23], we found highly discriminative microbial communities in chickens between the upper (isthmus and uterus) and lower (vagina) reproductive tracts (isthmus vs. vagina, $R = 0.473$, $P < 0.001$, Analysis of Similarity [ANOSIM]; uterus vs. vagina, $R = 0.496$, $P < 0.001$, ANOSIM), but indistinguishable microbiota between the isthmus and uterus (isthmus vs. uterus, $R = -0.003$, $P = 0.694$, ANOSIM) (**Supplementary Table S1**). These results demonstrated microbiota heterogeneity throughout contiguous sites of the digestive and reproductive tracts in hens.

We used principal coordinates analysis (PCoA) to visualize differences in taxa composition between microbiota in the reproductive and digestive tracts. The first principal component, explaining 34.96% of the variance in weighted UniFrac distance matrices among the samples, separated reproductive and digestive tract samples (**Fig. 1c**). Given that reproductive and digestive tracts share a common exit in the cloaca, the frequently exchanged microbiome likely resulted in similar microbiota at the distal end

of both tracts. Consequently, we found that the vagina acquired microbe communities from the isthmus and uterus, which all belong to the reproductive tract. Nonetheless, the vagina microbiota was partially indistinguishable from that of the small intestine (**Fig. 1c**). The unweighted UniFrac distance matrices (**Supplementary Fig. S2i**), weighted UniFrac distance (**Supplementary Fig. S2j**) and an ANOSIM based on Bray-Curtis (BC) distances (**Supplementary Table S1**) recapitulated these findings.

Similar phyla dominated the microbiota in the six sites; *Firmicutes*, *Proteobacteria*, and *Cyanobacteria* (likely an artifact of feed-derived chloroplast DNA) accounted for 71.45% - 97.86% of all OTUs. Nonetheless, some differences were observed among the sites. *Cyanobacteria* (likely an artifact of feed-derived chloroplast DNA) was the dominant material in the gizzard (48.19% of the total abundance); however, *Firmicutes* was the most abundant phylum (43.60% to 78.93%) in the other five sites. We also found that the uterus and isthmus had similar dominant phyla, including *Firmicutes* (44.87% and 43.60%), *Proteobacteria* (26.25% and 23.77%), and *Bacteroidetes* (17.13% and 19.52%) (**Supplementary Fig. S2k**). Strikingly, the vagina had the highest abundance of *Fusobacteria* (11.51%) among the six sites.

At the genus level, *Lactobacillus* (7.24% - 73.74% in the six sites), *Exiguobacterium* (2.79% - 4.68%), *Stenotrophomonas* (1.01% - 6.54%), and *Bacteroides* (3.40% - 4.86%) were ubiquitously found across all sites with higher abundances than other bacteria due to their broad adaptability and beneficial functions (**Fig. 1a**). We found *Lactobacillus* to be more dominant in the digestive tract (73.74% in crop, 24.76% in gizzard, 30.70% in small intestine) compared with the reproductive tract (7.24% in vagina, 9.27% in uterus, 9.91% in isthmus). *Lactobacillus* is thought to inhibit pathogenic bacteria by lowering the environmental pH through lactic acid and hydrogen peroxide production [24]. This genus was highly abundant in the digestive tracts which

were characterized by low pH values which strongly limits the growth of most pathogens [25, 26]. In contrast, *Lactobacillus* was less abundant in the reproductive tract where an alkaline pH is needed to maintain sperm motility [27, 28]. *Unidentified Erysipelotrichaceae, Unidentified Chloroplast, Lactobacillus,* and *Bacteroides* had abundances of >1.0% in the vagina, which was further increased in the uterus and isthmus (**Fig. 1a**).

Furthermore, 14.63% of genera (362 of 2,475) demonstrated associations between sites ($P < 0.05$ of Spearman’ s r, Z-test) (**Supplementary Fig. S2l**). Typically, genera belonging to *Proteobacteria* and *Firmicutes* showed significantly positive correlations ($P < 0.001$, Z-test) between the crop and gizzard, the gizzard and small intestine, or the three reproductive tract sites. These results imply there is a connection of microbiome communities possibly caused because by the flow of material from different sites.

Site-associated microorganisms in reproductive and digestive tracts

We analyzed the functional capacity of the microbiota in each reproductive and digestive tract site using PICRUSt2, and found that 72.00% of the representative pathways (36 of the top 50 Kyoto Encyclopedia of Genes and Genomes [KEGG] pathways) were shared across the six sites, one third of which (12 of 36) were primarily involved in metabolism (**Supplementary Fig. S3a, b**). Specifically, ‘bacterial secretion system’ and ‘bacterial chemotaxis’ were enriched in the reproductive tract. Previous studies found that successful bacterial pathogens evolved versatile protein secretion systems to promote their survival and fitness in response to different environmental challenges, and to modulate host immunity [29-32]. Seven pathways were specifically enriched at a site (three of six were site-specific to crop and gizzard, and ‘riboflavin

metabolism' was specific to vagina). Abundances of the OTUs involved in these pathways differed among the six sites ($P < 0.001$, Wilcoxon rank-sum test) (**Supplementary Table S2**). For example, the microbial community of the small intestine had important roles in 'valine, leucine, and isoleucine biosynthesis', as indicated by the moderate row *Z* scores (-0.66) for each pathway. Moreover, 'propanoate metabolism' (*Z* score = 1.72) and 'bacterial chemotaxis' (*Z* score = 1.53) were overrepresented in the vagina. Meanwhile, 'bacterial secretion system' was overrepresented only in the uterus (*Z* score = 1.29) and isthmus (*Z* score = 0.94) compared with the vagina and the three digestive tract sites (**Fig. 2a**).

We next identified 65 site-associated bacterial taxa among the six sites using Linear discriminant analysis (LDA) Effect Size (LEfSe); [33] (**Fig. 2b and Supplementary Fig. S3c**), which confirmed most of the observations described above (i.e., the uterus and isthmus showed essentially similar microbiota). Of note, *Helicobacter* and *Unidentified Erysipelotrichaceae*, which were associated with the small intestine, showed the highest abundance among the six sites (**Fig. 2b**). Six genera from *Lactobacillaceae* were crop-associated bacteria. In chicken, the crop acts as a reservoir for the storage of food prior to its digestion, where food mixes with many beneficial *Lactobacillus* bacteria (73.84% at the genus level) that produce lactic acid before moving on to the proventriculus [34]. Next, the gizzard grinds any remaining large food particles with the assistance of grit, releasing abundant *Unidentified Chloroplast* (2.94%) and mitochondria-like (2.37%) materials from plant consumption. The small intestine exhibited the most abundant microbes of the three digestive tract sites, which is mainly where further digestion occurs, and fermentation begins. *Paenibacillaceae* species, with optimum growth at pH 6.0-7.0, were also overrepresented. As a possible pathogen, *Helicobacter* specifically inhabits the small intestine in chickens, and may

be involved in inflammation, metabolism, and neutralization of gastric acid [35-37].

Unidentified Erysipelotrichaceae showed higher abundance in the three reproductive tract sites (1.83% - 2.40%). Bacteria associated with the isthmus and uterus both showed higher abundances than in the other sites. Several genera (typically, *Romboutsia*, *Fusobacterium*, and *Clostridium sensu stricto 1*) were dominant in the vagina (> 25% of the microbiota) but had lower abundances in the other sites (**Fig. 2b**). Among these, vaginal *Romboutsia* could be employed as a predictor for egg number in laying hens [8]. Six *Bacteroidetes* bacterial taxa were isthmus-associated; *Bacteroides* species live on host mucus-secreted polysaccharides and this flexible foraging behavior contributes to diversity and stability [38].

These findings confirm that the digestive and reproductive tract microbiotas in chicken are primarily determined by the physiological function of each compartment within these systems.

Weak association between host genetics and microbial communities

In order to explore the relationship between host genome and microbiome of six sites, we generated a total of 1.76 tera bases of high-quality genome sequences from 128 chickens with ~10.15-fold average depth for each individual (**Supplementary Table S3**), and identified a total of 10.82 M single nucleotide polymorphisms (SNPs) with a density of ~10.29 SNPs per kb.

The correlation between host genetics (using genetic relatedness matrix [GRM] and microbial beta diversity based on BC distance) at the six sites in the same cohort of laying hens was not statistically significant ($|r| < 0.033$, $P > 0.05$, Mantel test, **Fig. 3a-f and Supplementary Table S4**). Nonetheless, the microbiomes of anatomically

neighboring sites were similar. Typically, the microbial communities of the isthmus were positively correlated with those of the neighboring uterus (Spearman's $r = 0.426$, $P < 0.0001$, Mantel test), but not significantly associated with the relatively distant crop (Spearman's $r = 0.019$, $P = 0.335$, Mantel test, **Supplementary Table S4**). We also estimated the association between GRM and microbial relationship matrix (MRM), and obtained similar results: both Pearson's and Spearman's correlations suggesting that host genetics and the microbiota composition are weakly associated (**Supplementary Table S4**).

We next regarded the abundance of each microorganism as a quantitative trait to estimate the h^2 of each microorganism at the species, genus, and OTU level. Microorganisms in > 20% but < 60% of samples were analyzed qualitatively as dichotomous traits (**Fig. 3g-i**). At the species and genus levels, no significant correlation ($P > 0.05$, Wilcoxon rank-sum test) was found between the presence of a SNP and the presence of a specific microbe (**Fig. 3j-l**) in the crop. Three species in the small intestine (accounting for 0.21% of the microbiota species tested in the small intestine), six in the vagina (0.39%), 14 in the uterus (0.92%), and ten in the isthmus (0.66%) exhibited significant SNP-based heritability ($P < 0.05$, Wilcoxon rank-sum test) (**Fig. 3k and Supplementary Table S5**). Most of these heritable bacteria belonged to the *Firmicutes* phylum (**Supplementary Fig. S4a, c, e**). Reproductive tract sites had more heritable bacterial phyla than digestive tract sites (**Supplementary Fig. S4b, d, f**). The cumulative abundances of these heritable bacteria were only 0.22%, 4.14%, 1.46%, and 1.61% ($P < 0.05$, Wilcoxon rank-sum test) in the small intestine, vagina, uterus, and isthmus, respectively (**Fig. 3n and Supplementary Table S5**). Similar results were observed at the genus and OTU levels (**Fig. 3m, o**). These results supported that host genetics have limited effect on shaping the microbial composition of the reproductive

and digestive tracts.

Heritability (h^2) and microbiability (m^2) of EN300

To further explore the effect of genome and microbiome on egg number at 300 days of age (EN300), we used a GRM of sample pairs to estimate the h^2 value of EN300 explained by whole genome SNPs using the restricted maximum likelihood method. We found that EN300 exhibited relatively low to medium heritability (h^2 = 0.282, P = 0.048, likelihood ratio test), which was comparable to previous estimations (**Supplementary Table S6**) [5, 6]. The fraction of EN300 variance explained by microbial variance was measured by microbiability (m^2) [18]. After correcting for host genetic factors using EN300-related SNPs as additional covariates, we found that the estimated EN300 m^2 values for digestive tract sites (0.523 for small intestine, 0.869 for crop, and 0.873 for gizzard) were lower than those for reproductive tract sites (0.923 for vagina, 0.936 for uterus, and 0.989 for isthmus) (**Table 1**). Generally, higher EN300 m^2 values were observed for sites neighboring the ovaries; the isthmus was the most pertinent site with respect to egg production. Commercial egg producers are acutely interested in hen oviducts because pathological changes or disrupted activity directly affect egg production efficiency and ultimately decrease economic profitability [39]. In chickens, the inner and outer shell membranes form in the isthmus, while calcification of the eggshell, subsequent pigmentation, and cuticle deposition occur in the uterus and is followed by expulsion of the egg through the vagina [40]. These results suggest that EN300 in layer chickens is determined more by the microbiota in the reproductive tract than in the digestive tract.

Table 1. Estimated microbiability (m^2) of EN300

	Site	m^2	Standard error	*P* value
**Digestive tract**	Crop	0.869	0.049	$< 10^{-16}$
	Gizzard	0.873	0.045	$< 10^{-16}$
	Small intestine	0.523	0.111	2.56×10^{-11}
**Reproductive tract**	Isthmus	0.989	0.011	$< 10^{-16}$
	Uterus	0.936	0.030	$< 10^{-16}$
	Vagina	0.923	0.028	$< 10^{-16}$
**Host genetics**		0.282	0.231	0.049

Microorganisms in the reproductive tract are significantly associated with EN300

We next focused on the microorganisms which are highly associated with EN300. The results showed that most of the microorganisms detected at the microbial species, genus, and OTU levels that significantly associated with EN300 belonged to the *Firmicutes* phylum ($P < 0.05$, Wilcoxon rank-sum test) (**Supplementary Fig. S5**). Only microorganisms that exhibited a significant correlation between egg production and relative abundance as determined by both Pearson's *r* and Spearman's *r* were considered a causal relationship ($P < 0.05$, Wilcoxon rank-sum test) Consequently, 39 OTUs, 26 genera, and 24 species fulfilled these criteria (**Fig. 4a and Supplementary Fig. S6a**).

Most OTUs, genera, and species present in the three digestive tract sites were negatively correlated (Pearson's *r*) with egg production (negative/positive: 19/6, 8/8 and 16/5, respectively), whereas they were positively correlated with egg production in the reproductive tract sites (positive/negative: 13/9, 11/10 and 13/4, respectively) (**Fig. 4b and Supplementary Fig. S6b, c**). Microorganisms in the uterus were most strongly correlated with each other (**Fig. 4c and Supplementary Fig. S7**), which implied a strong symbiotic/competitive relationship.

At the genus level, *Lactobacillus*, *Bacteroides*, and *Desulfovibrio* were positively

correlated with EN300 in the three reproductive tract sites. *Pseudomonas*, *Exiguobacterium*, and *Unidentified Erysipelotrichaceae* were negatively correlated with EN300 in the three reproductive tract sites, but were positively correlated in the three digestive tract sites (**Supplementary Fig. S6b**). In accordance with previous findings, there is clear evidence of the role of fecal microbiomes in low and high egg-laying performance in hens; Elokil et al [16] demonstrated a significantly positive association between the microbial genus *Lactobacillus* and egg-laying performance ($P < 0.05$). Likewise, Wang et al [41] reported that *Lactobacillus* was also abundant in the feces of high-yield hens. The genus *Lactobacillus* produces growth promoters and exhibits antimicrobial activity against pathogenic microbes [42, 43] which may explain why the increasing abundance of *Lactobacillus* in the high-yield group is beneficial to egg-laying performance. The cecum has distinct microbial community profiles [44-46] that were not explored in this study. Microbial community analysis of the cecum microbiota in chickens exhibiting different egg production performances requires further investigation.

At the species level, *Bacteroides fragilis*, *B. salanitronis*, *B. barnesiae*, and *C. leptum* were positively correlated with EN300 in the three reproductive tract sites, but were weakly negatively correlated with EN300 in the small intestine, while no correlation was found in the crop and gizzard. The first three species belong to the genus *Bacteroides*, which had a significantly positive correlation with egg production in the three reproductive sites (Pearson's r = 0.403 - 0.479). *Bacteroides* species have been identified as the predominant anaerobic genera in chicken cecum [47], which were thought to play an important role in the breakdown of polysaccharides into simpler compounds used by the animal host as well as the microorganisms themselves [48]. Interestingly, a recent study reported that the human-adapted *Bacteroides* species are

likely introduced to chicken flocks by contact with humans and can temporarily persist in chickens [49]. Intestinal anaerobic bacteria such as *B. fragilis* and *B. salanitronis* have been suggested to possess metabolic pathways for N-glycan production [50]. The symbiont *B. fragilis* exists in a commensal relationship with the host as it expresses a relatively large number of genes involved in polysaccharide metabolism, which benefits the host. The surface of *B. fragilis* can produce polysaccharides; in particular, capsular polysaccharide A (CPSA) is a key mediator of mammalian immune system development [51]. Surprisingly, CPSA has also been shown to exert protective effects in autoimmune disorder models, such as antibiotic-induced experimental encephalomyelitis. It is thus suggested that the genus *Bacteroides* could regulate reproductive activity by mediating the avian immune system.

Firmicutes bacterium ZOR0006 had a significantly negative correlation with EN300 in the three reproductive tract sites and a significantly positive correlation in the three digestive tract sites. The 20% of chickens with the lowest EN300 values (mean = 37.13) had significantly lower *B. fragilis*, *B. salanitronis*, *B. barnesiae*, and *C. leptum* abundances (P < 0.05, Wilcoxon rank-sum test) (**Supplementary Fig. S8a, b**) compared with the highest EN300 values (mean = 113.75) of the 20% of chickens in the reproductive tract sites. Although its function is unknown, 20% of chickens with the highest abundance of *Firmicutes* bacterium ZOR0006 exhibited significantly lower EN300 values than the 20% of chickens with the lowest abundance of this microorganism (**Fig. 5a**) (P < 0.05, Wilcoxon rank-sum test) in the reproductive tract sites.

Moreover, the 20% of chickens with the highest *B. fragilis*, *B. salanitronis*, *B. barnesiae*, and *C. leptum* abundances exhibited significantly higher EN300 values than the 20% of chickens with the lowest abundances of these microorganisms in the

reproductive tract sites, with the exception of *C. leptum* abundance in the vagina (**Fig. 5a**). *C. leptum*, a major member of the *Firmicutes* phylum, can alter the gut microbiota in rats, especially in obese individuals. In human infants, fecal levels of *C. leptum* were found to be negatively correlated with proinflammatory marker levels [52]. Colonic colonization of *C. leptum* was associated with accumulation of regulatory T cells, which inhibited the development of inflammatory lesions. The proliferation and activation of regulatory T cells is crucial to establishing and maintaining an appropriate level of immune tolerance. In addition, our results demonstrated that *C. leptum* was associated with a large range of other uterus or isthmus microbiota constituents (but limited association was observed with digestive microbiota constituents) and was not influenced by host genetics. Thus, this microorganism might serves as a stimulator of regulatory T cell production and inhibitor of inflammatory lesions, then regulating and maintaining immunologic tolerance and microbiota composition of the reproductive tract (especially the uterus and isthmus). These results suggest that the microbial species contribute to the enhanced egg production are modulated by influencing the immune processes.

We then characterized the spatial distribution of these five EN300-associated microorganisms (*B. fragilis*, *B. salanitronis*, *B. barnesiae*, *C. leptum*, and *Firmicutes* bacterium ZOR0006). *B. fragilis* was detected in almost all samples and accounted for 0.05% - 1.29% of the total abundance (**Fig. 5b**). *B. salanitronis* and *B. barnesiae* were detected at similar ratios in the six sites and in most samples from the reproductive tract sites; both accounted for the highest abundance in the vagina. *Firmicutes* bacterium ZOR0006 was also detected in most samples from the reproductive tract sites (74.22% - 89.84%) and in half of the samples from the digestive tract sites (48.44% - 58.59%), accounting for 0.61% - 2.40% of the total abundance. Although the detection ratio

(28.13% - 64.84%) and relative abundance of *C. leptum* were much lower than those of other microorganisms in all six sites, they accounted for the highest abundance in the isthmus and uterus (**Fig. 5b**).

Multiple factors, especially host species, potential pathogens, and immune status of the host, all play a major role in the female reproductive organs adversely interfering with the egg industry in laying flocks [53]. Additionally, the digestive tract environment of low-egg producing hens is fragile and susceptible to the influence of exogenous microorganisms [41]. Pathogenic infection, room temperature fluctuations, management systems, and other sudden changes to various factors can alter the composition of microbiota [54, 55]. These alterations may cause a significant degradation in production performance. Here, our results indicate that the reproductive tract microbiota play an important role in egg production.

Transcriptomic divergence in the uterus between hens with high and low egg production

Furthermore, we compared the transcriptional profiles in the uterus between the two groups composed of hens with either the 20% lowest or 20% highest EN300 values (six hens for each group). As expected, the correlation rates between the high- and low-egg production groups (mean Pearson's r = 0.93) were relatively lower than those between biological replicates (mean Pearson's r = 0.95 and 0.96 for groups with high and low egg production, respectively) (**Fig. 6a, b**), indicating significant biological differences between groups. We identified 1,051 genes that exhibited significant expression changes (false discovery rate [FDR] $\leq$ 0.01 and |log2 (fold change)| $\geq$ 1) between groups with high and low egg production (**Fig. 6c**), which are mainly involved in immune-

related categories, including the ‘NF−κB signaling pathway’ and ‘chronic inflammatory response’ (**Supplementary Fig. S9**). Of these, 739 genes that were significantly downregulated in the high-egg production group were overrepresented in the categories related to the inflammatory response, including ‘T cell costimulation’, ‘B cell receptor signaling pathway’ and ‘lymphocyte activation’ (**Fig. 6e**). Notably, we observed eight well-documented inflammatory markers (two Toll-like receptors [*TLR15* and *TLR1A*] and six interleukins [*IL21R*, *IL18RAP*, *IL22RA2*, *IL4I1*, *IL17REL* and *IL8*]) (**Fig. 6c, d**) that were significantly different expressed in the uterus of the high-egg production group and the low-egg production group. Functionally, the microbiota of the uterus affects the health of the oviduct and thus influence chicken egg production, which is manifested as increased pathway abundance for bacterial motility proteins, the bacterial secretion system, and membrane and intracellular structural molecules.

Conclusion

Our study provides a comprehensive view of the microbial community in the digestive and reproductive tracts of layer chickens. The diversity, composition, and predicted function of the microbiota varied considerably according to location within the reproductive and digestive tracts. Our results indicate that the reproductive tract microbiota in the hen influences egg production more than the digestive tract microbiota, and host genome has limited effect on their microbial composition. A small proportion of the variability in egg production was associated with the microbiota in the reproductive and digestive tracts of chickens. Remarkably, the genus *Bacteroides* and the species *C. leptum* and *Firmicutes* bacterium ZOR0006 were strongly associated with egg production, indicating their potential role in promoting reproductive performance. These findings provide new insight into the roles of reproductive and

digestive tract microbiota for complex traits, and may help contribute to the development of effective therapies for improving commercial egg production in chickens.

Methods

Chickens

The study was conducted on a common flock of 128 Dongxiang green shell laying hens reared on an experimental poultry farm at Sichuan Agricultural University in Ya'an, Sichuan, China. All chicks were hatched on the same day and housed in individual pens. Feed intake was controlled daily according to standard farm husbandry practices and water was provided *ad libitum*. The number of eggs produced for the first 300 days of life was recorded daily for each individual. We determined that the mean number of eggs (~75.32; ranging from 24-129) that each hen laid by 300 days of age (EN300) fit a normal distribution pattern (P = 0.725, Kolmogorov-Smirnov test) (**Supplementary Fig. S10**). At the age of 300 days, 2 mL of whole blood was collected from the wing vein using venipuncture and stored at -20 °C. Subsequently, each individual was culled by cervical dislocation followed by decapitation. After the abdomen was opened, fresh tissue was collected from three sites in the reproductive tract (vagina, uterus and isthmus) and three sites in the digestive tract (crop, gizzard and small intestine) (**Fig. 1a**). A 12-cm-long fixed mid-region of the small intestine (jejunum) was collected from each bird. All samples were snap-frozen in liquid nitrogen, transported to the laboratory, and stored at -80 °C until further analysis. Chickens were managed according to the Institutional Animal Care and Use Committee of Sichuan Agricultural University under permit number DKY- 2018102015.

Host and microbial genomic DNA extraction

Host DNA was isolated from blood using a TIANamp Genomic DNA Kit (Tiangen Biotech) following the manufacturer's instructions. Total microbial genomic DNA from lumen of digestive tracts and mucus of reproductive tracts were extracted from ~200 mg tissues using a TIANamp Stool DNA Kit (Tiangen Biotech, Beijing, China) according to the manufacturer's instructions. The extracted DNA was quantified using a NanoDrop2000 spectrophotometer (Thermo Fisher Scientific, Chengdu, China), and DNA integrity was determined by 1% agarose gel electrophoresis.

16S rDNA amplicon and sequencing

The V4 hypervariable region of the bacterial 16S rDNA was amplified using Phusion® High-Fidelity PCR Master Mix (New England Biolabs, Ipswich, Ma, USA) and the universal primers 515 F (5′-GTGCCAGCMGCCGCGGTAA-3′) and 806 R (5′-GGACTACHVGGGTWTCTAAT -3′) [56]. Reactions were carried out using 15 μL of Phusion® High-Fidelity PCR Master Mix, 3 μL of the forward and reverse primers, 10 μL of template DNA, and 2 μL of ddH_2O, no template control was also performed. The PCR cycling conditions were as follows: an initial denaturation step at 98 °C for 1 min and 30 cycles of 98 °C for 10 sec, 50 °C for 30 sec, and 72 °C for 30 sec, followed by an extension step at 72 °C for 5 min, using a Bio-Rad CFX96 thermal cycler (Bio-Rad Laboratories, Hercules, CA, USA). Amplicons were purified on agarose gel (1%) using a GeneJET Gel Extraction kit (Thermo Fisher Scientific, Schwerte, Germany). A DNA library was prepared using an Ion Plus Fragment Library Kit (Thermo Fisher Scientific) based on the manufacturer's instructions. Reads were barcoded per sample, combined

for multiplexed sequencing with the Ion S5^{TM} XL platform (Thermo Fisher Scientific) to generate 400-bp single-end reads, and sequenced by Novogene Bioinformatics Technology Co. Ltd of China.

Whole-genome sequencing

After qualified host DNA samples were tested, the DNA was randomly fragmented using an ultrasonicator (Covaris Inc., Woburn, MA, USA), and then a sequencing library was prepared using a TruSeq Nano DNA HT Sample Preparation Kit (Illumina, San Diego, CA, USA) following the manufacturer's instructions. Index codes were added to tag each sample. DNA fragments were then end-repaired, dA-tailed, and ligated with a full-length adapter for Illumina sequencing, PCR amplification, and purification. Next, isolated DNA libraries were constructed with an insert size of 350 bp. Finally, genomes of the 128 individuals were separately sequenced with 150 bp paired-end reads using the Illumina Novaseq platform by Novogene Bioinformatics Technology Co. Ltd.

16S rDNA-seq data processing

The raw data obtained by sequencing were first filtered to obtain high-quality data. First, the adapter sequences in 61.05 million (M) raw reads (**Supplementary Table S7**) were trimmed using Cutadapt (version 1.9.1) [57] when the overlap length between the read and the adapter was shorter than 10 bp. Then, barcode sequences were trimmed and reads that were too long (> 260 bp) or too short (< 220 bp) were filtered using Cutadapt with the parameters "-e 0 -q 17 -m 200 -M 2600". Next, the remaining reads were compared with the ChimeraSlayer reference database using the UCHIME algorithm [58]

to detect and then remove chimera sequences. Finally, low-quality reads (i.e., more than 50% of bases with a phred quality lower than five) were also removed. Consequently, 57.61 M high-quality reads were generated for subsequent analysis (**Supplementary Table S8**). Greater than 93.85% of the high-quality reads had lengths of 250 - 260 nt (**Supplementary Fig. S11a**). Data with a quality score > 20 accounted for 88.14% of all the effective bases (**Supplementary Fig. S11b, c**). The error ratio of the sequencing reads was relatively high in the ending position (**Supplementary Fig. S11d**).

OTU cluster and species annotation

The remaining high-quality sequences were used to generate OTUs by Uparse software (version 7.0.1001) [59] with an identity cutoff of 97%. Singleton OTUs (OTUs found in only one sample) that did not match the reference database were removed. Clustering across all samples from the 128 chickens produced 46,480 OTUs after singleton removal. Nonetheless, most of those OTUs were present in low abundance and were found in very few samples. We then discarded OTUs that were not found in at least 20% of the chickens in each sampling site, yielding 6,776 OTUs (**Supplementary Fig. S1a, b**). For each OTU, the SSUrRNA library in the Silva (https://www.arb-silva.de/) [60] was used to annotate taxonomic information (i.e., kingdom, phylum, class, order, family, genus and species) based on the Mothur algorithm [61]. Subsequently, we determined the phylogenetic relationship of different OTUs and dominant species differences in samples (groups) after multiple sequence alignment using MUSCLE software (version 3.8.31) [62]. Additionally, OTU abundance information was normalized using a standard sequence number corresponding to the sample with the fewest sequences.

Alpha diversity

Alpha diversity was used to analyze the complexity of species diversity for a sample based on normalized OTUs through six indices (i.e., observed OTUs, ACE, Chao1, Simpson, Shannon, and Good's coverage), using the QIIME2 software [63]. Among these, Chao1 and ACE were selected to identify community richness, Shannon and Simpson were used to identify community diversity, and Good's coverage was used to characterize sequencing depth. Differences in alpha diversity indices among the six sites were calculated with the Wilcoxon rank-sum test using R software (version 2.15.3).

Beta diversity

Beta diversity was used to evaluate differences in samples. Beta diversity in BC and weighted/unweighted UniFrac distances were calculated using QIIME2 software [63]. The BC ordination provided position values along an ordination axis and distances from the axis for samples of communities.

Principal coordinates analysis (PCoA)

PCoA was performed to obtain principal coordinates and to visualize complex, multidimensional data. A distance matrix of previously obtained weighted/unweighted UniFrac distances among samples was transformed to a new set of orthogonal axes, by which the maximum variation factor was demonstrated by the first principal coordinate, the second maximum variation factor was demonstrated by the second principal coordinate, and so on. PCoA was performed using the WGCNA package [64], stat packages, and ggplot2 package in R software.

Prediction of the functional profiles of microbial communities

The functions of the microorganisms present in the microbial communities detected in the six sites were predicted using PICRUSt2 [65]. We used the Wilcoxon rank-sum test to investigate differences in pathways among sites. *P*-values were adjusted using the Benjamini-Hochberg method by the FDR with the p.adjust function in R.

Community difference analysis

Pairwise comparisons between different sites were statistically compared using ANOSIM (also named permutational MANOVA) with 10,000 permutations based on BC ordination to evaluate the reasonability of the division of groups.

Between-group variation analysis

High-dimensional biomarkers were discovered by LEfSe using the parameter 'LDA score > 4' [33] to identify characteristics of abundance and related classes (e.g., genes, metabolites, or taxa).

Identification of microbiota constituents related to egg production

EN300 values between two groups (the lowest- and highest-ranked 20% of chickens with respect to their EN300 value) were then compared using the Wilcoxon rank-sum test. Microorganisms with $P < 0.05$ and FDR < 0.05 were retained. Furthermore, we calculated the Spearman's r and Pearson's r between EN300 and the abundance of each

microbiota constituent at genus, OTU, and species levels. A significant correlation between the presence of a microorganism and the EN300 value was considered if $P < 0.05$, as determined using the psych package in R with the P value adjusted using the Benjamini-Hochberg method. Overlapping microorganisms obtained from the Wilcoxon rank-sum test and Spearman's *r* and Pearson's *r* were considered to have a potential relationship with EN300. We subsequently characterized EN300-associated microbes in the six sites.

Whole-genome sequencing data processing

To avoid analysis noise caused by sequencing errors, low-quality paired reads (reads with ≥ 10% unidentified nucleotides [N]; > 10 nt aligned to the adaptor, allowing ≤ 10% mismatches; > 50% bases having phred quality < 5; and putative PCR duplicates generated in the library construction process), which mainly resulted from base-calling duplicates and adaptor contamination, were removed using an in-house script [66]. Consequently, 1.30 terabases (~10.15-fold per individual) of high-quality paired-end reads were obtained, including 95.13% and 88.98% nucleotides with phred quality ≥ Q20 (with an accuracy of 99.00%) and ≥Q30 (with an accuracy of 99.90%), respectively (**Supplementary Table S3**).

Read mapping, and genomic variant calling and annotation

The remaining high-quality reads of each individual were aligned to the reference chicken genome (Gallus_gallus-6.0 Ensembl release 98, http://asia.ensembl.org/Gallus_gallus/Info/Index/) using the Burrows-Wheeler Alignment tool (BWA) (version 0.7.15) [67] with the command 'mem -t 10 -k 32'.

BAM alignment files were then generated using SAMtools (version 0.1.19) [67]. Additionally, we improved alignment performance through filtering the alignment reads with mismatches ≤ 5 and mapping quality = 0. After sorting by SAMtools, the sorted BAM file was marked in duplicate using the command "MarkDuplicates" in the package Picard (version 1.119).

Subsequently, we performed gVCF calling in accordance with the Genome Analysis Toolkit (GATK) best practices pipeline (version v3.7) [68] using the HaplotypeCaller-based method, and then population SNP calling by merging all gVCFs with the commands "CombineGVCFs".

To obtain high-credibility SNPs, we applied the hard filter command 'VariantFiltration' to exclude potential false-positive variant calls as follows: (a) quality by depth >10.0; (b) mapping quality score > 40.0; (c) FS < 60.0; (d) MQRank-Sum > −12.5; (e) ReadPosRankSum > −8.0. In addition, In addition, we further removed the SNPs with adjacent distances ≤ 5 [69]. Finally, we used vcftools (version 0.1.15) to obtain biallelic variants with the following parameters: sample call rate > 90%, SNP call ratio > 95%, minor allele frequencies > 1%, and Hardy-Weinberg equilibrium *P* value < 10^{-5}. Ultimately, a total of 10.82 M high-credibility SNPs in 128 individuals were retained (**Supplementary Table S9**). SNPs were classified into different genomic regions (i.e., exonic, intronic, splice sites, upstream and downstream around gene regions, and intergenic) using the ANNOVAR package [70].

Construction of microbial relationship and host genetic relatedness matrices

OTUs identified in each site were normalized to a zero mean and unit variance. We then constructed a MRM [71] using an R script based on the following equation:

$$r_{tij} = \frac{1}{N_T}\sum_{o=1}^{N_T}\frac{(a_{tio} - \overline{t_{to}})(a_{tjo} - \overline{a_{to}})}{\sigma_{to}^2}$$

where r_{tij} represents the tested microbial relationship in tract t between chickens i and j; a_{tio} and a_{tjo} are the abundance of OTU o in tract t in chickens i and j, respectively; $\overline{t_{to}}$ is the average relative abundance of OTU o in tract t in the population; σ_{to}^2 is the variance in the abundance of OTU o in tract t; and N_T is the total number of OTUs in tract t used for the computation of relatedness. High-quality SNPs were further used to detect independent markers using PLINK [72], with the following parameters: 50 kb window size, 10 SNPs per step, and 0.2 as a squared Pearson's *r* (r^2). All 10,809,968 SNPs were used to compute the principal components (PCs) and GRM [73] using GCTA version 1.91.1 [74]:

$$h_{ij} = \frac{1}{N}\sum_{a=1}^{N}\frac{(r_{ia} - 2\overline{f_a})(r_{ja} - 2\overline{f_a})}{2\overline{f_a}(1 - \overline{f_a})}$$

where h_{ij} is the tested genetic relationship between chickens i and j; r_{ia} and r_{ja} represent the number of reference alleles in chickens i and j, respectively; $\overline{f_a}$ is the frequency of the reference allele in the population; and N is the number of variants.

Heritability (h^2) analysis

To estimate the effects of host genetics on the microbiota at different sites, we computed the correlation between GRM and BC distances at each site using both Pearson's *r* and Spearman's *r*, based on Mantel tests with 10,000 permutations. The correlation between GRM and MRM was also computed. To estimate the correlation between GRM and the microbiota community, we computed heritability at OTU, genus, and species levels. OTU abundance information was normalized using a standard sequence number

corresponding to the sample with the least number of sequences. Microorganisms that were present in < 60% but ≥ 20% of the samples were dichotomized as present or absent [75], and the microorganisms that were detected in < 20% of the samples from each site were excluded from the analysis.

Genetic and microbial parameters of egg production

As the individuals examined in this study had no pedigree information, we computed the SNP-based heritability of the egg production phenotype (i.e., EN300) instead, using the following model [73]:

$$y = K_c + g + e[A]$$

where y is an observed value (EN300); c is a vector of fixed covariates with the corresponding design matrix K; e is the residual effect; and g is a vector of aggregate effects of all SNPs with an ~N(0,$G_{\sigma_A^2}$), where G and σ_A^2 are the GRM and polygenetic variance (overall SNP effects), respectively. The top five host genetic PCs were considered covariates in the model to account for the calculated population stratification, as described above. The likelihood ratio test P value was calculated to examine the significance of the association between SNPs and EN300.

The fraction of EN300 variance explained by microbial variance was calculated as $m^2 = \frac{\sigma_m^2}{\sigma_p^2}$; (called 'microbiability' [m^2] in animals [71] and 'microbiome-association index' in humans [18]), where σ_m^2 and σ_p^2 are the phenotypic variance and microbial variance, respectively. To adjust for host genetic effects, all valid individuals and SNPs were used in a GWAS with a univariate linear mixed model (LMM), which was performed using GEMMA [76]. The LMM was calculated as follows:

$$y=K_c+m_s + e \text{ [B]}$$

where the model parameters are the same as those described in model [A], except m_s, which is the random effect of the microbiota in locations following the multinomial distribution m_s~N(0,Mσ_m^2), and M is the MRM. We then used the MRM in GCTA to calculate m^2. The genome-wide significance threshold was 10^{-6}. We then extracted these SNPs with significant effects on EN300 and calculated the PCs using PLINK. The first two PCs and the top five host genetic PCs were then used as covariates in model [B] to account for host genetics.

RNA sequencing (RNA-seq) analysis

For RNA-seq, total RNA was extracted from uterine tissue of twelve hens (six for each of the groups with either the 20% lowest or 20% highest EN300 values) using the RNeasy Mini Kit (Qiagen). We used an rRNA depletion protocol (Ribo-Zero kit, Epicenter) coupled with the Illumina TruSeq stranded RNA-seq library protocol to construct the RNA-seq libraries. A total of 12 libraries were quantified using the Qubit dsDNA High Sensitivity Assay Kit (Invitrogen) and separately sequenced on the NovaSeq 6000 platform (Illumina) to produce an average of ~31.86 M 150-bp paired-end raw reads and ~30.52 M high-quality reads for each library. Sequence reads were aligned to the chicken reference genome (Gallus_gallus-6.0 Ensembl release 98) by the STAR alignment tool (version 2.5.3a). On average, ~96% of reads of individual libraries were aligned to the chicken reference genome, generating an average of 29.30 M aligned reads for each sample. The gene expression level was then estimated as

transcripts per million (TPM) using the high-speed transcript quantification tool Kallisto (V0.43.0) [77].

We used the edgeR package [78] to identify differentially expressed genes (FDR < 0.01 and |log2(fold change)| ≥ 1) between the two groups with either the 20% lowest or 20% highest EN300 values. Functional enrichment analysis of differentially expressed genes was performed using the Metascape tool [79]. Only Gene Ontology (GO) terms and KEGG pathways with a *P* value < 0.05 were considered significant and are listed.

The expression levels of eight genes were verified using a quantitative PCR (q-PCR) approach. The β-actin gene of chicken was used as an endogenous control gene. Relative expression levels of objective mRNAs were calculated using the ΔΔCt method. The primer sequences used for q-PCR are shown in **Supplementary Table S10**. All measurements contained a negative control (no cDNA template), and each RNA sample was analyzed in triplicate.

Figure Legends

Figure 1. Diversity and composition of the reproductive and digestive tract microbiota in chickens. (**a**) Relative abundance of the microbiota from six sites at the genus level. Only genera with an abundance > 1% in a site are shown. (**b**) Alpha diversity comparison based on the Shannon diversity index (**P* < 0.05; ***P* < 0.01, Wilcoxon rank-sum test). **c.** Principal coordinates analysis of the 768 samples based on weighted UniFrac distances. 34.96% of variance was explained for component 1 (*P* < 0.05, Tracy-Widom test) and 24.23% for component 2 (*P* < 0.05, Tracy-Widom test).

Figure 2. Comparison of predicted functional capacities and site-associated taxa of microbial communities. (**a**) Heatmap showing the predicted KEGG pathways and their abundances at reproductive and digestive tract sites (Supplementary Table S2). Z scores indicate the means of KEGG pathway abundances. (**b**) Heatmap showing the 65 site-associated bacterial taxa identified by LEfSe (LDA > 4). Z scores indicate the relative abundances of site-associated bacterial taxa. Black frames represent site-associated bacterial taxa whose *Z* scores of relative abundances differed significantly among the six sites. p, phylum; c, class; o, order; f, family; g, genus.

Figure 3. Effect of host genetics on microbiota of the reproductive and digestive tracts. (**a-f**) Density scatter plots of genetic kinship of pairs of individuals (x axis) and their microbiome dissimilarity (y axis) among all pairs of individuals (n = 16,256). NS, not significant ($P > 0.05$; Mantel test). (**g-i**) Distribution of species, genera, and OTUs identified in six sites for all chickens. Microorganisms present in < 20% of samples were excluded. (**j-l**) Proportion of heritable microbial genera, species, and OTUs in each site. **(m-o)** Cumulative relative abundances of heritable microbial genera, species, and OTUs in each site.

Figure 4. Microorganisms associated with egg number at 300 days of age (EN300). (**a**) Microbial species associated with EN300 ($P < 0.05$) among the six sites. **(b)** Pearson's r values between EN300 and 24 EN300-associated microbial species; only significant r values are given numerically ($P < 0.05$). **c** Pearson's r (lower diagonal) and Spearman's r (upper diagonal) values among microbial species in the uterus (UT); significant r values are given numerically ($P < 0.05$).

Figure 5. Effect of microbial species associated with EN300. (**a**) EN300 values for the 20% of chickens with the highest and lowest abundances of *B. fragilis*, *B. salanitronis*,

B. barnesiae, *C. leptum*, and *Firmicutes* bacterium ZOR0006 in the three reproductive tract sites. The plots show the median, as well as the 25% and 75% quantiles. The cross and horizontal lines indicate the mean and median values in the corresponding group, respectively. Significance levels were calculated using a permutation test with 10,000 replicates. *$P < 0.05$; NS, not significant. (**b**) Relative abundance and detected ratio of five species (*B. fragilis*, *B. salanitronis*, *B. barnesiae*, *C. leptum* and *Firmicutes* bacterium ZOR0006) in the six sites. Blue bars indicate the detection ratio of each species at each site.

Figure 6. Microorganisms influence the expression of immune-related genes in the uterus. **(a)** Hierarchical clustering and (**b**) pairwise Pearson's correlations of 12 samples using transcriptional profiles. **(c)** Differentially expressed genes between groups with high and low egg production. Eight well-documented inflammatory markers are labeled. (**d**) Comparison of expression changes of eight inflammatory markers in the uterus between groups with high and low egg production by a q-PCR approach. **(e)** Top 20 functional categories enriched for 739 genes that were significantly downregulated in the high-egg production group. The enrichment analysis was performed using the Metascape tool (see Method). GO-BP: biological process (blue) and GO-MF: molecular function (yellow).

Data Availability

The sequencing data for this project have been deposited in the NCBI and can be accessed with BioProject Nos. PRJNA730194 (Microbiome), PRJNA731001 (Whole genome resequencing) and PRJNA730355 (RNA-seq). Other data further supporting this work are openly available in the GigaScience repository, GigaDB [80].

Additional Files

Supplementary Figure S1. The number distribution of OTUs with different existing ratio of samples. (**a**) The number plot of OTUs with different existing ratio of samples. (**b**) The relationship of existing ratio and the slope of the curve in **a**. The dotted line indicated the threshold utilized to remove existing ratio distribution trend due to fluctuate greatly.

Supplementary Figure S2. Alpha diversity, relative abundance, and Spearman's *r* values of specific microbiota among the six sites. (**a-e**) Alpha diversity comparison based on Good's coverage, observed OTU，ACE, Chao1, and Simpson indices, using Wilcoxon rank-sum test to determine significant differences. (**f**) Rarefaction curves of observed OTU. (**g**) Alpha diversity values of the six sites. Values are represented as median ± SD. (**h**) *P* values of Wilcoxon rank-sum test of each comparison for six alpha diversity indices. (**i**) PCoA of the 768 samples based on unweighted UniFrac distances. (**j**) Beta diversity comparison based on the weighted UniFrac distances among the six sites. The values are filled with weighted UniFrac distances (mean ± SD) in the corresponding comparisons. All comparisons were significantly different using Wilcoxon rank-sum test ($P < 0.05$). (**k**) Relative abundance of the top ten dominant microbial phyla in the six sites. (**l**) Only microbial genera that were present in at least 461 samples (60% of the total) were plotted. Each row represents a microorganism. Among 2,475 Spearman's *r* values, only 362 (14.62%) were significantly correlated ($P < 0.05$).

Supplementary Figure S3. Comparison of the functional capacities of the reproductive and digestive microbial communities among the six sites. (**a**) Overlap of the top 50 predictions among the six sites. (**b**) Heatmap showing the 36 overlapped predictions with different abundances among the six sites. The heatmap is color-coded based on row *Z* scores. (**c**) Map showing 65 site-associated bacterial taxa identified by LEfSe (LDA score > 4) in the test trial.

Supplementary Figure S4. Significantly heritable microorganisms. The number of significantly heritable microorganism OTUs, genera, and species ($P < 0.05$) grouped by sampling phyla (**a, c, e**) and site (**b, d, f**).

Supplementary Figure S5. Significantly EN300-associated microorganisms. The number of microorganisms significantly associated with EN300 detected at OTU, genus, and species ($P < 0.05$) levels grouped by sampling phyla (**a, c, e**) and site (**b, d, f**).

Supplementary Figure S6. EN300-associated microorganisms. (**a**) The number of microbial genera (left) and OTUs (right) associated with EN300 at $P < 0.05$ of three test methods of six sites and their overlap. (**b-c**) Pearson's and Spearman's *r* values between EN300 and EN300-associated 26 genera and 39 OTUs. Red and blue tiles indicate positive and negative correlations, respectively. Significant *r* values are filled in numerically ($P < 0.05$).

Supplementary Figure S7. Pearson correlations between EN300 and EN300-associated microorganisms. (**a**) Pearson' s *r* values of candidate microbial species in the six sites. (**b**) Pearson' s *r* values among microbial species in each site. CP: Crop, GZ: Gizzard, SI: small intestine, UT: uterus, IS: Isthmus, VA: vagina. Red and blue tiles

indicate positive and negative correlations, respectively. The ratios on the right side of each site represents the number of significant correlations. *$P < 0.05$.

Supplementary Figure S8. Differences in the relative abundance of five species between the 20% of chickens with the highest and lowest egg production (EN300). (**a**) EN300 values for the 20% of individuals with the highest and lowest egg production. (**b**) EN300 values for the 20% of individuals with the highest and lowest abundances of *Clostridium leptum*, *Bacteroides salanitronis*, *Firmicutes* bacterium ZOR0006, *B. barnesiae*, and *B. fragilis* in the three reproductive tract sites. All comparisons were significantly different, established at $P < 0.05$.

Supplementary Figure S9. Top 20 functional categories enriched by 1,051 genes exhibited significant expression changes between groups with the high- and low- egg production. The enrichment analysis was performed using the Metascape tool (See Method). GO-BP: biological process (blue), GO-MF: molecular function (yellow) and KEGG (red).

Supplementary Figure S10. Distribution of egg number at 300 days of age (EN300). Compared to the 20% of hens with the highest EN300 values, the 20% of hens with the lowest EN300 values exhibited a later start laying age, an earlier stop aging day and irregular lay performance.

Supplementary Figure S11. Quality assessment of sequencing data. (**a**) Length distribution of reads. (**b**) Quality score of each base. (**c**) Quality score distribution of sequencing data. (**d**) Error rate distribution of reads.

Supplementary Table S1. Analysis of Bray-Curtis distance similarities.

Supplementary Table S2. Statistical test for the 65 functional capacities among the six sites.

Supplementary Table S3. Summary of host whole genome sequencing.

Supplementary Table S4. Correlation between genetic relatedness matrix (GRM) and each Bray-Curtis (BC) distance or microbial relationship matrix (MRM) by Mantel test.

Supplementary Table S5. Heritability (h^2) of the microbiota and cumulative abundance of heritable microbiota.

Supplementary Table S6. Heritability (h^2) of reproductive traits from previous reports.

Supplementary Table S7. Summary of 16S rDNA sequencing.

Supplementary Table S8. Summary statistics of 16S rDNA sequencing.

Supplementary Table S9. Summary statistics of host whole genome sequencing.

Supplementary Table S10. Primer sequences for q-PCR.

Abbreviations

QTLs: quantitative trait loci; h^2: heritability; GWAS: genome-wide association study; *PLAG1*: pleiomorphic adenoma gene 1; *LYN*: lck/yes-related novel tyrosine kinase gene; EN300: egg number at 300 days of age; M: million; OTUs: operational taxonomic units; BC: Bray-Curtis; PCoA: Principal coordinates analysis; FDR: false discovery rate; ANOSIM: Analysis of Similarity; LDA: Linear discriminant analysis; LEfSe: Linear discriminant analysis Effect Size; BWA: Burrows-Wheeler Alignment tool; GATK: Genome Analysis Toolkit; SNP: single nucleotide polymorphism; MRM: microbial relationship matrix; PCs: principal components; GRM: genetic relatedness matrix; m^2:

microbiability; LMM: linear mixed model; RNA-seq: RNA sequencing; TPM: transcripts per million; GO: Gene Ontology; KEGG: Kyoto Encyclopedia of Genes and Genomes; q-PCR: quantitative PCR; CPSA: capsular polysaccharide A.

Ethics Statement

All animal experiments were approved and reviewed by Animal Care and Use Committee Institutional of Sichuan Agricultural University (Approval No. DKY-2018102015).

Competing Interests

The authors declare that they have no competing interests.

Funding

This work was supported by the Sichuan Science and Technology Program (2019JDTD0009, 2020YFH0138 and 2021YFYZ0009), the Fok Ying-Tong Education Foundation for Young Teachers in the Higher Education Institutions of China (161026).

Authors' Contributions

D.L., M.L. and Q.Z. designed the study; T.W., S.K.M., Y.S., W.Z., and S.T. wrote the manuscript; Z.X., M.H., X.Z., H.Y., X.F., and Q.N. collected the sample and extracted the DNA. M.Y., D.Y., Y.L., B.Z, and M.Z., did bioinformatics analyses; D.L. wrote methods; D.L., M.L. and Q.Z. supervised the work.

Acknowledgements

We thank the High-Performance Computing Platform of Sichuan Agricultural University and Ya'an Big Data Industrial Park for providing computing resources and support that have contributed to these research results.

References

1. Wolc A, Arango J, Settar P, et al. Analysis of egg production in layer chickens using a random regression model with genomic relationships. Poult Sci 2013;**92**(6):1486-1491.
2. Hu ZL, Park CA and Reecy JM. Building a livestock genetic and genomic information knowledgebase through integrative developments of Animal QTLdb and CorrDB. Nucleic Acids Res 2019;**47**(D1):D701-D710.
3. Yuan J, Sun C, Dou T, et al. Identification of Promising Mutants Associated with Egg Production Traits Revealed by Genome-Wide Association Study. PLoS One 2015;**10**(10):e0140615.
4. Zhang GX, Fan QC, Wang JY, et al. Genome-wide association study on reproductive traits in Jinghai Yellow Chicken. Anim Reprod Sci 2015;**163**:30-34.
5. Tongsiri S, Jeyaruban MG and Van Der Werf JH. Genetic parameters for egg production traits in purebred and hybrid chicken in a tropical environment. Br Poult Sci 2015;**56**(6):613-620.
6. Savegnago RP, Caetano SL, Ramos SB, et al. Estimates of genetic parameters, and cluster and principal components analyses of breeding values related to egg production traits in a White Leghorn population. Poult Sci 2011;**90**(10):2174-2188.
7. Chen C, Song X, Wei W, et al. The microbiota continuum along the female reproductive tract

and its relation to uterine-related diseases. Nat Commun 2017;**8**(1):875.

8. Wen C, Li Q, Lan F, et al. Microbiota continuum along the chicken oviduct and its association with host genetics and egg formation. Poult Sci 2021;**100**(7):101104.
9. Fettweis JM, Serrano MG, Brooks JP, et al. The vaginal microbiome and preterm birth. Nat Med 2019;**25**(6):1012-1021.
10. Serrano MG, Parikh HI, Brooks JP, et al. Racioethnic diversity in the dynamics of the vaginal microbiome during pregnancy. Nat Med 2019;**25**(6):1001-1011.
11. Gotsch F, Romero R, Kusanovic JP, et al. The fetal inflammatory response syndrome. Clin Obstet Gynecol 2007;**50**(3):652-683.
12. Lamont RF, Sobel JD, Akins RA, et al. The vaginal microbiome: new information about genital tract flora using molecular based techniques. BJOG 2011;**118**(5):533-549.
13. Den Hartog G, De Vries-Reilingh G, Wehrmaker AM, et al. Intestinal immune maturation is accompanied by temporal changes in the composition of the microbiota. Benef Microbes 2016;**7**(5):677-685.
14. Choi JH, Kim GB and Cha CJ. Spatial heterogeneity and stability of bacterial community in the gastrointestinal tracts of broiler chickens. Poult Sci 2014;**93**(8):1942-1950.
15. Lee S, La T-M, Lee H-J, et al. Characterization of microbial communities in the chicken oviduct and the origin of chicken embryo gut microbiota. Sci Rep 2019;**9**(1):6838.
16. Elokil AA, Magdy M, Melak S, et al. Faecal microbiome sequences in relation to the egg-laying performance of hens using amplicon-based metagenomic association analysis. Animal 2020;**14**(4):706-715.
17. Carmody RN, Gerber GK, Luevano JM, Jr., et al. Diet dominates host genotype in shaping the

murine gut microbiota. Cell Host Microbe 2015;**17**(1):72-84.

18. Rothschild D, Weissbrod O, Barkan E, et al. Environment dominates over host genetics in shaping human gut microbiota. Nature 2018;**555**(7695):210-215.
19. Liu H, Chen Z, Gao G, et al. Characterization and comparison of gut microbiomes in nine species of parrots in captivity. Symbiosis 2019;**78**(3):241-250.
20. Ji J, Luo CL, Zou X, et al. Association of host genetics with intestinal microbial relevant to body weight in a chicken F2 resource population. Poult Sci 2019;**98**(9):4084-4093.
21. Mignon-Grasteau S, Narcy A, Rideau N, et al. Impact of Selection for Digestive Efficiency on Microbiota Composition in the Chicken. PLoS One 2015;**10**(8):e0135488.
22. Scepanovic P, Hodel F, Mondot S, et al. A comprehensive assessment of demographic, environmental, and host genetic associations with gut microbiome diversity in healthy individuals. Microbiome 2019;**7**(1):130.
23. Verstraelen H, Vilchez-Vargas R, Desimpel F, et al. Characterisation of the human uterine microbiome in non-pregnant women through deep sequencing of the V1-2 region of the 16S rRNA gene. PeerJ 2016;**4**:e1602.
24. Anahtar MN, Gootenberg DB, Mitchell CM, et al. Cervicovaginal Microbiota and Reproductive Health: The Virtue of Simplicity. Cell Host Microbe 2018;**23**(2):159-168.
25. Thompson JL and Hinton M. Antibacterial activity of formic and propionic acids in the diet of hens on Salmonellas in the crop. Br Poult Sci 1997;**38**(1):59-65.
26. Khan S, Moore RJ, Stanley D, et al. Gut microbiota of laying hens and its manipulation with prebiotics and probiotics to enhance gut health and food safety. Appl Environ Microbiol 2020;**86**(13):e00600-00620.

27. Mishra AK, Kumar A, Swain DK, et al. Insights into pH regulatory mechanisms in mediating spermatozoa functions. Vet World 2018;**11**(6):852-858.

28. Fiser PS and Macpherson JW. pH values in the oviduct of the hen during egg formation. Poult Sci 1974;**53**(2):827-829.

29. Storek KM and Monack DM. Bacterial recognition pathways that lead to inflammasome activation. Immunol Rev 2015;**265**(1):112-129.

30. Fang S, Zhang L, Lou Y, et al. Intracellular translocation and localization of Edwardsiella tarda type III secretion system effector EseG in host cells. Microb Pathog 2016;**97**:166-171.

31. Yu MD and Lai EM. Warfare between Host Immunity and Bacterial Weapons. Cell Host Microbe 2017;**21**(1):3-4.

32. Chen H, Yang D, Han F, et al. The bacterial T6SS effector EvpP prevents NLRP3 inflammasome activation by inhibiting the Ca2+-dependent MAPK-Jnk pathway. Cell Host Microbe 2017;**21**(1):47-58.

33. Segata N, Izard J, Waldron L, et al. Metagenomic biomarker discovery and explanation. Genome Biol 2011;**12**(6):R60.

34. Moore RW, Park SY, Kubena LF, et al. Comparison of zinc acetate and propionate addition on gastrointestinal tract fermentation and susceptibility of laying hens to Salmonella enteritidis during forced molt. Poult Sci 2004;**83**(8):1276-1286.

35. Kalisperati P, Spanou E, Pateras IS, et al. Inflammation, DNA Damage, Helicobacter pylori and Gastric Tumorigenesis. Front Genet 2017;**8**:20.

36. Franceschi F, Annalisa T, Teresa DR, et al. Role of Helicobacter pylori infection on nutrition and metabolism. World J Gastroenterol 2014;**20**(36):12809-12817.

37. Ansari S and Yamaoka Y. Survival of Helicobacter pylori in gastric acidic territory. Helicobacter 2017;**22**(4):e12386.

38. Sonnenburg JL, Xu J, Leip DD, et al. Glycan foraging in vivo by an intestine-adapted bacterial symbiont. Science 2005;**307**(5717):1955-1959.

39. Chousalkar KK and Roberts JR. Ultrastructural changes in the oviduct of the laying hen during the laying cycle. Cell Tissue Res 2008;**332**(2):349-358.

40. Hrabia A, Lesniak-Walentyn A, Sechman A, et al. Chicken oviduct-the target tissue for growth hormone action: effect on cell proliferation and apoptosis and on the gene expression of some oviduct-specific proteins. Cell Tissue Res 2014;**357**(1):363-372.

41. Wang Y, Xu L, Sun X, et al. Characteristics of the fecal microbiota of high-and low-yield hens and effects of fecal microbiota transplantation on egg production performance. Res Vet Sci 2020;**129**:164-173.

42. Choe D, Loh T, Foo H, et al. Egg production, faecal pH and microbial population, small intestine morphology, and plasma and yolk cholesterol in laying hens given liquid metabolites produced by Lactobacillus plantarum strains. Br Poult Sci 2012;**53**(1):106-115.

43. Lee W-J and Hase K. Gut microbiota–generated metabolites in animal health and disease. Nat Chem Biol 2014;**10**(6):416-424.

44. Pandit RJ, Hinsu AT, Patel NV, et al. Microbial diversity and community composition of caecal microbiota in commercial and indigenous Indian chickens determined using 16s rDNA amplicon sequencing. Microbiome 2018;**6**(1):115.

45. Cui L, Zhang X, Cheng R, et al. Sex differences in growth performance are related to cecal microbiota in chicken. Microb Pathog 2021;**150**:104710.

46. Xiang H, Gan J, Zeng D, et al. Specific Microbial Taxa and Functional Capacity Contribute to Chicken Abdominal Fat Deposition. Front Microbiol 2021;**12**:643025.

47. Salanitro JP, Blake IG and Muirhead PA. Studies on the cecal microflora of commercial broiler chickens. Appl Microbiol 1974;**28**(3):439-447.

48. Reeves AR, Wang GR and Salyers AA. Characterization of four outer membrane proteins that play a role in utilization of starch by Bacteroides thetaiotaomicron. J Bacteriol 1997;**179**(3):643-649.

49. Kollarcikova M, Faldynova M, Matiasovicova J, et al. Different Bacteroides species colonise human and chicken intestinal tract. Microorganisms 2020;**8**(10):1483.

50. Nihira T, Suzuki E, Kitaoka M, et al. Discovery of beta-1,4-D-mannosyl-N-acetyl-D-glucosamine phosphorylase involved in the metabolism of N-glycans. J Biol Chem 2013;**288**(38):27366-27374.

51. Mazmanian SK, Liu CH, Tzianabos AO, et al. An immunomodulatory molecule of symbiotic bacteria directs maturation of the host immune system. Cell 2005;**122**(1):107-118.

52. Partty A, Kalliomaki M, Salminen S, et al. Infantile Colic Is Associated With Low-grade Systemic Inflammation. J Pediatr Gastr Nutr 2017;**64**(5):691-695.

53. Hassan MS and Abdul-Careem MF. Avian Viruses that Impact Table Egg Production. Animals 2020;**10**(10):1747.

54. Reid G, Younes JA, Van der Mei HC, et al. Microbiota restoration: natural and supplemented recovery of human microbial communities. Nat Rev Microbiol 2011;**9**(1):27-38.

55. Lee KN and Lee OY. Intestinal microbiota in pathophysiology and management of irritable bowel syndrome. World J Gastroenterol 2014;**20**(27):8886-8897.

56. Caporaso JG, Lauber CL, Walters WA, et al. Global patterns of 16S rRNA diversity at a depth of millions of sequences per sample. Proc Natl Acad Sci U S A 2011;**108**:4516-4522.
57. Martin M. Cutadapt removes adapter sequences from high-throughput sequencing reads. EMBnet J 2011;**17**(1):10-12.
58. Edgar RC, Haas BJ, Clemente JC, et al. UCHIME improves sensitivity and speed of chimera detection. Bioinformatics 2011;**27**(16):2194-2200.
59. Edgar RC. UPARSE: highly accurate OTU sequences from microbial amplicon reads. Nature Methods 2013;**10**(10):996-998.
60. Quast C, Pruesse E, Yilmaz P, et al. The SILVA ribosomal RNA gene database project: improved data processing and web-based tools. Nucleic Acids Res 2013;**41**:D590-D596.
61. Kozich J, Westcott S, Baxter N, et al. Development of a dual-index sequencing strategy and curation pipeline for analyzing amplicon sequence data on the MiSeq Illumina sequencing platform. Appl Environ Microbiol 2013;**79**(17):5112-5120.
62. Edgar RC. MUSCLE: multiple sequence alignment with high accuracy and high throughput. Nucleic Acids Res 2004;**32**(5):1792-1797.
63. Bolyen E, Rideout J, Dillon M, et al. Reproducible, interactive, scalable and extensible microbiome data science using QIIME 2. Nat Biotechnol 2019;**37**(8):852-857.
64. Langfelder P and Horvath S. WGCNA: an R package for weighted correlation network analysis. BMC Bioinformatics 2008;**9**:559.
65. Douglas GM, Maffei VJ, Zaneveld J, et al. PICRUSt2: An improved and extensible approach for metagenome inference. BioRxiv 2019:672295.
66. Li M, Tian S, Jin L, et al. Genomic analyses identify distinct patterns of selection in

domesticated pigs and Tibetan wild boars. Nat Genet 2013;**45**(12):1431-1438.

67. Li H and Durbin R. Fast and accurate long-read alignment with Burrows–Wheeler transform. Bioinformatics 2010;**26**(5):589-595.

68. McKenna A, Hanna M, Banks E, et al. The Genome Analysis Toolkit: a MapReduce framework for analyzing next-generation DNA sequencing data. Genome Res 2010;**20**(9):1297-1303.

69. Li H, Ruan J and Durbin R. Mapping short DNA sequencing reads and calling variants using mapping quality scores. Genome Res 2008;**18**(11):1851-1858.

70. Wang K, Li M and Hakonarson H. ANNOVAR: functional annotation of genetic variants from high-throughput sequencing data. Nucleic Acids Res 2010;**38**(16):e164.

71. Camarinha-Silva A, Maushammer M, Wellmann R, et al. Host Genome Influence on Gut Microbial Composition and Microbial Prediction of Complex Traits in Pigs. Genetics 2017;**206**(3):1637-1644.

72. Purcell S, Neale B, Todd-Brown K, et al. PLINK: a tool set for whole-genome association and population-based linkage analyses. Am J Hum Genet 2007;**81**(3):559-575.

73. Yang J, Bakshi A, Zhu Z, et al. Genetic variance estimation with imputed variants finds negligible missing heritability for human height and body mass index. Nat Genet 2015;**47**(10):1114-1120.

74. Yang J, Lee SH, Goddard ME, et al. GCTA: a tool for genome-wide complex trait analysis. Am J Hum Genet 2011;**88**(1):76-82.

75. Zierer J, Jackson MA, Kastenmuller G, et al. The fecal metabolome as a functional readout of the gut microbiome. Nat Genet 2018;**50**(6):790-795.

76. Zhou X and Stephens M. Genome-wide efficient mixed-model analysis for association studies.

Nat Genet 2012;**44**(7):821-824.

77. Bray NL, Pimentel H, Melsted P, et al. Near-optimal probabilistic RNA-seq quantification. Nat Biotechnol 2016;**34**(5):525-527.

78. Robinson MD, McCarthy DJ and Smyth GK. edgeR: a Bioconductor package for differential expression analysis of digital gene expression data. Bioinformatics 2010;**26**(1):139-140.

79. Zhou Y, Zhou B, Pache L, et al. Metascape provides a biologist-oriented resource for the analysis of systems-level datasets. Nat Commun 2019;**10**(1):1523.

80. Su Y; Tian S; Li D; Zhu W; Wang T; Mishra SK; Wei R; Xu Z; He M; Zhao X; Yin H; Fan X; Zeng B; Yang M; Yang D; Ni Q; Li Y; Zhang M; Zhu Q; Li M: Supporting data for "Female reproductive tract microbiota influence egg production in layer chickens" GigaScience Database. 2021. http://dx.doi.org/10.5524/100928.

Figure 1

Click here to access/download;Figure;Figure 1.tif

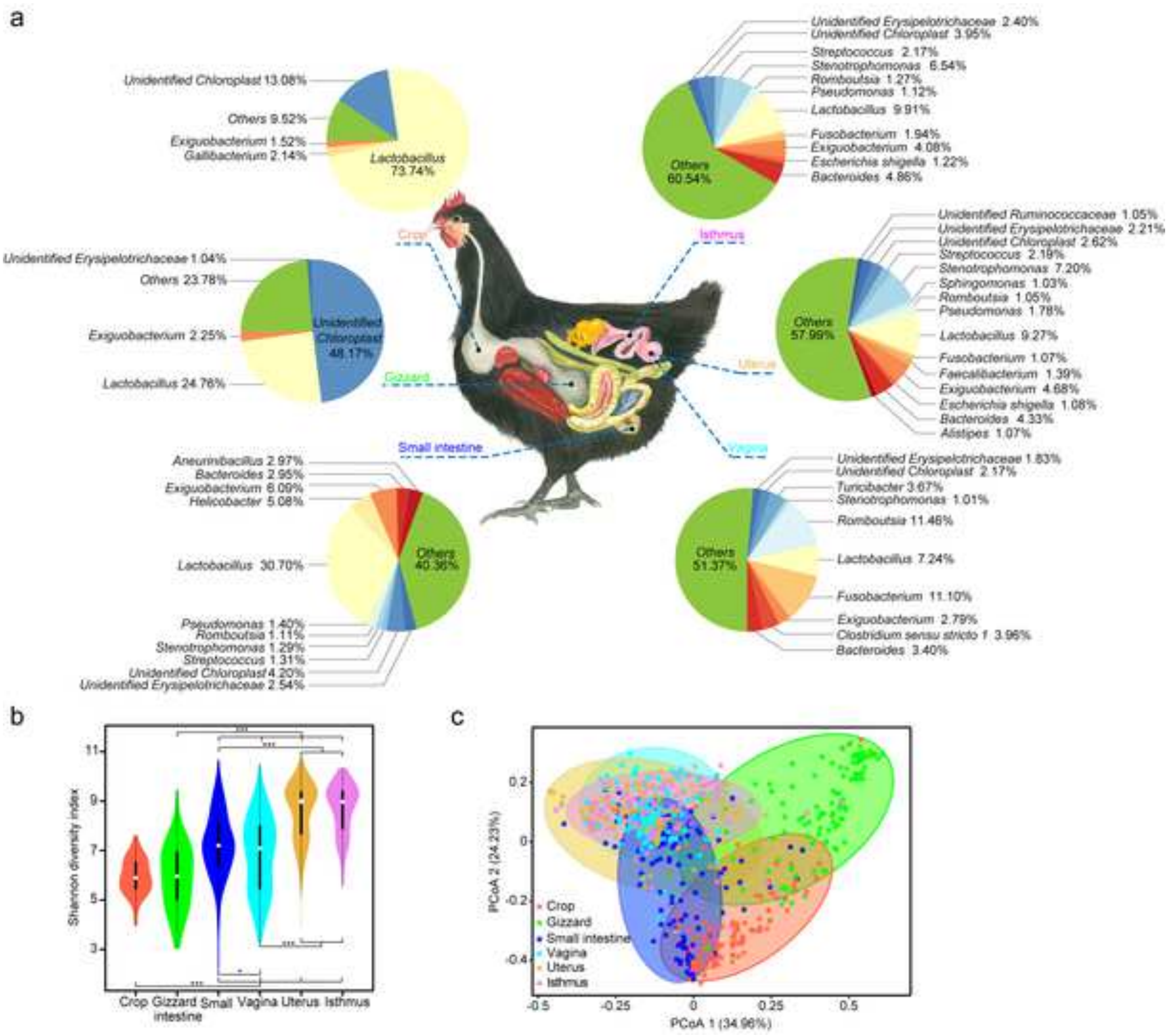

Figure 2

Click here to access/download;Figure;Figure 2.tif

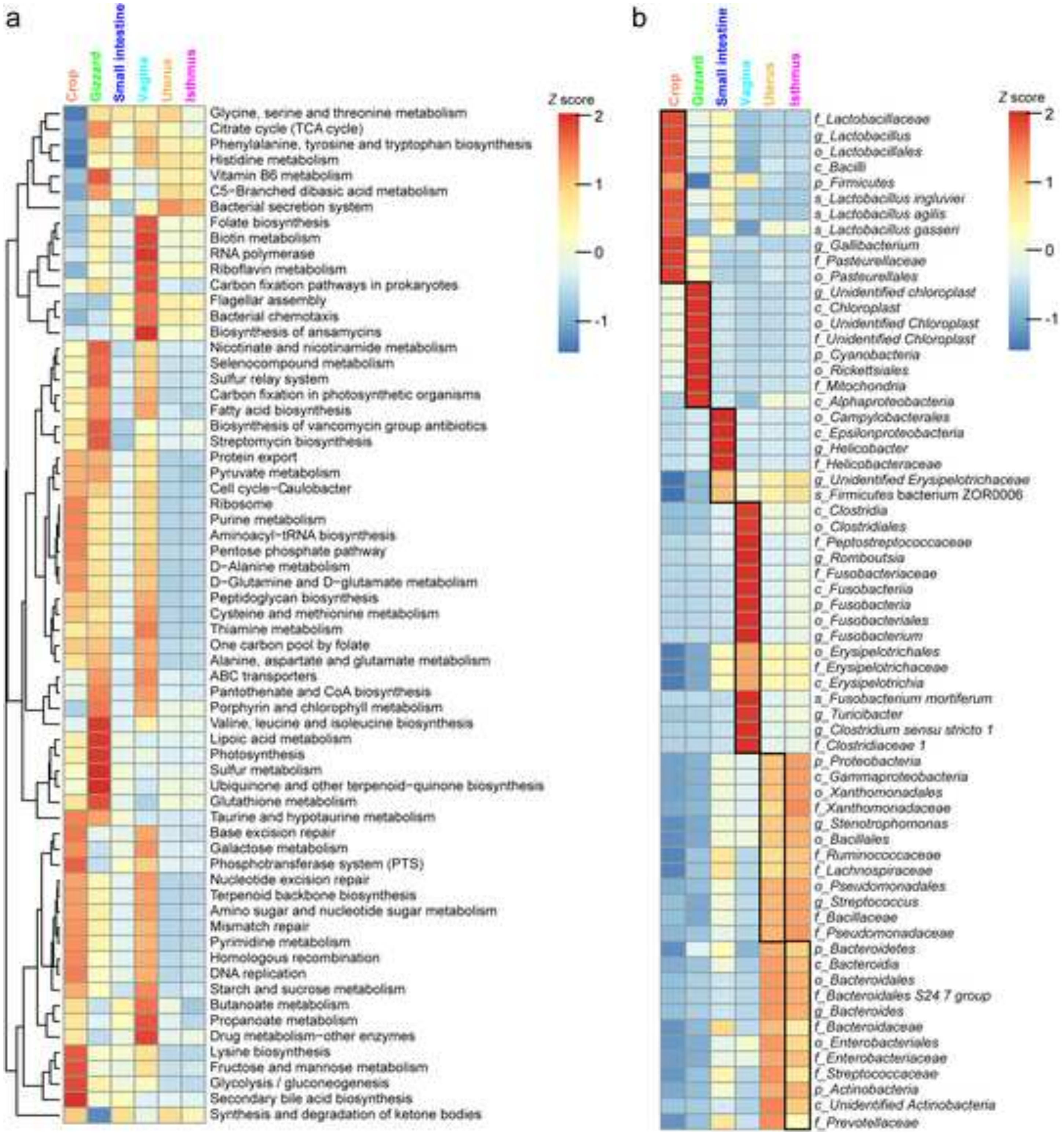

Figure 3

Click here to access/download;Figure;Figure 3.tif

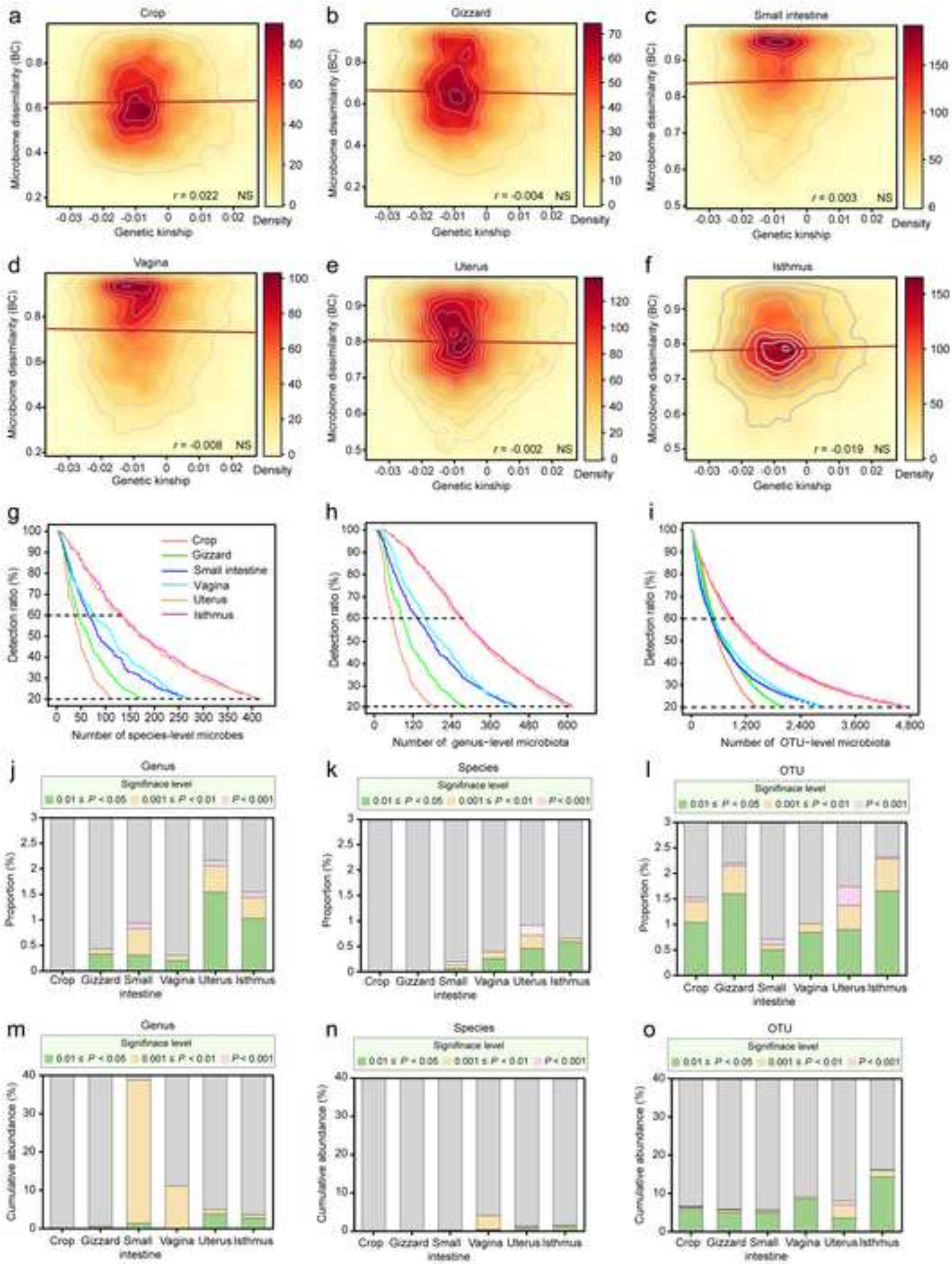

Figure 4 Click here to access/download;Figure;Figure 4.tif

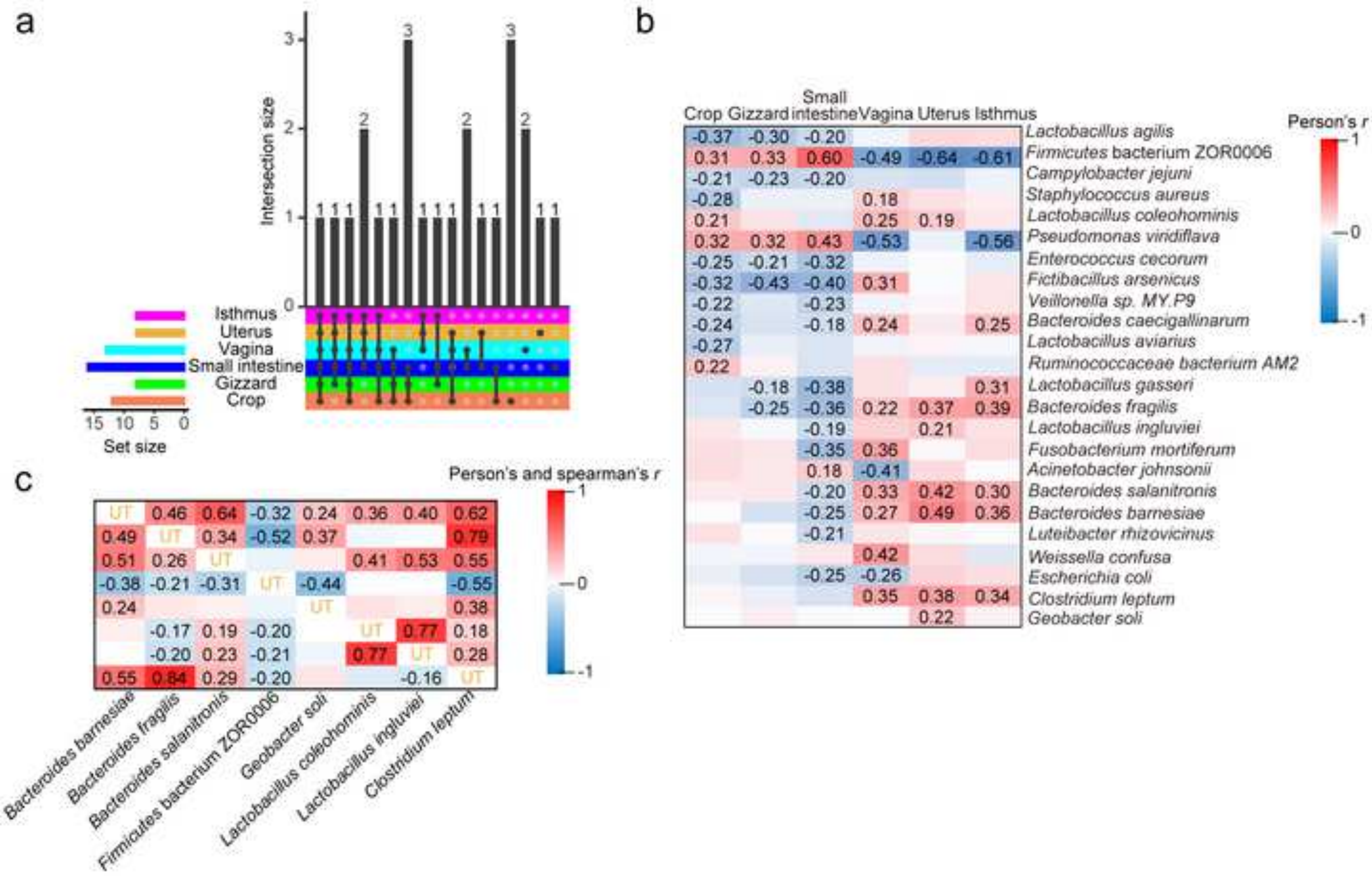

Click here to access/download;Figure;Figure 5.tif

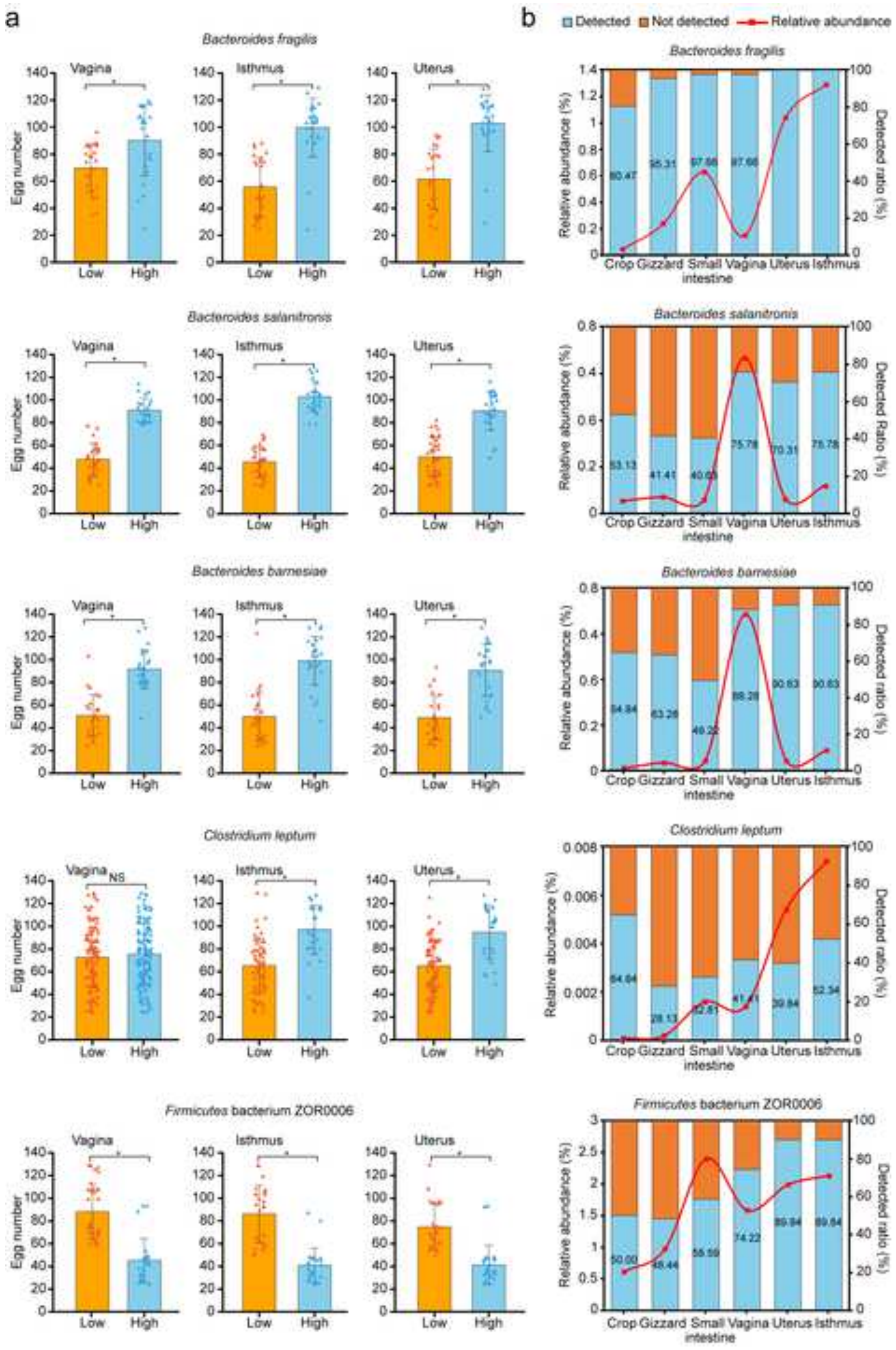

Figure 6

Click here to access/download;Figure;Figure 6.tif

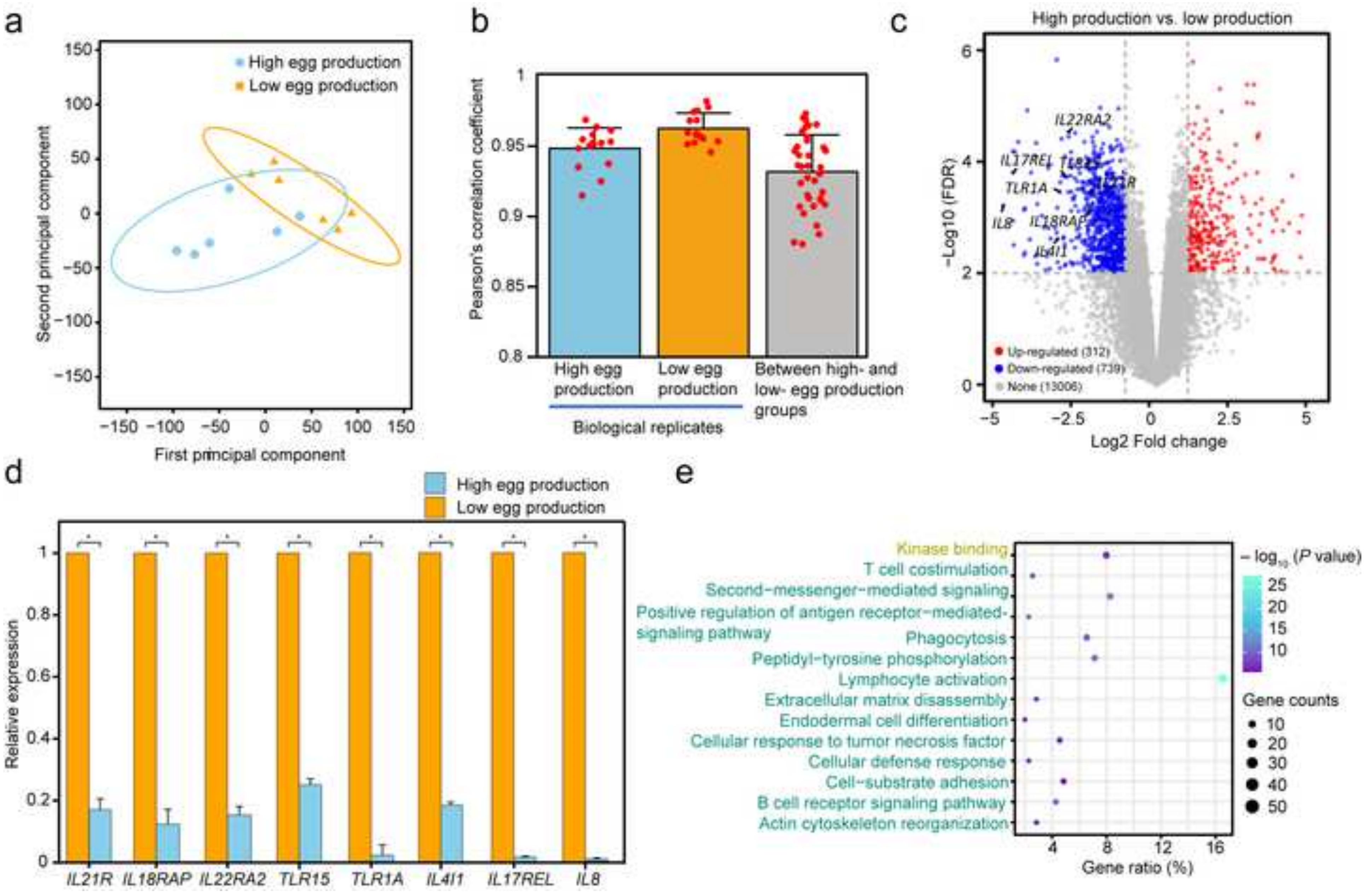

Click here to access/download
Supplementary Material
Supplementary Figures.docx

Supplementary Tables

Click here to access/download
Supplementary Material
Supplementary Tables.xlsx

 Click here to access/download;Personal Cover;Respone letter.docx

Dear editor and reviewers,

Thank you very much for reviewing our manuscript "*Female reproductive tract microbiota influence egg production in layer chickens*" (ID: GIGA-D-21-00132) for possible publication in *GigaScience*. We sincerely thank the editor and two reviewers for their valuable feedback that we have used to improve the quality of our manuscript. According to the reviewers' comments, we have made the relevant modifications to our manuscript. All modifications are marked in red in the revised manuscript and a point-by-point response to the reviewers' comments follows. We hope these revisions meet your satisfaction and make our manuscript acceptable for publication in your journal.

We look forward to hearing a positive response from you.

Best regards,

Diyan Li, Professor, Ph.D.
Address: Institute of Animal Genetics and Breeding, College of Animal Science and Technology, Sichuan Agricultural University, Chengdu 611130, China.
E-mail: diyanli@sicau.edu.cn

Detailed responses to reviewers:

All comments provided by reviewers are in black, and our responses are in red. This following text is also included in the uploaded file "Response letter":

Reviewer #1: Dear authors, the work described in the manuscript is very extensive! I have not yet seen an analysis of host genetics, microbiota composition and host transcriptomics coupled with egg production data. Also while not perfect the manuscript is written well.

Response: Thank you for your positive comments.

Main problems:

1. It is clear you found an association between specific bacteria and egg production. You have also shown that some form of inflammation is associated with the changes in microbiota and egg production. However, you assume that the bacteria affect inflammation which affect egg production. But other options also exist. For example, it is possible that waning egg production, from other causes, changes the conditions in the oviduct so that the microbiota would change. Or inflammation, for example because of a pathogen, might modulate both egg production as well as microbiota composition. Please change the text so that the readers understand you are speculating and briefly mention the other options.

Response: As suggested, we have added discussion of the other options affecting egg production in the "Results and Discussion" sections (Lines 355-363).

"Multiple factors, especially host species, potential pathogens, and immune status of the host, all play a major role in the female reproductive organs adversely interfering with the egg industry in laying flocks [53]. Additionally, the digestive tract environment

of low-egg producing hens is fragile and susceptible to the influence of exogenous microorganisms [41]. Pathogenic infection, room temperature fluctuations, management systems, and other sudden changes to various factors can alter the composition of microbiota [54, 55]. These alterations may cause a significant degradation in production performance. Here, our results indicate that the reproductive tract microbiota play an important role in egg production."

2. While it is too late to change, it is a pity that you did not characterize the cecum community. Of all of the intestinal communities the cecum is by far the biggest and the most likely to affect the nutrition of the hen, thereby possibly affecting egg production.
Response: Thank you for your comments and understanding. We have included some information regarding this in the Discussion (Lines 288-291).
"The cecum has distinct microbial community profiles [44-46] that were not explored in this study. Microbial community analysis of the cecum microbiota in chickens exhibiting different egg production performances requires further investigation."

3. Please make sure to reference and discuss relevant literature. I quickly identified Elokil A. A. Animal 2020 which performed a limited but similar analysis. Please perform a literature search and make sure to reference and discuss relevant work.
Response: Thank you for your helpful suggestions. As suggested, we have added and discussed additionally relevant literature references (Lines 280-288).
"In accordance with previous findings, there is clear evidence of the role of fecal microbiomes in low and high egg-laying performance in hens; Elokil et al [16] demonstrated a significantly positive association between the microbial genus *Lactobacillus* and egg-laying performance ($P < 0.05$). Likewise, Wang et al [41] reported that *Lactobacillus* was also abundant in the feces of high-yield hens. The genus *Lactobacillus* produces growth promoters and exhibits antimicrobial activity against pathogenic microbes [42, 43] which may explain why the increasing abundance of *Lactobacillus* in the high-yield group is beneficial to egg-laying performance."

Minor problems:
Lines 48-49 - something is wrong with this sentence.
Response: Thank you for the pointing this out. We have revised the sentence in the manuscript (Lines 46-48).
"An abnormal vaginal microbiota may predispose individuals to increased microbial invasion of the amniotic cavity and fetal damage [11, 12]."

Lines 67-69 - it is not clear what you are trying to say here.
Response: As commented, the sentence was not clear and had limited relevance to the manuscript so we deleted the sentence.

Line 71 - 16S rDNA sequencing and not whole-genome sequencing.
Response: In this study, we conducted whole-genome sequencing on 128 laying hens and 16S rDNA sequencing on 768 samples from six sites. We have revised the sentence

in the manuscript (Lines 68-71).
"Here, we performed 16S rDNA sequencing on 768 samples from three reproductive (vagina, uterus, and isthmus) and three digestive (crop, gizzard, and small intestine) tract sites and whole-genome sequencing of 128 laying hens."

Line 96 - please be more specific regarding which part of the small intestine was sampled.
Response: The jejunum of the small intestine was sampled. As suggested, we provide a more specific explanation of which part of the small intestine was sampled in the revised manuscript (Lines 420-421).
"A 12-cm-long fixed mid-region of the small intestine (jejunum) was collected from each bird."

Lines 100-108 - description of host DNA extraction is not appropriate another the heading "microbial genomic DNA extraction".
Response: "Microbial genomic DNA extraction" has been changed to "Host and microbial genomic DNA extraction" in the revised manuscript (Line 426).

Line 109 and elsewhere - 16S rDNA and not 16S rRNA.
Response: "16S rRNA" was changed to "16S rDNA" throughout the whole manuscript.

Line 154 - why are you using OTUs with 97% identity and not 100% identical amplicon sequence variants (ASVs)?
Response: As an alternative to OTUs, ASVs have been proposed as a way to adapt the thresholds suggested by genome sequencing to microbial community analysis using 16S rDNA sequences. Meanwhile, the OTU approach is still one of the primarily used methods for analyzing 16S rDNA-seq data (Dvergedal et al., 2020; Pinna et al., 2021; Wen et al., 2021). Schloss PD recently evaluated the clustering risk among ASV and OTU methods, and reached a conclusion that ASVs and the use of overly narrow thresholds to identify OTUs increase the risk of splitting a single genome into separate clusters (Schloss, 2021).

Since there is no consensus for a biological definition of a bacterial species (Sanford et al., 2021), microbiologists should accept that how bacterial species are named is biased and that taxonomic rules are not applied in a consistent manner. This makes it impossible to fit a distance threshold that matches a set of species names (Konstantinidis and Tiedje, 2005). Furthermore, the 16S rDNA sequence does not evolve at the same rate across all bacterial lineages (Schloss and Westcott, 2011), which limits the biological interpretation of a common OTU definition. A distance-based definition of a taxonomic unit based on the 16S rDNA or full-genome sequences is operational and not necessarily grounded in biological theory (Yarza et al., 2014; Barco et al., 2020). One benefit of a distance-based OTU definition is the ability to mask residual sequencing errors. The sequences generated in microbiome studies can harbor PCR and sequencing errors. These errors would only exacerbate the inflated number of ASVs.

Although there are multiple reasons why proponents favor ASVs, we feel the significant risk of artificially splitting genomes into separate clusters is too high to warrant their use.

References

Barco, R., G. Garrity, J. Scott, J. Amend, K. Nealson, and D. Emerson. 2020. A genus definition for bacteria and archaea based on a standard genome relatedness index. mBio 11:e02475-02419.

Dvergedal, H., S. R. Sandve, I. Angell, G. Klemetsdal, and K. Rudi. 2020. Association of gut microbiota with metabolism in juvenile Atlantic salmon. Microbiome 8:160.

Konstantinidis, K. T., and J. M. Tiedje. 2005. Towards a genome-based taxonomy for prokaryotes. Journal of Bacteriology 187:6258-6264.

Pinna, N. K., R. M. Anjana, S. Saxena, A. Dutta, V. Gnanaprakash, G. Rameshkumar, S. Aswath, S. Raghavan, C. S. S. Rani, and V. Radha. 2021. Trans-ethnic gut microbial signatures of prediabetic subjects from India and Denmark. Genome Medicine 13:36.

Sanford, R. A., K. G. Lloyd, K. T. Konstantinidis, and F. E. Löffler. 2021. Microbial taxonomy run amok. Trends in Microbiology 29:394-404.

Schloss, P. D. 2021. Amplicon sequence variants artificially split bacterial genomes into separate clusters. mSphere Jul 21:e0019121. doi: 10.1128/mSphere.00191-21.

Schloss, P. D., and S. L. Westcott. 2011. Assessing and improving methods used in operational taxonomic unit-based approaches for 16S rRNA gene sequence analysis. Applied and Environmental Microbiology 77:3219-3226.

Wen, C., W. Yan, C. Mai, Z. Duan, J. Zheng, C. Sun, and N. Yang. 2021. Joint contributions of the gut microbiota and host genetics to feed efficiency in chickens. Microbiome 9:126.

Yarza, P., P. Yilmaz, E. Pruesse, F. O. Glöckner, W. Ludwig, K.-H. Schleifer, W. B. Whitman, J. Euzéby, R. Amann, and R. Rosselló-Móra. 2014. Uniting the classification of cultured and uncultured bacteria and archaea using 16S rRNA gene sequences. Nature Reviews Microbiology 12:635-645.

Line 157 - what are "singleton OTUs"? OTUs with only one read? OTUs found in only one sample?

Response: "singleton OTUs" represent OTUs found in only one sample (Lines 483-484).

"Singleton OTUs (OTUs found in only one sample) that did not match the reference database were removed."

Lines 159-161 - why were low abundance OTUs discarded? Does it affect your analysis? In general, it is better not to modify the data base extensively. Specifically, some phylogenetic groups are represented by multiple low abundant strains with a high total abundance whereas other phylogenetic groups are represented by a single highly abundant strain. By getting rid of such lower abundance OTUs you might be creating an artifact.

Response: Thank you for the helpful comments. Errors introduced by next-generation amplicon sequencing tend to induce spurious OTUs and spurious counts in OTU tables,

both of which are especially prevalent at low abundances. Despite the power of NGS and the progress achieved, generated data is imperfect, being subject to different types of errors, including those inherent to PCR amplification (substitutions and chimeric sequence formation) and sequencing-specific biases which are characteristic of each sequencing technology. Sequencing errors are predominantly caused by base substitutions, although base deletions, low-quality reads, variable read lengths and non-target amplification are also known error sources which may result in false species affiliation. Undetected chimeric sequences, caused by the hybridization of DNA fragments from different species also reduce the reliability of the 16S rDNA sequence-based phylogenetic composition of microbial communities. Together, these different errors generate a high number of lower-abundance sequences, which lead to overestimations of actual community diversity and the creation of many false taxa. Those spurious sequences are hard to filter out using current mainstream pipelines implementing error correction, denoising, and stringent filtration of chimeric sequences, contaminants and non-bacterial contents. Previous study have reported that although the overall abundance of these pseudo sequences was low, introducing them into analysis increased the total number of features to 10 times higher than expected and enlarged the divergence of the alpha and beta diversity analyses among the different methods (Wang et al., 2018). Lower-abundance and lower-quality sequences were observed to surround higher-abundance, biologically real sequences, forming error clouds (Bokulich et al., 2013; Edgar, 2013). Various researchers have developed different approaches to remove these pseudo sequences.

In our work, we described the numerical distribution of OTUs with different existing ratios in samples, after which the slope fluctuation is estimated. We considered there are a lot of false positives for these microbiotas when fluctuations are great. Therefore, we selected OTUs that existed in more than 20% of samples according to the slope distribution curve (**Supplementary Fig. S1a, b**). This filtering rule will improve the stability and accuracy of further analyses. Similar filtering rules have also been reported in previous analyses (Zierer et al., 2018). In addition, we have cited **Supplementary Fig. S1a, b** in the revised manuscript (Lines 488-489).

References

Bokulich, N. A., S. Subramanian, J. J. Faith, D. Gevers, J. I. Gordon, R. Knight, D. A. Mills, and J. G. Caporaso. 2013. Quality-filtering vastly improves diversity estimates from Illumina amplicon sequencing. Nature Methods 10:57-59.

Edgar, R. C. 2013. UPARSE: highly accurate OTU sequences from microbial amplicon reads. Nature Methods 10:996-998.

Wang, J., Q. Zhang, G. Wu, C. Zhang, M. Zhang, and L. Zhao. 2018. Minimizing spurious features in 16S rRNA gene amplicon sequencing. PeerJ Preprints 6:e26872v1 https://doi.org/10.7287/peerj.preprints.26872v1

Zierer, J., M. A. Jackson, G. Kastenmüller, M. Mangino, T. Long, A. Telenti, R. P. Mohney, K. S. Small, J. T. Bell, and C. J. Steves. 2018. The fecal metabolome as a functional readout of the gut microbiome. Nature Genetics 50:790-795.

Lines 378-382 - this is an interesting idea that you did not establish well. Could you determine unifrac (or any other metric) distances of samples from different organs. i.e. compare all vagina samples to all small intestine samples and determine average Unifrac distances and stDev, and do so for each pair of organ sites. Perhaps you will gain a new figure showing how similar or different are the communities in each site.
Response: We are grateful for the suggestion. As suggested by the reviewer, we added a new figure (**Supplementary Fig. S2j**) showing the weighted UniFrac distances among the six sites.

Line 387 - cyanobacteria are not likely gut or reproductive tract inhabitants. This is more likely an artifact of identifying feed derived chloroplast DNA. You might want to mention this.
Response: Thanks for your helpful suggestions. The chickens used in our study are fed using corn-soybean-based diets, so the *Cyanobacteria* (likely an artifact of feed-derived chloroplast DNA) were misidentified as microbes. Additionally, we have revised the sentence in the revised manuscript (Lines 118-120).
"*Firmicutes*, *Proteobacteria*, and *Cyanobacteria* (likely an artifact of feed-derived chloroplast DNA) accounted for 71.45% - 97.86% of all OTUs."

Lines 398-402 - what is the connection between the "well-documented protective role of lactobacillus…" and differences in the levels of lactobacillus between gut and reproductive tract? Does the reproductive tract not deserve protection? Please stick to the data. Do not attach unsupported conclusions. Instead connect the levels of Lactobacillus and pH.
Response: As suggested, we have connected the levels of *Lactobacillus* and pH in the revised manuscript (Lines 134-139).
"*Lactobacillus* is thought to inhibit pathogenic bacteria by lowering the environmental pH through lactic acid and hydrogen peroxide production [24]. This genus was highly abundant in the digestive tracts which were characterized by low pH values which strongly limits the growth of most pathogens [25, 26]. In contrast, *Lactobacillus* was less abundant in the reproductive tract where an alkaline pH is needed to maintain sperm motility [27, 28]."

Lines 407-411 - if I understand correctly, you are implying a connection possibly caused because of the flow of material from different sites. You may want to note that.
Response: Thank you for the helpful comments. Yes, we want to note that, and we have added a sentence in our revised manuscript (Lines 146-147).
"These results imply there is a connection of microbiome communities possibly caused because by the flow of material from different sites."

Line 419-421 - you have reduced a whole field of study to just one "previous study". And in any case I am not sure what you wanted to write here.
Response: We have now included additional references in the revised manuscript (Line 158).

Line 442 - not chloroplast and mitochondria-like microbes but rather true chloroplasts and mitochondria which you misidentified as microbes.

Response: Thanks for your helpful suggestion. We have revised the sentence in the manuscript (Lines 179-180).

"*Unidentified Chloroplast* (2.94%) and mitochondria-like (2.37%) materials from plant consumption."

Line 447 - does Helicobactor maintain near natural pH and a microaerophilic environment? Is that its role in the gut? Please do not bring up bits and pieces of information if this is not really relevant to your results. What is important is that a possible pathogen was found in your chickens.

Response: Thanks for the comments. In order to infect the gastric mucosa, *Helicobacter pylori* has to survive in the gastric acidic pH, and *Helicobacter pylori* has well developed mechanisms to neutralize the effects of acidic pH (Ansari, S. and Y. Yamaoka. 2017. Survival of *Helicobacter pylori* in gastric acidic territory, helicobacter 22:e12386), that is its role in the gastric tract. In addition, we have reorganized the sentence in the revised manuscript (Lines 183-185).

"As a possible pathogen, *Helicobacter* specifically inhabits the small intestine in chickens, and may be involved in inflammation, metabolism, and neutralization of gastric acid [35-37]."

Lines 450-463 - this whole paragraph contains a lot of disjoined bits and pieces of information. Does it really matter that some bacteria were changed in immune suppressed honey bees? What are you trying to say? Consider taking this whole paragraph off.

Response: Thanks for the comment. This whole paragraph mainly displays the results of the site-associated bacterial taxa in the three reproductive tract sites identified by LEfSe and discussed association with the previous literature. Additionally, we have deleted the disjoined bits and pieces of information and reorganized the sentence in the paragraph in our revised manuscript (Lines 186-194).

"*Unidentified Erysipelotrichaceae* showed higher abundance in the three reproductive tract sites (1.83% - 2.40%). Bacteria associated with the isthmus and uterus both showed higher abundances than in the other sites. Several genera (typically, *Romboutsia*, *Fusobacterium*, and *Clostridium sensu stricto 1*) were dominant in the vagina (> 25% of the microbiota) but had lower abundances in the other sites (**Fig. 2b**). Among these, vaginal *Romboutsia* could be employed as a predictor for egg number in laying hens [8]. Six *Bacteroidetes* bacterial taxa were isthmus-associated; *Bacteroides* species live on host mucus-secreted polysaccharides and this flexible foraging behavior contributes to diversity and stability [38]."

Lines 464-466 - do your results support this speculation? If yes please expend, if not please remove.

Response: Thank you for the suggestion. Lines 464-466 have been removed from the

revised manuscript.

Line 492 - 0.21% of total abundance? Of total species?

Response: Yes, it is "of total species". We have reorganized the sentence and clarified in the manuscript (Line 223).

"accounting for 0.21% of the microbiota species tested in the small intestine".

Lines 560-568 - this is a good example of discussion! Here you brought relevant information and created a data based speculation.

Response: Thank you for your positive remarks about this discussion.

Line 646 - what do you mean in "microbiome succession"?

Response: "microbiome succession" has been changed to "microbial community" in the revised manuscript (Line 390).

Supplementary figure 2 contains two separate topics - functional sequencing data and bacterial community analysis. Please divide into separate figures.

Response: As suggested, we have divided Supplementary figure 2 into two separate figures (**Supplementary Fig. S2 and S11**).

Reviewer #2: This is a very good study to which I have only a few comments, mostly to microbiota part in which I am stronger than in chicken genetics. The only weaker part, but not weak, is the fact that the study was performed with only a single flock and was not repeated with different hens at all. I understand that it is impossible to repeat the study in another flock but for example you could have set up Cl. leptum PCR and check for its presence in reproductive tract in completely different birds.

Response: We appreciate the positive feedback and sincerely thank the reviewer for the thoughtful and supportive recommendations, which are of great help in improving the quality of this manuscript.

Please consider the following points.

line 94, indeed laparotomy?

Response: "After laparotomy" has been changed to "After the abdomen was opened" in the revised manuscript (Line 417).

l.111 and 114, I do not understand wha PCR kit you used. Please, reword.

Response: The PCR amplification was performed using Phusion® High-Fidelity PCR Master Mix (New England Biolabs, Ipswich, Ma, USA). We have revised the sentence in the revised manuscript (Lines 436-439).

"The V4 hypervariable region of the bacterial 16S rDNA was amplified using Phusion® High-Fidelity PCR Master Mix (New England Biolabs, Ipswich, Ma, USA) and the universal primers 515 F (5′-GTGCCAGCMGCCGCGGTAA-3′) and 806 R (5′-GGACTACHVGGGTWTCTAAT -3′) [56]."

l.253, 254, this sentence is somewhat compromised. Please check and reword.
Response: Thank you for the comments, and we have revised the sentence in the revised manuscript (Lines 578-579).
"In addition, we further removed the SNPs with adjacent distances ≤ 5."

l.342, rather verified, or confirmed, then detected. By the way, I am not sure whether you used data from the qPCR in the rest of the manuscript
Response: We have changed "detected" to "verified" in the revised manuscript (Line 664). We only used qPCR data in the "RNA sequencing (RNA-seq) analysis" section.

l.388, these are not Cyanobacteria, this is chloroplast DNA from plants in the feed, you may check very recent paper Volf et al. Eggshell and Feed Microbiota Do Not Represent Major Sources of Gut Anaerobes for Chickens in Commercial Production. Microorganisms 2021, 9, 1480.
Response: Thanks for the valuable suggestions, and we have read the above reference carefully. The chickens used in our study are fed using corn-soybean-based diets, so the *Cyanobacteria* (likely an artifact of feed derived-chloroplast DNA) was misidentified as microbes. Additionally, we have revised the sentence in the manuscript (Lines 121-122).
"*Cyanobacteria* (likely an artifact of feed-derived chloroplast DNA) was the dominant material in the gizzard (48.19% of the total abundance)."

l.462,463, I would suggest alternative explanation in this case and this is living on expense of host mucus secreted polysaccharides, Sonnenburg, J. L. et al. Glycan foraging in vivo by an intestine-adapted bacterial symbiont. Science 307, 1955-1959 (2005)
Response: As suggested, we have cited the reference and revised the sentence in the manuscript (Lines 192-194).
"*Bacteroides* species live on host mucus-secreted polysaccharides and this flexible foraging behavior contributes to diversity and stability [38]."

l.559, check that B. fragilis might be a consequence of intensive human care, Kollarcikova et al. Different Bacteroides Species Colonise Human and Chicken Intestinal Tract. Microorganisms 2020, 8, 1483.
Response: We are grateful for the suggestion. We have cited the useful and interesting reference in the revised manuscript (Lines 301-303).
"Interestingly, a recent study reported that the human-adapted *Bacteroides* species are likely introduced to chicken flocks by contact with humans and can temporarily persist in chickens [49]."

l.615, how can you know this? What if all of this the other way round, and I indeed believe that this is the other way, i.e. hens becomes of compromised performance, due to whatever factor, within but possibly also outside of those which you have monitored. This naturally results in decrease in egg lay but also in increased inflammatory response.

Locally changed conditions due to inflammatory signaling change, infiltrating heterophils and macrophages produce antimicrobial peptides and reactive oxygen species and strict anaerobes will be the firs bacterial species to decrease in a response of increasing oxygen concentration. What is cause and what is consequence. I do not know, I think that you do not know either, though you blame bacteria that these are responsible for the response.
Response: Thank you for the comments. We deleted the sentence that caused misunderstanding.

l.631, similar to previous comment, I do not think that there is any downregulation. In high egg producers, there is basal, background expression of inflammatory marker genes. And these are induced in the hens with compromised performance. Be also careful, whether this induction since this could also be a cause of infiltration of macrophage with their specific expression profile, and you then mistakenly conclude on induction when purifying mRNA from a total complex tissue.
Response: Thanks for your comments. "downregulated" has been changed to "different expressed" in the revised manuscript (Line 383).

l.656, the same as above, be careful what is cause and what is consequence. Increase in reactive oxygen species may affect the most strict anaerobes. When these are present, there is no inflammation. When these are eliminated by increase in oxygen concentration and all other inflammatory responses, this is explained that these bacteria are anti-inflammatory. These are not, these only dislike inflammation and oxygen.
Response: Thanks for your helpful suggestion. We have deleted the sentence in the revised manuscript.
