# Supplementary material for: Association of female reproductive tract microbiota with egg production in layer chickens
Source: Gigascience. 2021 Sep 23;10(9):giab067. doi: 10.1093/gigascience/giab067 (PMC8460357; doi:10.1093/gigascience/giab067)
Supplement: giab067_Supplemental_Figures_and_Tables [file giab067_supplemental_figures_and_tables.zip › Supplementary Figures.docx]

**Supplementary Material**


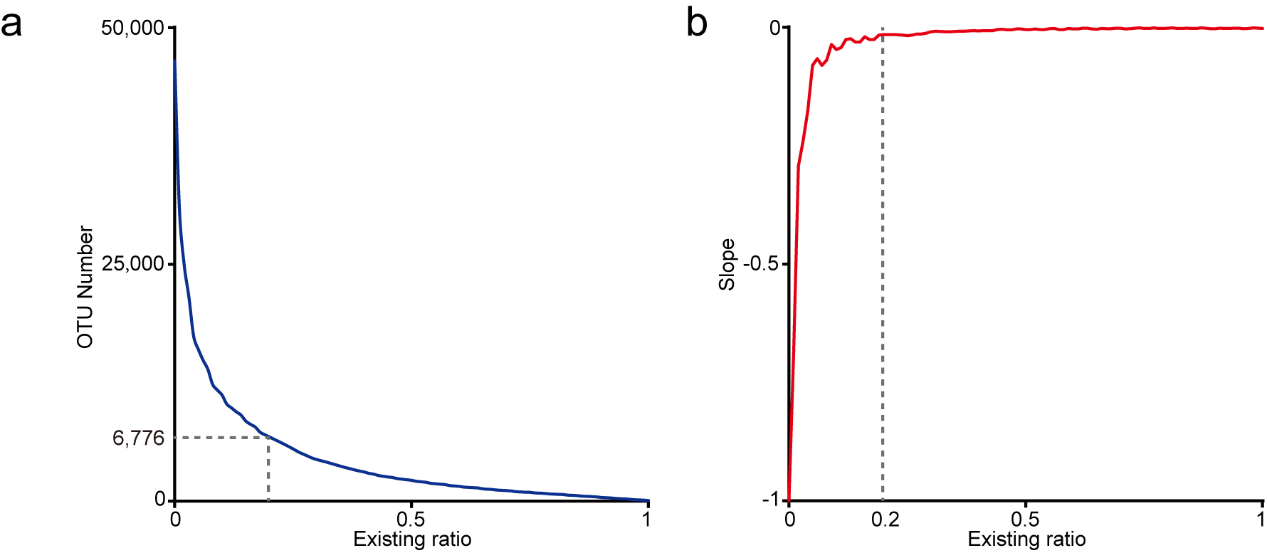


**Supplementary Figure S1.** The number distribution of OTUs with different existing ratio of samples. (**a**) The number plot of OTUs with different existing ratio of samples. (**b**) The relationship of existing ratio and the slope of the curve in **a**. The dotted line indicated the threshold utilized to remove existing ratio distribution trend due to fluctuate greatly.

**
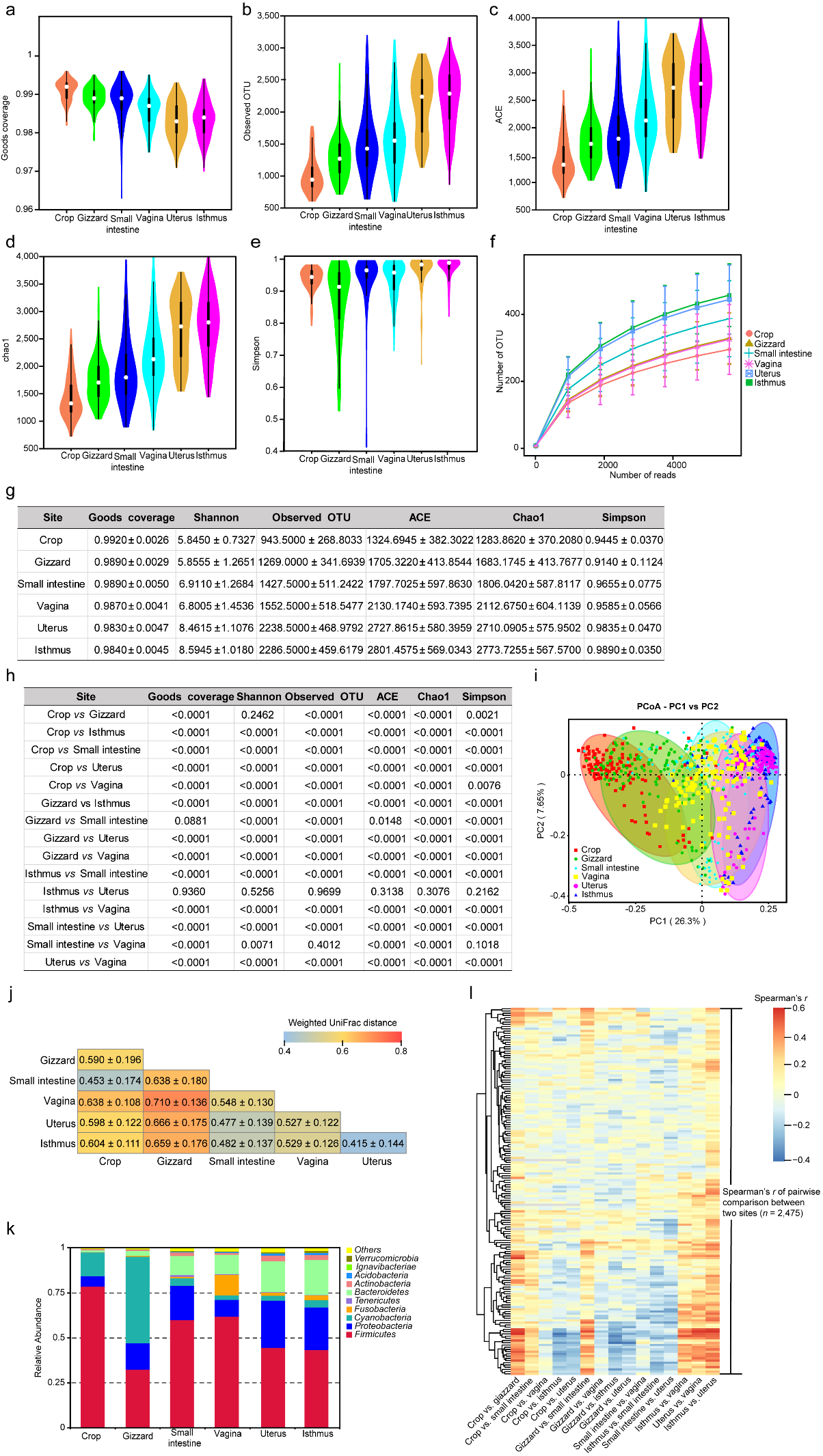
**

**Supplementary Figure S2.** Alpha diversity, relative abundance, and Spearman’s *r* values of specific microbiota among the six sites. (**a-e**) Alpha diversity comparison based on Good’s coverage, observed OTU，ACE, Chao1, and Simpson indices, using Wilcoxon rank-sum test to determine signiﬁcant differences. (**f**) Rarefaction curves of observed OTU. (**g**) Alpha diversity values of the six sites. Values are represented as median ± SD. (**h**) *P* values of Wilcoxon rank-sum test of each comparison for six alpha diversity indices. (**i**) PCoA of the 768 samples based on unweighted UniFrac distances. (**j**) Beta diversity comparison based on the weighted UniFrac distances among the six sites. The values are ﬁlled with weighted UniFrac distances (mean ± SD) in the corresponding comparisons. All comparisons were significantly different using Wilcoxon rank-sum test (*P* < 0.05). (**k**) Relative abundance of the top ten dominant microbial phyla in the six sites. (**l**) Only microbial genera that were present in at least 461 samples (60% of the total) were plotted. Each row represents a microorganism. Among 2,475 Spearman’s *r* values, only 362 (14.62%) were significantly correlated (*P* < 0.05).


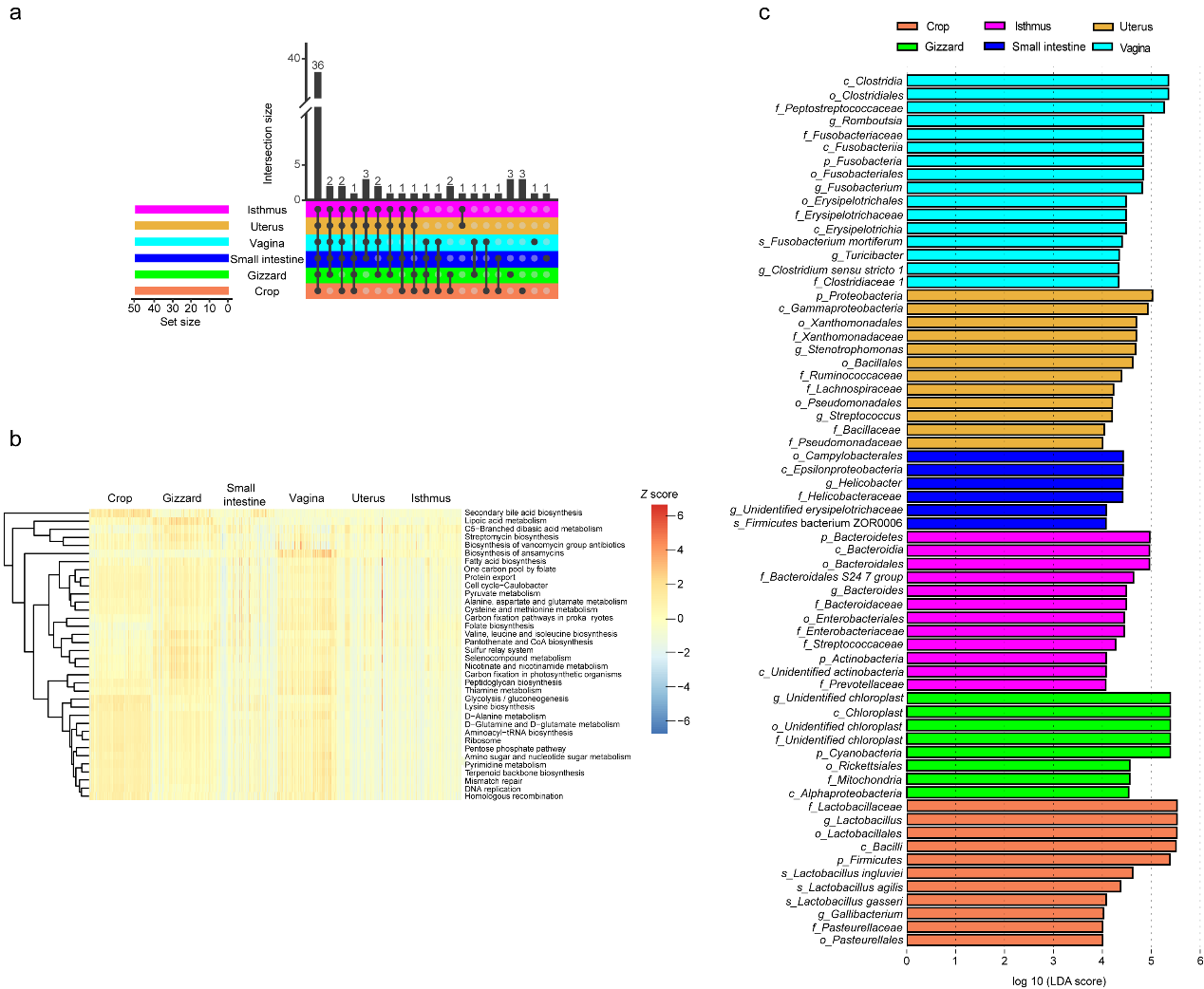


**Supplementary Figure S3.** Comparison of the functional capacities of the reproductive and digestive microbial communities among the six sites. (**a**) Overlap of the top 50 predictions among the six sites. (**b**) Heatmap showing the 36 overlapped predictions with different abundances among the six sites. The heatmap is color-coded based on row *Z* scores. (**c**) Map showing 65 site-associated bacterial taxa identified by LEfSe (LDA score > 4) in the test trial.

**
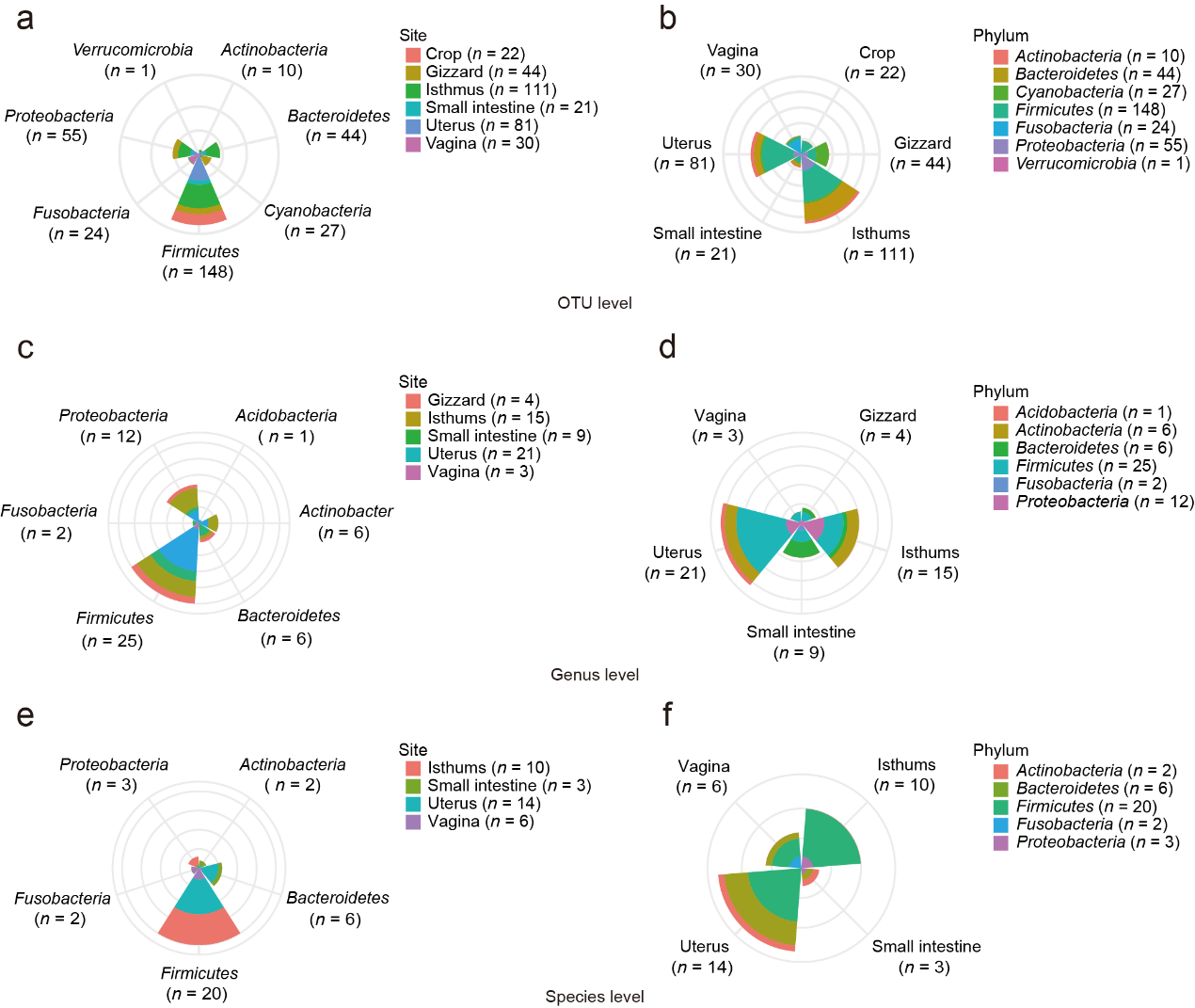
**

**Supplementary Figure S4.** Signiﬁcantly heritable microorganisms. The number of signiﬁcantly heritable microorganism OTUs, genera, and species (*P* < 0.05) grouped by sampling phyla (**a, c, e**) and site (**b, d, f**).


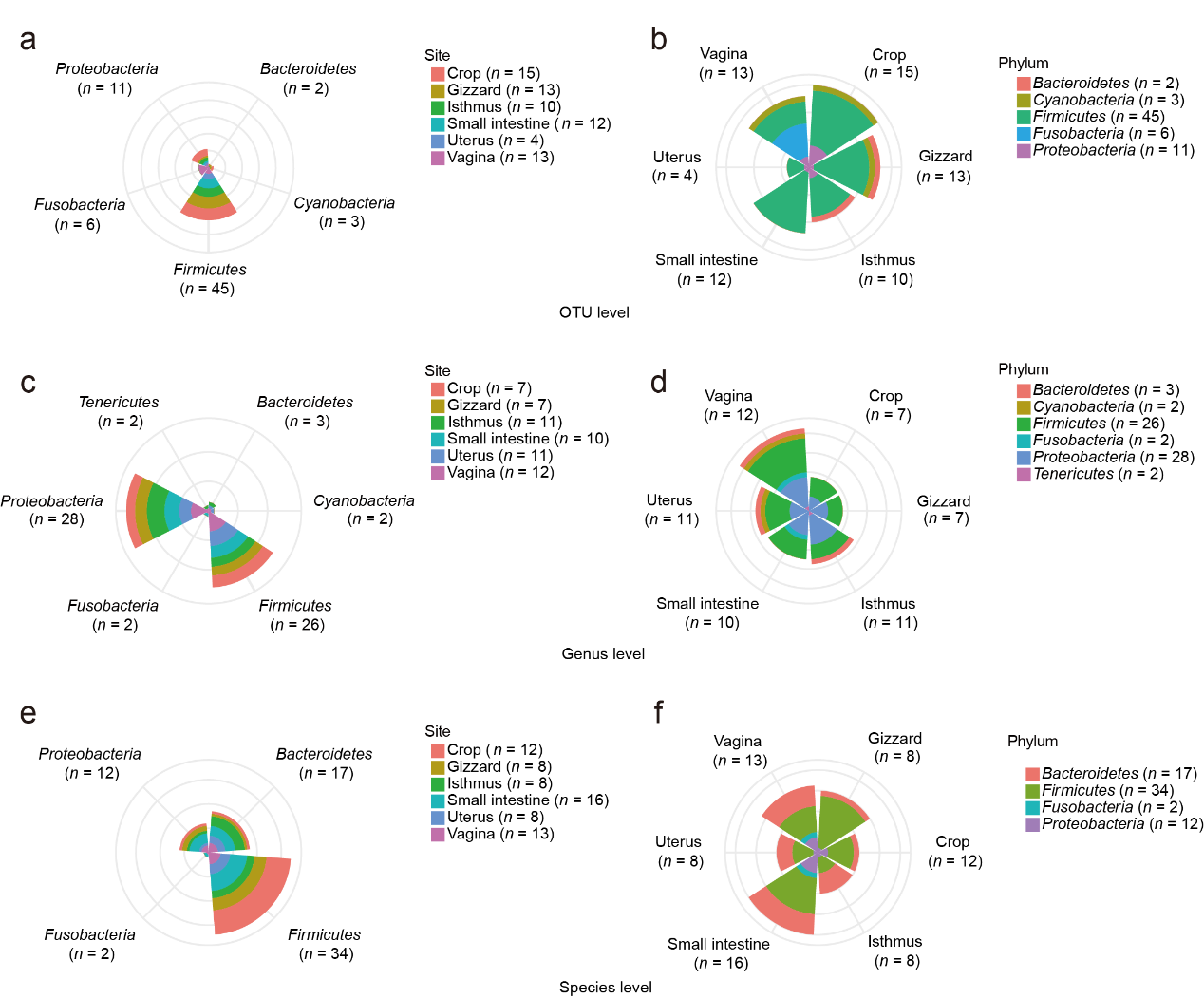


**Supplementary Figure S5.** Signiﬁcantly EN300-associated microorganisms. The number of microorganisms signiﬁcantly associated with EN300 detected at OTU, genus, and species (*P* < 0.05) levels grouped by sampling phyla (**a, c, e**) and site (**b, d, f**).


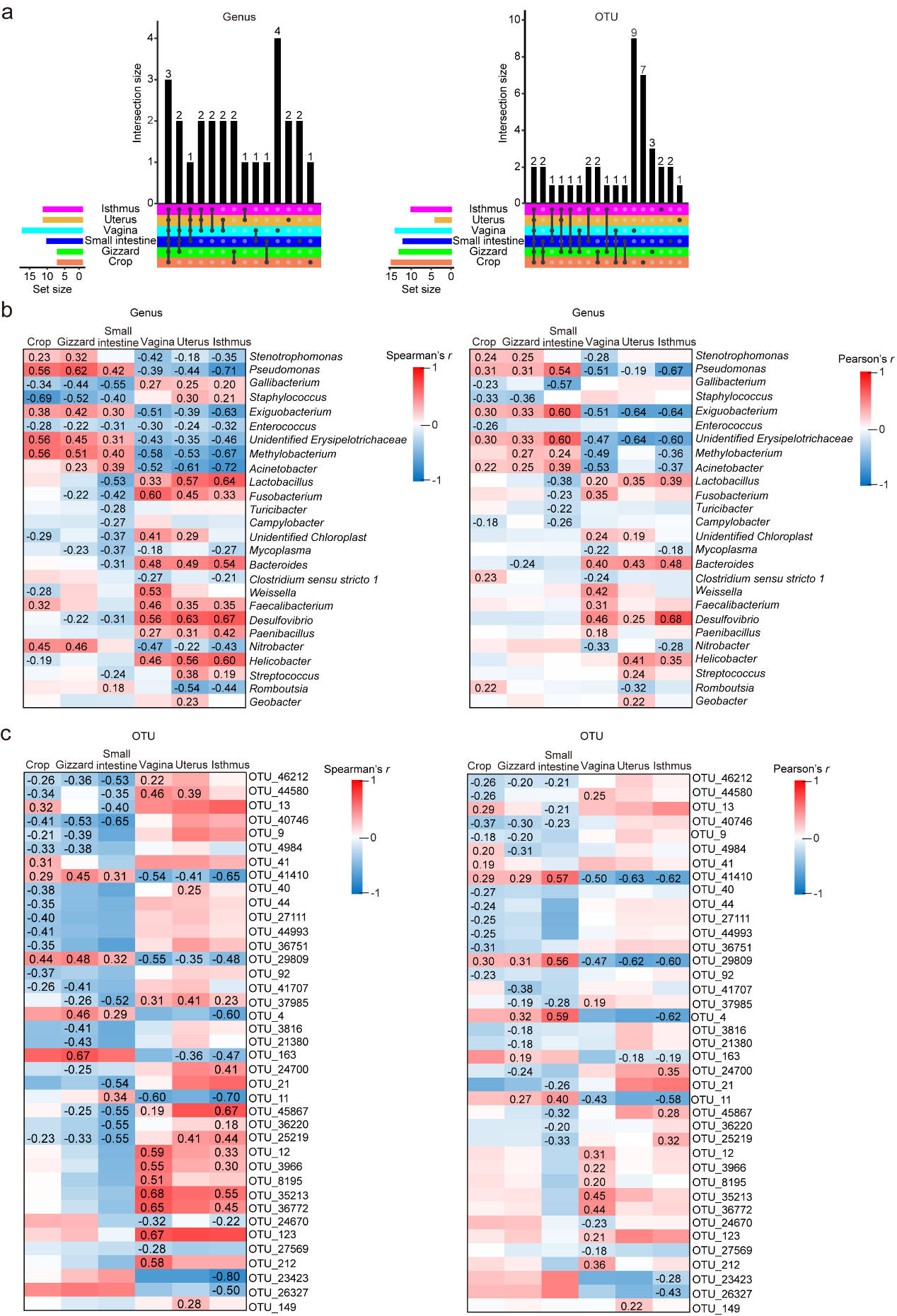


**Supplementary Figure S6.** EN300-associated microorganisms. (**a**) The number of microbial genera (left) and OTUs (right) associated with EN300 at *P* < 0.05 of three test methods of six sites and their overlap. (**b-c**) Pearson’s and Spearman’s *r* values between EN300 and EN300-associated 26 genera and 39 OTUs. Red and blue tiles indicate positive and negative correlations, respectively. Signiﬁcant *r* values are ﬁlled in numerically (*P* < 0.05).

**
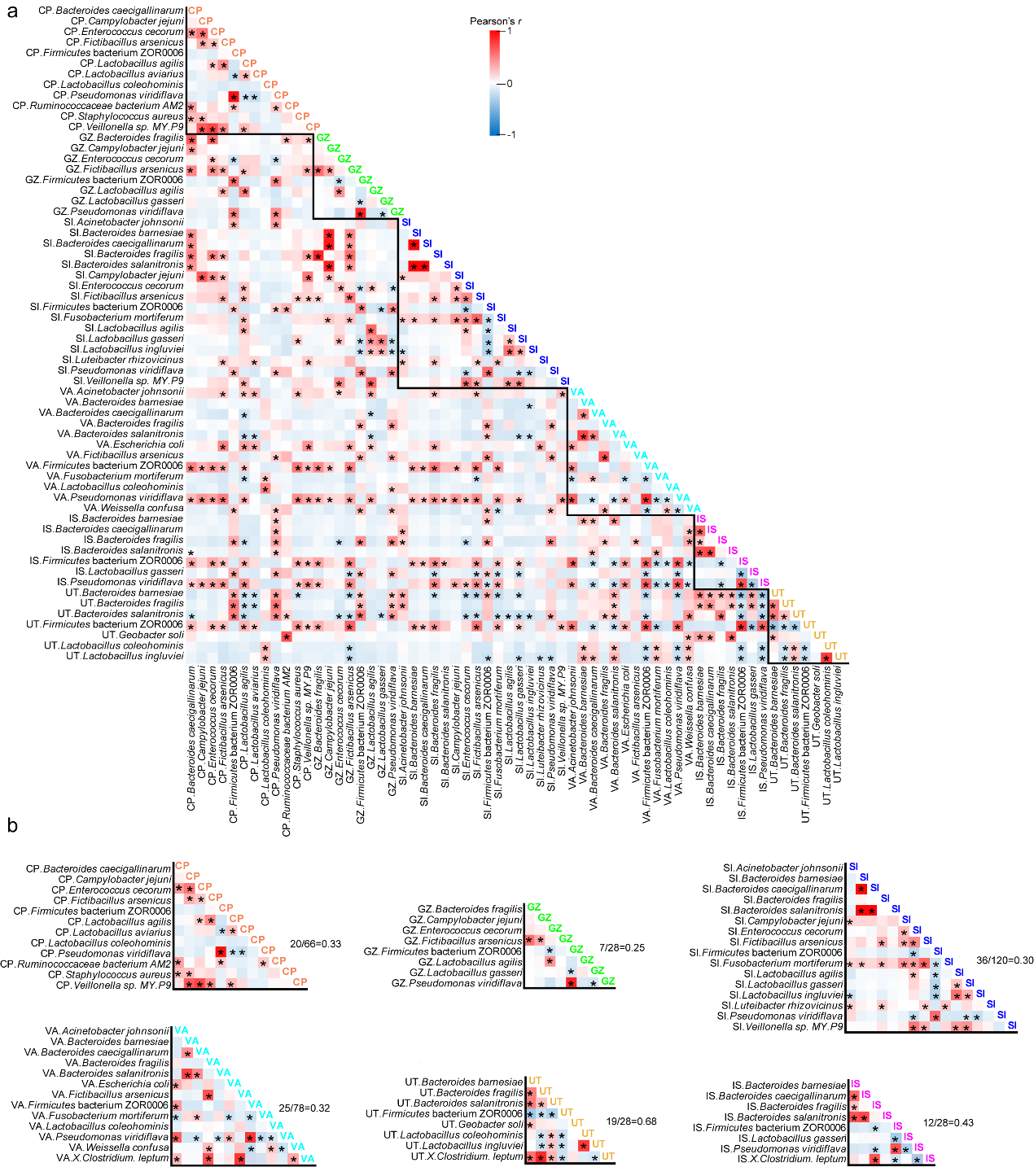
**

**Supplementary Figure S7.** Pearson correlations between EN300 and EN300-associated microorganisms. (**a**) Pearson’ s *r* values of candidate microbial species in the six sites. (**b**) Pearson’ s *r* values among microbial species in each site. CP: Crop, GZ: Gizzard, SI: small intestine, UT: uterus, IS: Isthmus, VA: vagina. Red and blue tiles indicate positive and negative correlations, respectively. The ratios on the right side of each site represents the number of significant correlations. **P* < 0.05.


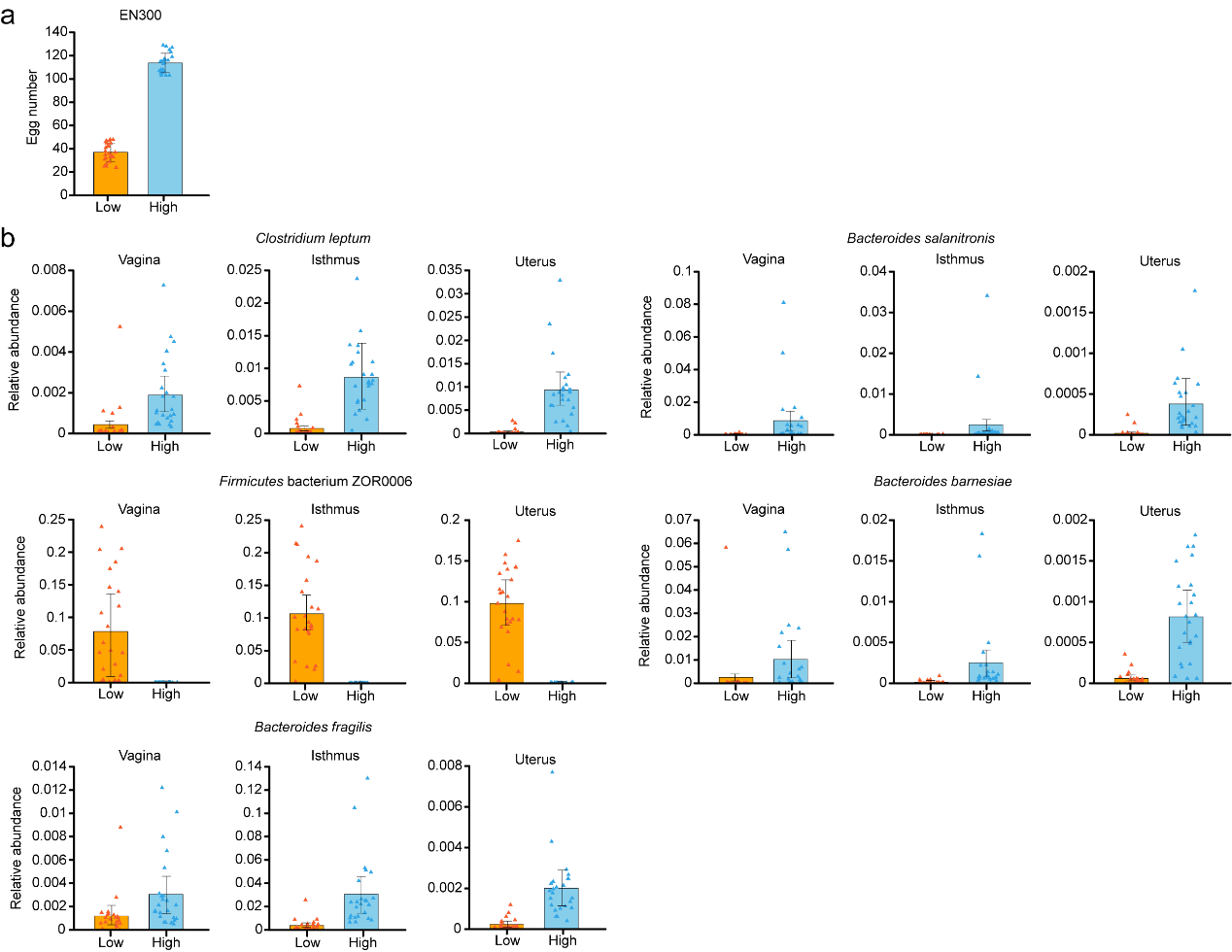


**Supplementary Figure S8.** Differences in the relative abundance of five species between the 20% of chickens with the highest and lowest egg production (EN300). (**a**) EN300 values for the 20% of individuals with the highest and lowest egg production. (**b**) EN300 values for the 20% of individuals with the highest and lowest abundances of *Clostridium leptum*, *Bacteroides salanitronis*, *Firmicutes* bacterium ZOR0006, *B. barnesiae,* and *B. fragilis* in the three reproductive tract sites. All comparisons were significantly different, established at *P* < 0.05.


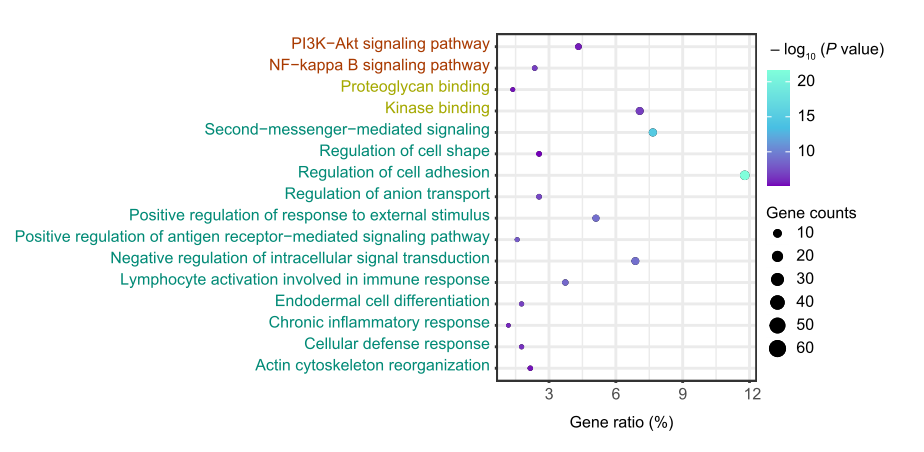


**Supplementary Figure S9.** Top 20 functional categories enriched by 1,051 genes exhibited significant expression changes between groups with the high- and low-egg production. The enrichment analysis was performed using the Metascape tool (See Method). GO-BP: biological process (blue), GO-MF: molecular function (yellow) and KEGG (red).

**
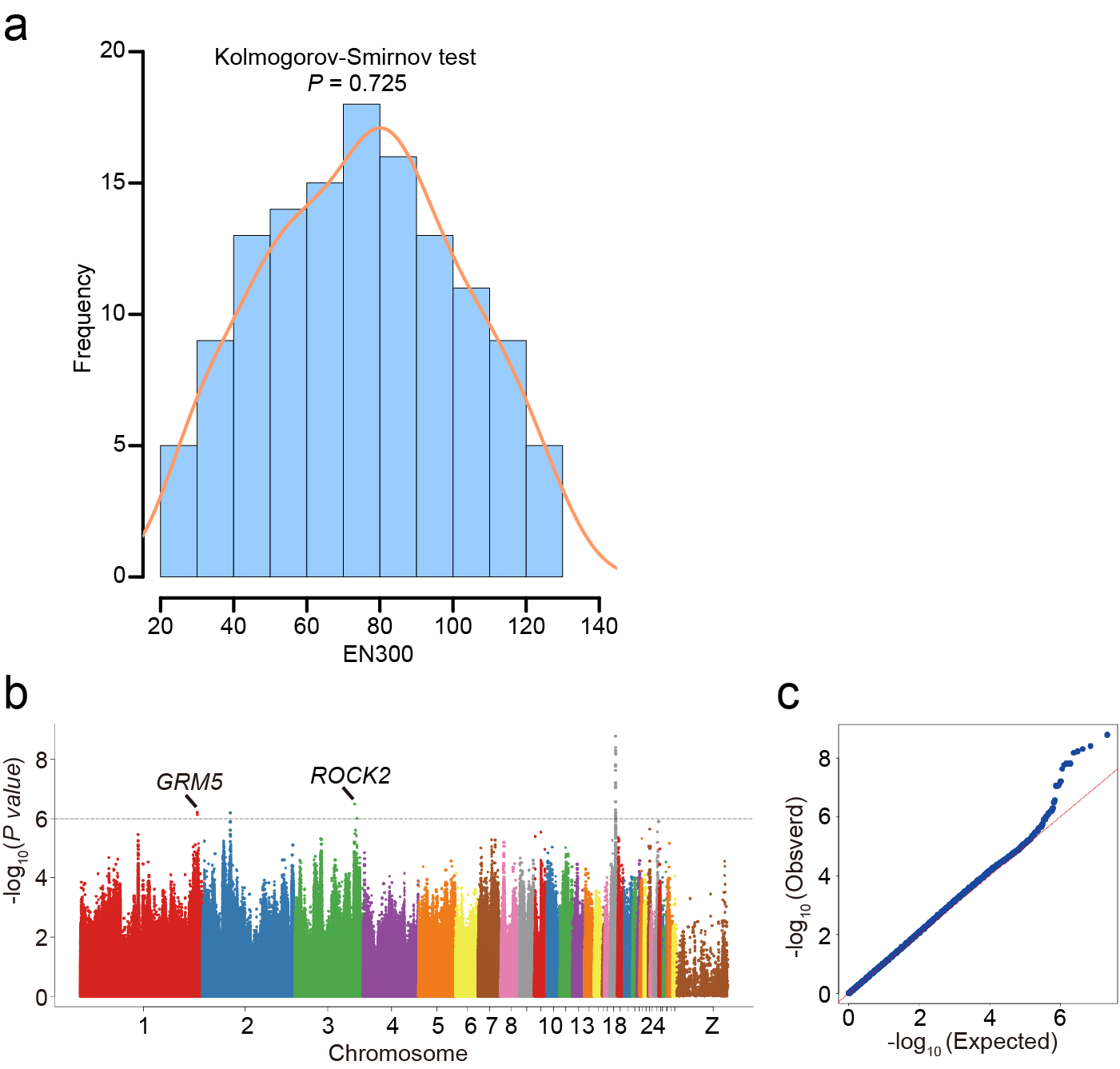
**

**Supplementary Figure S10.** Distribution of egg number at 300 days of age (EN300). Compared to the 20% of hens with the highest EN300 values, the 20% of hens with the lowest EN300 values exhibited a later start laying age, an earlier stop aging day and irregular lay performance.

**
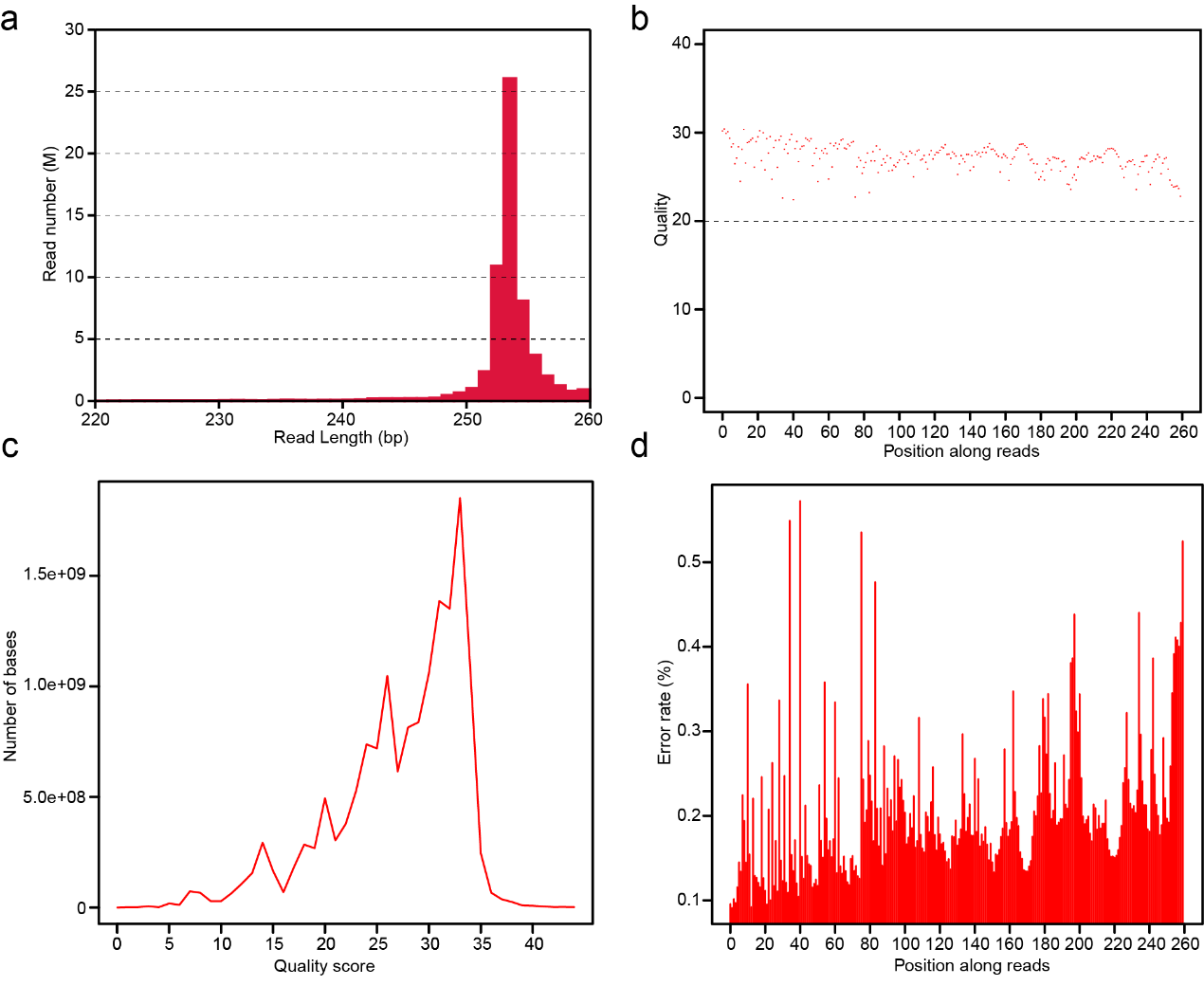
**

**Supplementary Figure S11.** Quality assessment of sequencing data. (**a**) Length distribution of reads. (**b**) Quality score of each base. (**c**) Quality score distribution of sequencing data. (**d**) Error rate distribution of reads.
