# Supplementary material for: Association of female reproductive tract microbiota with egg production in layer chickens
Source: Gigascience. 2021 Sep 23;10(9):giab067. doi: 10.1093/gigascience/giab067 (PMC8460357; doi:10.1093/gigascience/giab067)
Supplement: giab067_Response_to_Reviewer_Comments_Original_Submission [file giab067_response_to_reviewer_comments_original_submission.pdf]

Author's Response To Reviewer Comments

Close

Dear editor and reviewers,

Thank you very much for reviewing our manuscript “Female reproductive tract microbiota influence egg production in layer chickens” (ID: GIGA-D-21-00132) for possible publication in GigaScience. We sincerely thank the editor and two reviewers for their valuable feedback that we have used to improve the quality of our manuscript. According to the reviewers’ comments, we have made the relevant modifications to our manuscript. All modifications are marked in red in the revised manuscript and a point-by-point response to the reviewers’ comments follows. We hope these revisions meet your satisfaction and make our manuscript acceptable for publication in your journal.

We look forward to hearing a positive response from you.

Best regards,
Diyan Li, Professor, Ph.D.
Address: Institute of Animal Genetics and Breeding, College of Animal Science and Technology, Sichuan Agricultural University, Chengdu 611130, China.
E-mail: diyanli@sicau.edu.cn

Detailed responses to reviewers:
All comments provided by reviewers are in black, and our responses are in red. This following text is also included in the uploaded file "Response letter":

Reviewer #1: Dear authors, the work described in the manuscript is very extensive! I have not yet seen an analysis of host genetics, microbiota composition and host transcriptomics coupled with egg production data. Also while not perfect the manuscript is written well.
Response: Thank you for your positive comments.

Main problems:
1. It is clear you found an association between specific bacteria and egg production. You have also shown that some form of inflammation is associated with the changes in microbiota and egg production. However, you assume that the bacteria affect inflammation which affect egg production. But other options also exist. For example, it is possible that waning egg production, from other causes, changes the conditions in the oviduct so that the microbiota would change. Or inflammation, for example because of a pathogen, might modulate both egg production as well as microbiota composition. Please change the text so that the readers understand you are speculating and briefly mention the other options.
Response: As suggested, we have added discussion of the other options affecting egg production in the "Results and Discussion" sections (Lines 355-363).
"Multiple factors, especially host species, potential pathogens, and immune status of the host, all play a major role in the female reproductive organs adversely interfering with the egg industry in laying flocks [53]. Additionally, the digestive tract environment of low-egg producing hens is fragile and susceptible to the influence of exogenous microorganisms [41]. Pathogenic infection, room temperature fluctuations, management systems, and other sudden changes to various factors can alter the composition of microbiota [54, 55]. These alterations may cause a significant degradation in production performance. Here, our results indicate that the reproductive tract microbiota play an important role in egg production."

2. While it is too late to change, it is a pity that you did not characterize the cecum community. Of all of the intestinal communities the cecum is by far the biggest and the most likely to affect the nutrition of the hen, thereby possibly affecting egg production.
Response: Thank you for your comments and understanding. We have included some information regarding this in the Discussion (Lines 288-291).
"The cecum has distinct microbial community profiles [44-46] that were not explored in this study. Microbial community analysis of the cecum microbiota in chickens exhibiting different egg production

performances requires further investigation."

3. Please make sure to reference and discuss relevant literature. I quickly identified Elokil A. A. Animal 2020 which performed a limited but similar analysis. Please perform a literature search and make sure to reference and discuss relevant work.
Response: Thank you for your helpful suggestions. As suggested, we have added and discussed additionally relevant literature references (Lines 280-288).
"In accordance with previous findings, there is clear evidence of the role of fecal microbiomes in low and high egg-laying performance in hens; Elokil et al [16] demonstrated a significantly positive association between the microbial genus Lactobacillus and egg-laying performance ($P < 0.05$). Likewise, Wang et al [41] reported that Lactobacillus was also abundant in the feces of high-yield hens. The genus Lactobacillus produces growth promoters and exhibits antimicrobial activity against pathogenic microbes [42, 43] which may explain why the increasing abundance of Lactobacillus in the high-yield group is beneficial to egg-laying performance."

Minor problems:
Lines 48-49 - something is wrong with this sentence.
Response: Thank you for the pointing this out. We have revised the sentence in the manuscript (Lines 46-48).
"An abnormal vaginal microbiota may predispose individuals to increased microbial invasion of the amniotic cavity and fetal damage [11, 12]."

Lines 67-69 - it is not clear what you are trying to say here.
Response: As commented, the sentence was not clear and had limited relevance to the manuscript so we deleted the sentence.

Line 71 - 16S rDNA sequencing and not whole-genome sequencing.
Response: In this study, we conducted whole-genome sequencing on 128 laying hens and 16S rDNA sequencing on 768 samples from six sites. We have revised the sentence in the manuscript (Lines 68-71).
"Here, we performed 16S rDNA sequencing on 768 samples from three reproductive (vagina, uterus, and isthmus) and three digestive (crop, gizzard, and small intestine) tract sites and whole-genome sequencing of 128 laying hens."

Line 96 - please be more specific regarding which part of the small intestine was sampled.
Response: The jejunum of the small intestine was sampled. As suggested, we provide a more specific explanation of which part of the small intestine was sampled in the revised manuscript (Lines 420-421).
"A 12-cm-long fixed mid-region of the small intestine (jejunum) was collected from each bird."

Lines 100-108 - description of host DNA extraction is not appropriate another the heading "microbial genomic DNA extraction".
Response: "Microbial genomic DNA extraction" has been changed to "Host and microbial genomic DNA extraction" in the revised manuscript (Line 426).

Line 109 and elsewhere - 16S rDNA and not 16S rRNA.
Response: "16S rRNA" was changed to "16S rDNA" throughout the whole manuscript.

Line 154 - why are you using OTUs with 97% identity and not 100% identical amplicon sequence variants (ASVs)?
Response: As an alternative to OTUs, ASVs have been proposed as a way to adapt the thresholds suggested by genome sequencing to microbial community analysis using 16S rDNA sequences. Meanwhile, the OTU approach is still one of the primarily used methods for analyzing 16S rDNA-seq data (Dvergedal et al., 2020; Pinna et al., 2021; Wen et al., 2021). Schloss PD recently evaluated the clustering risk among ASV and OTU methods, and reached a conclusion that ASVs and the use of overly narrow thresholds to identify OTUs increase the risk of splitting a single genome into separate clusters (Schloss, 2021).
Since there is no consensus for a biological definition of a bacterial species (Sanford et al., 2021), microbiologists should accept that how bacterial species are named is biased and that taxonomic rules are not applied in a consistent manner. This makes it impossible to fit a distance threshold that matches a set of species names (Konstantinidis and Tiedje, 2005). Furthermore, the 16S rDNA sequence does not evolve at the same rate across all bacterial lineages (Schloss and Westcott, 2011), which limits the biological interpretation of a common OTU definition. A distance-based definition of a taxonomic unit

based on the 16S rDNA or full-genome sequences is operational and not necessarily grounded in biological theory (Yarza et al., 2014; Barco et al., 2020). One benefit of a distance-based OTU definition is the ability to mask residual sequencing errors. The sequences generated in microbiome studies can harbor PCR and sequencing errors. These errors would only exacerbate the inflated number of ASVs. Although there are multiple reasons why proponents favor ASVs, we feel the significant risk of artificially splitting genomes into separate clusters is too high to warrant their use.

References
Barco, R., G. Garrity, J. Scott, J. Amend, K. Nealson, and D. Emerson. 2020. A genus definition for bacteria and archaea based on a standard genome relatedness index. mBio 11:e02475-02419.
Dvergedal, H., S. R. Sandve, I. Angell, G. Klemetsdal, and K. Rudi. 2020. Association of gut microbiota with metabolism in juvenile Atlantic salmon. Microbiome 8:160.
Konstantinidis, K. T., and J. M. Tiedje. 2005. Towards a genome-based taxonomy for prokaryotes. Journal of Bacteriology 187:6258-6264.
Pinna, N. K., R. M. Anjana, S. Saxena, A. Dutta, V. Gnanaprakash, G. Rameshkumar, S. Aswath, S. Raghavan, C. S. S. Rani, and V. Radha. 2021. Trans-ethnic gut microbial signatures of prediabetic subjects from India and Denmark. Genome Medicine 13:36.
Sanford, R. A., K. G. Lloyd, K. T. Konstantinidis, and F. E. Löffler. 2021. Microbial taxonomy run amok. Trends in Microbiology 29:394-404.
Schloss, P. D. 2021. Amplicon sequence variants artificially split bacterial genomes into separate clusters. mSphere Jul 21:e0019121. doi: 10.1128/mSphere.00191-21.
Schloss, P. D., and S. L. Westcott. 2011. Assessing and improving methods used in operational taxonomic unit-based approaches for 16S rRNA gene sequence analysis. Applied and Environmental Microbiology 77:3219-3226.
Wen, C., W. Yan, C. Mai, Z. Duan, J. Zheng, C. Sun, and N. Yang. 2021. Joint contributions of the gut microbiota and host genetics to feed efficiency in chickens. Microbiome 9:126.
Yarza, P., P. Yilmaz, E. Pruesse, F. O. Glöckner, W. Ludwig, K.-H. Schleifer, W. B. Whitman, J. Euzéby, R. Amann, and R. Rosselló-Móra. 2014. Uniting the classification of cultured and uncultured bacteria and archaea using 16S rRNA gene sequences. Nature Reviews Microbiology 12:635-645.

Line 157 - what are "singleton OTUs"? OTUs with only one read? OTUs found in only one sample?
Response: "singleton OTUs" represent OTUs found in only one sample (Lines 483-484).
"Singleton OTUs (OTUs found in only one sample) that did not match the reference database were removed."

Lines 159-161 - why were low abundance OTUs discarded? Does it affect your analysis? In general, it is better not to modify the data base extensively. Specifically, some phylogenetic groups are represented by multiple low abundant strains with a high total abundance whereas other phylogenetic groups are represented by a single highly abundant strain. By getting rid of such lower abundance OTUs you might be creating an artifact.
Response: Thank you for the helpful comments. Errors introduced by next-generation amplicon sequencing tend to induce spurious OTUs and spurious counts in OTU tables, both of which are especially prevalent at low abundances. Despite the power of NGS and the progress achieved, generated data is imperfect, being subject to different types of errors, including those inherent to PCR amplification (substitutions and chimeric sequence formation) and sequencing-specific biases which are characteristic of each sequencing technology. Sequencing errors are predominantly caused by base substitutions, although base deletions, low-quality reads, variable read lengths and non-target amplification are also known error sources which may result in false species affiliation. Undetected chimeric sequences, caused by the hybridization of DNA fragments from different species also reduce the reliability of the 16S rDNA sequence-based phylogenetic composition of microbial communities. Together, these different errors generate a high number of lower-abundance sequences, which lead to overestimations of actual community diversity and the creation of many false taxa. Those spurious sequences are hard to filter out using current mainstream pipelines implementing error correction, denoising, and stringent filtration of chimeric sequences, contaminants and non-bacterial contents. Previous study have reported that although the overall abundance of these pseudo sequences was low, introducing them into analysis increased the total number of features to 10 times higher than expected and enlarged the divergence of the alpha and beta diversity analyses among the different methods (Wang et al., 2018). Lower-abundance and lower-quality sequences were observed to surround higher-abundance, biologically real sequences, forming error clouds (Bokulich et al., 2013; Edgar, 2013). Various researchers have developed different approaches to remove these pseudo sequences.
In our work, we described the numerical distribution of OTUs with different existing ratios in samples, after which the slope fluctuation is estimated. We considered there are a lot of false positives for these

microbiotas when fluctuations are great. Therefore, we selected OTUs that existed in more than 20% of samples according to the slope distribution curve (Supplementary Fig. S1a, b). This filtering rule will improve the stability and accuracy of further analyses. Similar filtering rules have also been reported in previous analyses (Zierer et al., 2018). In addition, we have cited Supplementary Fig. S1a, b in the revised manuscript (Lines 488-489).

References

Bokulich, N. A., S. Subramanian, J. J. Faith, D. Gevers, J. I. Gordon, R. Knight, D. A. Mills, and J. G. Caporaso. 2013. Quality-filtering vastly improves diversity estimates from Illumina amplicon sequencing. Nature Methods 10:57-59.
Edgar, R. C. 2013. UPARSE: highly accurate OTU sequences from microbial amplicon reads. Nature Methods 10:996-998.
Wang, J., Q. Zhang, G. Wu, C. Zhang, M. Zhang, and L. Zhao. 2018. Minimizing spurious features in 16S rRNA gene amplicon sequencing. PeerJ Preprints 6:e26872v1 https://doi.org/10.7287/peerj.preprints.26872v1
Zierer, J., M. A. Jackson, G. Kastenmüller, M. Mangino, T. Long, A. Telenti, R. P. Mohney, K. S. Small, J. T. Bell, and C. J. Steves. 2018. The fecal metabolome as a functional readout of the gut microbiome. Nature Genetics 50:790-795.

Lines 378-382 - this is an interesting idea that you did not establish well. Could you determine unifrac (or any other metric) distances of samples from different organs. i.e. compare all vagina samples to all small intestine samples and determine average Unifrac distances and stDev, and do so for each pair of organ sites. Perhaps you will gain a new figure showing how similar or different are the communities in each site.
Response: We are grateful for the suggestion. As suggested by the reviewer, we added a new figure (Supplementary Fig. S2j) showing the weighted UniFrac distances among the six sites.

Line 387 - cyanobacteria are not likely gut or reproductive tract inhabitants. This is more likely an artifact of identifying feed derived chloroplast DNA. You might want to mention this.
Response: Thanks for your helpful suggestions. The chickens used in our study are fed using corn-soybean-based diets, so the Cyanobacteria (likely an artifact of feed-derived chloroplast DNA) were misidentified as microbes. Additionally, we have revised the sentence in the revised manuscript (Lines 118-120).
"Firmicutes, Proteobacteria, and Cyanobacteria (likely an artifact of feed-derived chloroplast DNA) accounted for 71.45% - 97.86% of all OTUs."

Lines 398-402 - what is the connection between the "well-documented protective role of lactobacillus…" and differences in the levels of lactobacillus between gut and reproductive tract? Does the reproductive tract not deserve protection? Please stick to the data. Do not attach unsupported conclusions. Instead connect the levels of Lactobacillus and pH.
Response: As suggested, we have connected the levels of Lactobacillus and pH in the revised manuscript (Lines 134-139).
"Lactobacillus is thought to inhibit pathogenic bacteria by lowering the environmental pH through lactic acid and hydrogen peroxide production [24]. This genus was highly abundant in the digestive tracts which were characterized by low pH values which strongly limits the growth of most pathogens [25, 26]. In contrast, Lactobacillus was less abundant in the reproductive tract where an alkaline pH is needed to maintain sperm motility [27, 28]."

Lines 407-411 - if I understand correctly, you are implying a connection possibly caused because of the flow of material from different sites. You may want to note that.
Response: Thank you for the helpful comments. Yes, we want to note that, and we have added a sentence in our revised manuscript (Lines 146-147).
"These results imply there is a connection of microbiome communities possibly caused because by the flow of material from different sites."

Line 419-421 - you have reduced a whole field of study to just one "previous study". And in any case I am not sure what you wanted to write here.
Response: We have now included additional references in the revised manuscript (Line 158).

Line 442 - not chloroplast and mitochondria-like microbes but rather true chloroplasts and mitochondria which you misidentified as microbes.
Response: Thanks for your helpful suggestion. We have revised the sentence in the manuscript (Lines

179-180).
"Unidentified Chloroplast (2.94%) and mitochondria-like (2.37%) materials from plant consumption."

Line 447 - does Helicobactor maintain near natural pH and a microaerophilic environment? Is that its role in the gut? Please do not bring up bits and pieces of information if this is not really relevant to your results. What is important is that a possible pathogen was found in your chickens.
Response: Thanks for the comments. In order to infect the gastric mucosa, Helicobacter pylori has to survive in the gastric acidic pH, and Helicobacter pylori has well developed mechanisms to neutralize the effects of acidic pH (Ansari, S. and Y. Yamaoka. 2017. Survival of Helicobacter pylori in gastric acidic territory, helicobacter 22:e12386), that is its role in the gastric tract. In addition, we have reorganized the sentence in the revised manuscript (Lines 183-185).
"As a possible pathogen, Helicobacter specifically inhabits the small intestine in chickens, and may be involved in inflammation, metabolism, and neutralization of gastric acid [35-37]."

Lines 450-463 - this whole paragraph contains a lot of disjoined bits and pieces of information. Does it really matter that some bacteria were changed in immune suppressed honey bees? What are you trying to say? Consider taking this whole paragraph off.
Response: Thanks for the comment. This whole paragraph mainly displays the results of the site-associated bacterial taxa in the three reproductive tract sites identified by LEfSe and discussed association with the previous literature. Additionally, we have deleted the disjoined bits and pieces of information and reorganized the sentence in the paragraph in our revised manuscript (Lines 186-194).
"Unidentified Erysipelotrichaceae showed higher abundance in the three reproductive tract sites (1.83% - 2.40%). Bacteria associated with the isthmus and uterus both showed higher abundances than in the other sites. Several genera (typically, Romboutsia, Fusobacterium, and Clostridium sensu stricto 1) were dominant in the vagina (> 25% of the microbiota) but had lower abundances in the other sites (Fig. 2b). Among these, vaginal Romboutsia could be employed as a predictor for egg number in laying hens [8]. Six Bacteroidetes bacterial taxa were isthmus-associated; Bacteroides species live on host mucus-secreted polysaccharides and this flexible foraging behavior contributes to diversity and stability [38]."

Lines 464-466 - do your results support this speculation? If yes please expend, if not please remove.
Response: Thank you for the suggestion. Lines 464-466 have been removed from the revised manuscript.

Line 492 - 0.21% of total abundance? Of total species?
Response: Yes, it is "of total species". We have reorganized the sentence and clarified in the manuscript (Line 223).
"accounting for 0.21% of the microbiota species tested in the small intestine".

Lines 560-568 - this is a good example of discussion! Here you brought relevant information and created a data based speculation.
Response: Thank you for your positive remarks about this discussion.

Line 646 - what do you mean in "microbiome succession"?
Response: "microbiome succession" has been changed to "microbial community" in the revised manuscript (Line 390).

Supplementary figure 2 contains two separate topics - functional sequencing data and bacterial community analysis. Please divide into separate figures.
Response: As suggested, we have divided Supplementary figure 2 into two separate figures (Supplementary Fig. S2 and S11).

Reviewer #2: This is a very good study to which I have only a few comments, mostly to microbiota part in which I am stronger than in chicken genetics. The only weaker part, but not weak, is the fact that the study was performed with only a single flock and was not repeated with different hens at all. I understand that it is impossible to repeat the study in another flock but for example you could have set up Cl. leptum PCR and check for its presence in reproductive tract in completely different birds.
Response: We appreciate the positive feedback and sincerely thank the reviewer for the thoughtful and supportive recommendations, which are of great help in improving the quality of this manuscript.

Please consider the following points.
line 94, indeed laparotomy?
Response: "After laparotomy" has been changed to "After the abdomen was opened" in the revised

manuscript (Line 417).

l.111 and 114, I do not understand wha PCR kit you used. Please, reword.
Response: The PCR amplification was performed using Phusion® High-Fidelity PCR Master Mix (New England Biolabs, Ipswich, Ma, USA). We have revised the sentence in the revised manuscript (Lines 436-439).
"The V4 hypervariable region of the bacterial 16S rDNA was amplified using Phusion® High-Fidelity PCR Master Mix (New England Biolabs, Ipswich, Ma, USA) and the universal primers 515 F (5′-GTGCCAGCMGCCGCGGTAA-3′) and 806 R (5′-GGACTACHVGGGTWTCTAAT -3′) [56]."

l.253, 254, this sentence is somewhat compromised. Please check and reword.
Response: Thank you for the comments, and we have revised the sentence in the revised manuscript (Lines 578-579).
"In addition, we further removed the SNPs with adjacent distances ≤ 5."

l.342, rather verified, or confirmed, then detected. By the way, I am not sure whether you used data from the qPCR in the rest of the manuscript
Response: We have changed "detected" to "verified" in the revised manuscript (Line 664). We only used qPCR data in the "RNA sequencing (RNA-seq) analysis" section.

l.388, these are not Cyanobacteria, this is chloroplast DNA from plants in the feed, you may check very recent paper Volf et al. Eggshell and Feed Microbiota Do Not Represent Major Sources of Gut Anaerobes for Chickens in Commercial Production. Microorganisms 2021, 9, 1480.
Response: Thanks for the valuable suggestions, and we have read the above reference carefully. The chickens used in our study are fed using corn-soybean-based diets, so the Cyanobacteria (likely an artifact of feed derived-chloroplast DNA) was misidentified as microbes. Additionally, we have revised the sentence in the manuscript (Lines 121-122).
"Cyanobacteria (likely an artifact of feed-derived chloroplast DNA) was the dominant material in the gizzard (48.19% of the total abundance)."

l.462,463, I would suggest alternative explanation in this case and this is living on expense of host mucus secreted polysaccharides, Sonnenburg, J. L. et al. Glycan foraging in vivo by an intestine-adapted bacterial symbiont. Science 307, 1955-1959 (2005)
Response: As suggested, we have cited the reference and revised the sentence in the manuscript (Lines 192-194).
"Bacteroides species live on host mucus-secreted polysaccharides and this flexible foraging behavior contributes to diversity and stability [38]."

l.559, check that B. fragilis might be a consequence of intensive human care, Kollarcikova et al. Different Bacteroides Species Colonise Human and Chicken Intestinal Tract. Microorganisms 2020, 8, 1483.
Response: We are grateful for the suggestion. We have cited the useful and interesting reference in the revised manuscript (Lines 301-303).
"Interestingly, a recent study reported that the human-adapted Bacteroides species are likely introduced to chicken flocks by contact with humans and can temporarily persist in chickens [49]."

l.615, how can you know this? What if all of this the other way round, and I indeed believe that this is the other way, i.e. hens becomes of compromised performance, due to whatever factor, within but possibly also outside of those which you have monitored. This naturally results in decrease in egg lay but also in increased inflammatory response. Locally changed conditions due to inflammatory signaling change, infiltrating heterophils and macrophages produce antimicrobial peptides and reactive oxygen species and strict anaerobes will be the firs bacterial species to decrease in a response of increasing oxygen concentration. What is cause and what is consequence. I do not know, I think that you do not know either, though you blame bacteria that these are responsible for the response.
Response: Thank you for the comments. We deleted the sentence that caused misunderstanding.

l.631, similar to previous comment, I do not think that there is any downregulation. In high egg producers, there is basal, background expression of inflammatory marker genes. And these are induced in the hens with compromised performance. Be also careful, whether this induction since this could also be a cause of infiltration of macrophage with their specific expression profile, and you then mistakenly conclude on induction when purifying mRNA from a total complex tissue.
Response: Thanks for your comments. "downregulated" has been changed to "different expressed" in

the revised manuscript (Line 383).

l.656, the same as above, be careful what is cause and what is consequence. Increase in reactive oxygen species may affect the most strict anaerobes. When these are present, there is no inflammation. When these are eliminated by increase in oxygen concentration and all other inflammatory responses, this is explained that these bacteria are anti-inflammatory. These are not, these only dislike inflammation and oxygen.
Response: Thanks for your helpful suggestion. We have deleted the sentence in the revised manuscript.

Close
