# Supplementary material for: Association of female reproductive tract microbiota with egg production in layer chickens
Source: Gigascience. 2021 Sep 23;10(9):giab067. doi: 10.1093/gigascience/giab067 (PMC8460357; doi:10.1093/gigascience/giab067)
Supplement: giab067_Reviewer_1_Report_Original_Submission — Erez Mills -- 6/14/2021 Reviewed [file giab067_reviewer_1_report_original_submission.pdf]

Reviewer Report

Title: Female reproductive tract microbiota influence egg production in layer chickens

Version: Original Submission **Date:** 6/14/2021

Reviewer name: Erez Mills

Reviewer Comments to Author:

Dear authors, the work described in the manuscript is very extensive! I have not yet seen an analysis of host genetics, microbiota composition and host transcriptomics coupled with egg production data. Also while not perfect the manuscript is written well.

Main problems:

1. It is clear you found an association between specific bacteria and egg production. You have also shown that some form of inflammation is associated with the changes in microbiota and egg production. However, you assume that the bacteria affect inflammation which affect egg production. But other options also exist. For example, it is possible that waning egg production, from other causes, changes the conditions in the oviduct so that the microbiota would change. Or inflammation, for example because of a pathogen, might modulate both egg production as well as microbiota composition. Please change the text so that the readers understand you are speculating and briefly mention the other options.
2. While it is too late to change, it is a pity that you did not characterize the cecum community. Of all of the intestinal communities the cecum is by far the biggest and the most likely to affect the nutrition of the hen, thereby possibly affecting egg production.
3. Please make sure to reference and discuss relevant literature. I quickly identified Elokil A. A. Animal 2020 which performed a limited but similar analysis. Please perform a literature search and make sure to reference and discuss relevant work.

Minor problems:

Lines 48-49 - something is wrong with this sentence.

Lines 67-69 - it is not clear what you are trying to say here.

Line 71 - 16S rDNA sequencing and not whole-genome sequencing.

Line 96 - please be more specific regarding which part of the small intestine was sampled.

Lines 100-108 - description of host DNA extraction is not appropriate another the heading "microbial genomic DNA extraction".

Line 109 and elsewhere - 16S rDNA and not 16S rRNA.

Line 154 - why are you using OTUs with 97% identity and not 100% identical amplicon sequence variants (ASVs)?

Line 157 - what are "singleton OTUs"? OTUs with only one read? OTUs found in only one sample?

Lines 159-161 - why were low abundance OTUs discarded? Does it affect your analysis? In general, it is better not to modify the data base extensively. Specifically, some phylogenetic groups are represented by multiple low abundant strains with a high total abundance whereas other phylogenetic groups are represented by a single highly abundant strain. By getting rid of such lower abundance OTUs you might

be creating an artifact.
Lines 378-382 - this is an interesting idea that you did not establish well. Could you determine unifrac (or any other metric) distances of samples from different organs. i.e. compare all vagina samples to all small intestine samples and determine average Unifrac distances and stDev, and do so for each pair of organ sites. Perhaps you will gain a new figure showing how similar or different are the communities in each site.
Line 387 - cyanobacteria are not likely gut or reproductive tract inhabitants. This is more likely an artifact of identifying feed derived chloroplast DNA. You might want to mention this.
Lines 398-402 - what is the connection between the "well-documented protective role of lactobacillus…" and differences in the levels of lactobacillus between gut and reproductive tract? Does the reproductive tract not deserve protection? Please stick to the data. Do not attach unsupported conclusions. Instead connect the levels of Lactobacillus and pH.
Lines 407-411 - if I understand correctly, you are implying a connection possibly caused because of the flow of material from different sites. You may want to note that.
Line 419-421 - you have reduced a whole field of study to just one "previous study". And in any case I am not sure what you wanted to write here.
Line 442 - not chloroplast and mitochondria-like microbes but rather true chloroplasts and mitochondria which you misidentified as microbes.
Line 447 - does Helicobactor maintain near natural pH and a microaerophilic environment? Is that its role in the gut? Please do not bring up bits and pieces of information if this is not really relevant to your results. What is important is that a possible pathogen was found in your chickens.
Lines 450-463 - this whole paragraph contains a lot of disjoined bits and pieces of information. Does it really matter that some bacteria were changed in immune suppressed honey bees? What are you trying to say? Consider taking this whole paragraph off.
Lines 464-466 - do your results support this speculation? If yes please expend, if not please remove.
Line 492 - 0.21% of total abundance? Of total species?
Lines 560-568 - this is a good example of discussion! Here you brought relevant information and created a data based speculation.
Line 646 - what do you mean in "microbiome succession"?
Supplementary figure 2 contains two separate topics - functional sequencing data and bacterial community analysis. Please divide into separate figures.

Methods

Are the methods appropriate to the aims of the study, are they well described, and are necessary controls included? Choose an item.

Conclusions

Are the conclusions adequately supported by the data shown? Choose an item.

Reporting Standards

Does the manuscript adhere to the journal's guidelines on [minimum standards of reporting?] Choose an item.

Choose an item.

Statistics

Are you able to assess all statistics in the manuscript, including the appropriateness of statistical tests used? Choose an item.

Quality of Written English

Please indicate the quality of language in the manuscript: Choose an item.

Declaration of Competing Interests

Please complete a declaration of competing interests, considering the following questions:

- Have you in the past five years received reimbursements, fees, funding, or salary from an organisation that may in any way gain or lose financially from the publication of this manuscript, either now or in the future?
- Do you hold any stocks or shares in an organisation that may in any way gain or lose financially from the publication of this manuscript, either now or in the future?
- Do you hold or are you currently applying for any patents relating to the content of the manuscript?
- Have you received reimbursements, fees, funding, or salary from an organization that holds or has applied for patents relating to the content of the manuscript?
- Do you have any other financial competing interests?
- Do you have any non-financial competing interests in relation to this paper?

If you can answer no to all of the above, write 'I declare that I have no competing interests' below. If your reply is yes to any, please give details below.

I declare that I have no competing interests

I agree to the open peer review policy of the journal. I understand that my name will be included on my report to the authors and, if the manuscript is accepted for publication, my named report including any attachments I upload will be posted on the website along with the authors' responses. I agree for my report to be made available under an Open Access Creative Commons CC-BY license (http://creativecommons.org/licenses/by/4.0/). I understand that any comments which I do not wish to be included in my named report can be included as confidential comments to the editors, which will not be published.

Choose an item.

To further support our reviewers, we have joined with Publons, where you can gain additional credit to further highlight your hard work (see: https://publons.com/journal/530/gigascience). On publication of this paper, your review will be automatically added to Publons, you can then choose whether or not to claim your Publons credit. I understand this statement.

Yes Choose an item.
