# Supplementary material for: Association of female reproductive tract microbiota with egg production in layer chickens
Source: Gigascience. 2021 Sep 23;10(9):giab067. doi: 10.1093/gigascience/giab067 (PMC8460357; doi:10.1093/gigascience/giab067)
Supplement: giab067_Reviewer_2_Report_Original_Submission — Ivan Rychlik -- 7/11/2021 Reviewed [file giab067_reviewer_2_report_original_submission.pdf]

Reviewer Report

Title: Female reproductive tract microbiota influence egg production in layer chickens

Version: Original Submission **Date:** 7/11/2021

Reviewer name: Ivan Rychlik

Reviewer Comments to Author:

This is a very good study to which I have only a few comments, mostly to microbiota part in which I am stronger than in chicken genetics. The only weaker part, but not weak, is the fact that the study was performed with only a single flock and was not repeated with different hens at all. I understand that it is impossible to repeat the study in another flock but for example you could have set up Cl. leptum PCR and check for its presence in reproductive tract in completely different birds.
Please consider the following points.
line 94, indeed laparotomy?
l.111 and 114, I do not understand wha PCR kit you used. Please, reword.
l.253, 254, this sentence is somewhat compromised. Please check and reword.
l.342, rather verified, or confirmed, then detected. By the way, I am not sure whether you used data from the qPCR in the rest of the manuscript
l.388, these are not Cyanobacteria, this is chloroplast DNA from plants in the feed, you may check very recent paper Volf et al. Eggshell and Feed Microbiota Do Not Represent Major Sources of Gut Anaerobes for Chickens in Commercial Production. Microorganisms 2021, 9, 1480.
l.462,463, I would suggest alternative explanation in this case and this is living on expense of host mucus secreted polysaccharides, Sonnenburg, J. L. et al. Glycan foraging in vivo by an intestine-adapted bacterial symbiont. Science 307, 1955-1959 (2005)
l.559, check that B. fragilis might be o consequence of intensive human care, Kollarcikova et al. Different Bacteroides Species Colonise Human and Chicken Intestinal Tract. Microorganisms 2020, 8, 1483.
l.615, how can you know this? What if all of this the other way round, and I indeed believe that this is the other way, i.e. hens becomes of compromised performance, due to whatever factor, within but possibly also outside of those which you have monitored. This naturally results in decrease in egg lay but also in increased inflammatory response. Locally changed conditions due to inflammatory signaling change, infiltrating heterophils and macrophages produce antimicrobial peptides and reactive oxygen species and strict anaerobes will be the firs bacterial species to decrease in a response of increasing oxygen concentration. What is cause and what is consequence. I do not know, I think that you do not know either, though you blame bacteria that these are responsible for the response.
l.631, similar to previous comment, I do not think that there is any downregulation. In high egg producers, there is basal, background expression of inflammatory marker genes. And these are induced in the hens with compromised performance. Be also careful, whether this induction since this could also be a cause of infiltration of macrophage with their specific expression profile, and you then mistakenly conclude on induction when purifying mRNA from a total complex tissue.
l.656, the same as above, be careful what is cause and what is consequence. Increase in reactive oxygen

species may affect the most strict anaerobes. When these are present, there is no inflammation. When these are eliminated by increase in oxygen concentration and all other inflammatory responses, this is explained that these bacteria are anti-inflammatory. These are not, these only dislike inflammation and oxygen.

Methods

Are the methods appropriate to the aims of the study, are they well described, and are necessary controls included? Choose an item.

Conclusions

Are the conclusions adequately supported by the data shown? Choose an item.

Reporting Standards

Does the manuscript adhere to the journal's guidelines on minimum standards of reporting? Choose an item.

Choose an item.

Statistics

Are you able to assess all statistics in the manuscript, including the appropriateness of statistical tests used? Choose an item.

Quality of Written English

Please indicate the quality of language in the manuscript: Choose an item.

Declaration of Competing Interests

Please complete a declaration of competing interests, considering the following questions:

- Have you in the past five years received reimbursements, fees, funding, or salary from an organisation that may in any way gain or lose financially from the publication of this manuscript, either now or in the future?
- Do you hold any stocks or shares in an organisation that may in any way gain or lose financially from the publication of this manuscript, either now or in the future?
- Do you hold or are you currently applying for any patents relating to the content of the manuscript?
- Have you received reimbursements, fees, funding, or salary from an organization that holds or has applied for patents relating to the content of the manuscript?
- Do you have any other financial competing interests?

- Do you have any non-financial competing interests in relation to this paper?

If you can answer no to all of the above, write 'I declare that I have no competing interests' below. If your reply is yes to any, please give details below.

I declare that I have no competing interests.

I agree to the open peer review policy of the journal. I understand that my name will be included on my report to the authors and, if the manuscript is accepted for publication, my named report including any attachments I upload will be posted on the website along with the authors' responses. I agree for my report to be made available under an Open Access Creative Commons CC-BY license (http://creativecommons.org/licenses/by/4.0/). I understand that any comments which I do not wish to be included in my named report can be included as confidential comments to the editors, which will not be published.

Choose an item.

To further support our reviewers, we have joined with Publons, where you can gain additional credit to further highlight your hard work (see: https://publons.com/journal/530/gigascience). On publication of this paper, your review will be automatically added to Publons, you can then choose whether or not to claim your Publons credit. I understand this statement.

Yes Choose an item.
